# High abundance of Serine/Threonine-rich regions predicted to be hyper-*O*-glycosylated in the secretory proteins coded by eight fungal genomes

**DOI:** 10.1186/1471-2180-12-213

**Published:** 2012-09-20

**Authors:** Mario González, Nélida Brito, Celedonio González

**Affiliations:** 1 Departamento de Bioquímica y Biología Molecular, Universidad de La Laguna, La Laguna (Tenerife), E-38206, Spain

## Abstract

**Background:**

*O*-glycosylation of secretory proteins has been found to be an important factor in fungal biology and virulence. It consists in the addition of short glycosidic chains to Ser or Thr residues in the protein backbone via *O*-glycosidic bonds. Secretory proteins in fungi frequently display Ser/Thr rich regions that could be sites of extensive *O*-glycosylation. We have analyzed *in silico* the complete sets of putatively secretory proteins coded by eight fungal genomes (*Botrytis cinerea*, *Magnaporthe grisea*, *Sclerotinia sclerotiorum*, *Ustilago maydis*, *Aspergillus nidulans*, *Neurospora crassa*, *Trichoderma reesei*, and *Saccharomyces cerevisiae*) in search of Ser/Thr-rich regions as well as regions predicted to be highly *O*-glycosylated by NetOGlyc (http://www.cbs.dtu.dk).

**Results:**

By comparison with experimental data, NetOGlyc was found to overestimate the number of *O-*glycosylation sites in fungi by a factor of 1.5, but to be quite reliable in the prediction of highly *O-*glycosylated regions. About half of secretory proteins have at least one Ser/Thr-rich region, with a Ser/Thr content of at least 40% over an average length of 40 amino acids. Most secretory proteins in filamentous fungi were predicted to be *O*-glycosylated, sometimes in dozens or even hundreds of sites. Residues predicted to be *O*-glycosylated have a tendency to be grouped together forming hyper-*O*-glycosylated regions of varying length.

**Conclusions:**

About one fourth of secretory fungal proteins were predicted to have at least one hyper-*O*-glycosylated region, which consists of 45 amino acids on average and displays at least one *O-*glycosylated Ser or Thr every four residues. These putative highly *O*-glycosylated regions can be found anywhere along the proteins but have a slight tendency to be at either one of the two ends.

## Background

It has been estimated that more than half of all proteins are glycoproteins [1], a proportion expected to be much higher if only secretory proteins are considered. The term secretory will be used in this article as comprising all proteins entering the secretory pathway, i.e. all proteins having a signal peptide. Glycosyl residues, mainly N-acetylgalactosamine, mannose, galactose or glucose, can be linked to proteins via asparagine (N-glycosylation) or via hydroxylated amino acids including serine, threonine, and, more rarely, tyrosine, hydroxyproline and hydroxylysine (*O*-glycosylation) [2,3]. The first step of *O*-glycosylation in fungi generally consists in the addition of 1–3 mannose units from dolichyl phosphate mannose to Ser/Thr residues in target proteins [3], by the action of protein *O*-mannosyltransferases (PMTs) in the endoplasmic reticulum. The initial addition of glucose or galactose residues to Ser/Thr has also been reported for *Trichoderma*[2]. The chain is then extended, as the protein continues the secretion through Golgi, by several other enzymes generating linear or branched sugar chains composed mostly of mannose residues. Yeast usually have linear sugar chains composed exclusively of mannose [4], but filamentous fungi may have branched chains containing also glucose or galactose [2,3].

The physiological function of *O*-glycosylation has been established mostly by analyzing null mutants in one or more PMT genes, which show a reduced ability to add sugars to Ser/Thr residues in the secretion pathway. A role for *O*-glycosylation could be established in enhancing the stability and solubility of the proteins, in protecting from proteases, as a sorting determinant, and in the development and differentiation of the fungal hyphae [2]. It is common that the knock-out of a particular PMT gene, or the simultaneous deletion of several of them, causes loss of viability or strong defects such as lower conidiation, changes in fungal morphology, etc. [2], emphasizing the importance of *O*-glycosylation for the biology of fungal organisms. Regarding phytopathogenic fungi, it is very interesting the fact that the deletion of an individual PMT gene in *Ustilago maydis* completely abolished pathogenicity of the fungus by eliminating the ability to penetrate the plant tissue, without otherwise affecting the *U. maydis* life cycle [5,6]. Additionally, *O*-glycosylation may play an important role in the regulation of enzymatic activity, as has been shown for the *Aspergillus awamori* Gluco-amylase, which has a Ser/Thr-rich domain that carries several *O*-linked oligomannose structures necessary for the activity of the enzyme against raw, but not against dissolved, starch [7].

In metazoans, mucin-type *O*-glycosylation sites are found grouped in clusters in protein regions rich in Ser and Thr residues [8]. Proteins containing mucin-like *O*-glycosylation are often found bound to the plasma membrane constituting the glycocalyx, or in the extracellular medium contributing to the formation of the extracellular matrix or the gel-like mucus in the mucosal surfaces. Mucins seem to be restricted to metazoans, where they appeared soon in evolution [9], and *in silico* analysis has been applied to the identification of mucins in animal species with sequenced genomes [9,10]. To our knowledge, a similar approach has never been used in fungi despite the fact that fungal secretory proteins are frequently highly glycosylated and contain Ser/Thr-rich regions predicted to be the site of high density *O*-glycosylation of the polypeptide chains [11]. Here we have analyzed *in silico* the presence and distribution of such regions among the putatively secretory proteins coded by the genomes of *S. cerevisiae*, four plant-pathogenic filamentous fungi (*Botrytis cinerea, Magnaporthe grisea, Sclerotinia sclerotiorum* and *Ustilago maydis*) and three non-pathogenic filamentous fungi (*Aspergillus nidulans*, *Neurospora crassa* and *Trichoderma reesei*). The results show a high frequency of Ser/Thr rich regions in the secretory proteins for all the fungi studied, as well as the prediction of regions highly *O*-glycosylated for about 25% of them.

## Results

### NetOGlyc 3.1 can predict regions with a high density of *O*-glycosylation in fungal proteins

Part of the results presented here relies on the prediction of *O*-glycosylation by the web-based server NetOGlyc 3.1 [12,13]. This tool consists of a Neural Network trained on mucin-type mammalian *O*-glycosylation sites (O-N-acetylgalactosamine) and thus has not been designed to predict fungal *O*-glycosylation sites (mainly O-mannose). In order to check the usefulness of NetOGlyc for fungal proteins, we used all the available fungal proteins with experimentally confirmed *O*-glycosylation sites that were produced in their natural host, only 30 to our knowledge (Additional file 1), and compared them with the predictions of NetOGlyc for the same group of proteins. NetOGlyc predicted a total of 288 *O*-glycosylation sites for the whole set, while the number of experimentally-determined *O*-glycosylation sites was 197. The number of sites predicted by NetOGlyc that were actually found experimentally was 106. Therefore, the server accurately predicts 54% of the experimentally-found *O*-glycosylation sites, which is not much worse than the value of 67% found for the same parameter with mammalian proteins, for which the system was designed [12]. However, NetOGlyc seems to produce a higher rate of false positives for fungal proteins than for mammalian proteins and therefore overestimates the number of *O*-glycosylation sites. The parameter defined as specificity (the fraction of all positive predictions that are correct) by Julenius et al. [12] showed a value of 37% for fungal proteins while it was 68% for mammalian proteins. Although these differences are certainly not small, the accuracy of NetOGlyc with fungal proteins is, in our opinion, higher than what one could expect from the poor conservation in the molecular mechanisms involved in protein *O*-glycosylation between fungi and mammals [14]. The relationship between the number of experimental vs. predicted *O*-glycosylation sites, 197 divided by 288, was used to correct the statistics about fungal proteins calculated from NetOGlyc results, such as the average number of *O*-glycosylation sites per protein, to compensate the overestimation produced by NetOGlyc. The number of predicted *O*-glycosylation sites multiplied by 0.68 was therefore taken as a rough estimation of the actual number of *O*-glycosylation sites.

**Figure 1:**
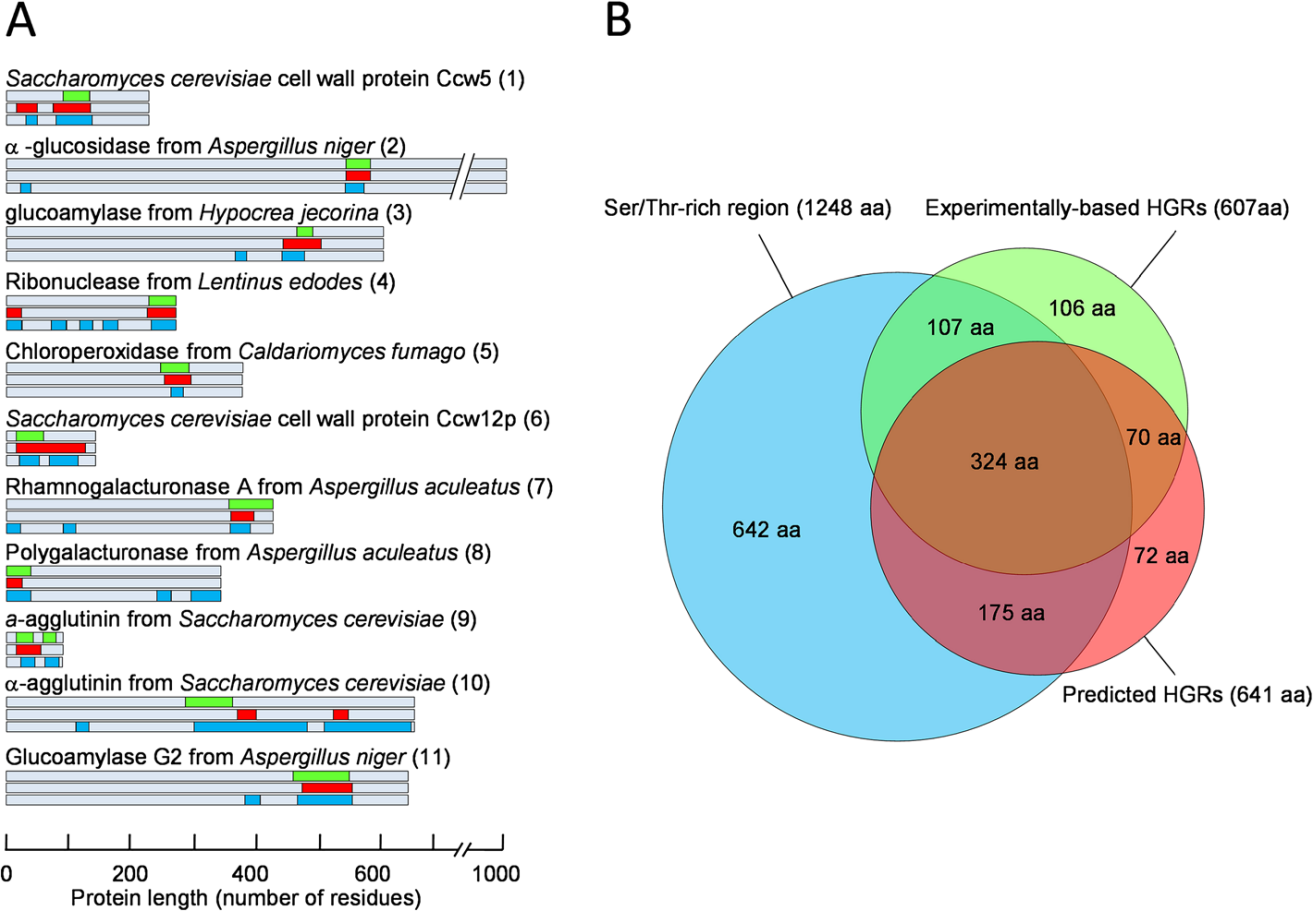
**Comparison of experimentally confirmed HGRs with those predicted by NetOGlyc (pHGRs) and with Ser/Thr-rich regions in the same set of proteins. A**: Experimental HGRs are represented as green boxes and pHGRs as red boxes. Ser/Thr-rich regions are represented as blue boxes. HGRs have a minimum of 15% *O*-glycosylated residues in the case of the experimental data, or 25% in the case of NetOGlyc-predicted *O*-glycosylation sites (to correct for the overestimation produced by NetOGlyc). Ser/Thr rich regions have a minimum Ser/Thr content of 40%. Numbers in brackets identify these proteins in Additional file 1, with more information for each of them including references. **B**: Venn diagram displaying the number of amino acid coincidences in the three kinds of regions. Each area is proportional to the number of amino acids (also displayed in the figure) which are inside a given type of region (or in several of them simultaneously) for the whole protein set.

Despite its relatively poor prediction of individual *O*-glycosylation sites, NetOGlyc showed a much higher accuracy in the prediction of highly *O*-glycosylated regions (HGRs), defined as regions not smaller than 20 amino acids of which at least 25% are *O-*glycosylated Ser or Thr residues. Details about how HGRs are calculated can be found in the Materials and Methods section. Figure 1A shows HGRs found in the set of proteins with experimentally determined *O*-glycosylation sites. Almost all of them were also predicted by NetOGlyc. The reason for this increase in performance could be related to the fact that these hyper-*O*-glycosylated regions need to be also Ser/Thr-rich regions, which are predicted to be hyper-*O*-glycosylated both in mammals and in fungi, only that in fungi the exact *O*-glycosylated site is somehow predicted in the wrong amino acids. To assess this possibility we also studied the presence of Ser/Thr-rich regions in the control set of proteins, defined as protein regions with a minimum Ser/Thr content of 40% over a window of at least 20-aa (Figure 1A). The results showed that actually most experimental HGRs are also rich in Ser/Thr. However, when we explored numerically the overlap between experimental HGRs and predicted HGRs (pHGRs) or Ser/Thr–rich regions (Figure 1B), we observed that NetOGlyc did a better job at predicting *O*-glycosylation-rich regions than the mere determination of Ser/Thr content. We can summarize the data in Figure 1B by saying that an amino acid within a pHGR, predicted by NetOGlyc, has a probability of 0.61 of being inside a real HGR, while the same probability is just 0.34 for an amino acid within a Ser/Thr-rich region. We can conclude, therefore, that NetOGlyc, although being of limited use in the prediction of single *O*-glycosylation sites in fungal proteins, can be effective in the prediction of highly *O*-glycosylated regions, which is the aim of this work.

### Fungal signalP-positive proteins frequently display Ser/Thr-rich regions

As a first step in the study of *O*-glycosylation in fungal secretory proteins, we determined the set of proteins for which a signal peptide was predicted by SignalP (Additional file 2), for the 8 genomes analyzed in this study. The number of putatively secretory proteins varied widely, with the maximum number being displayed by *M. grisea* and the minimum corresponding to *S. cerevisiae* (Table 1). No clear relationship was observed between the number of proteins entering the secretory pathway by any given fungus and their biology. Phytopathogenic fungi, for example, seem to have a tendency to have a slightly higher number of proteins predicted to have signal peptide, but *U. maydis* is a clear counterexample.

**Table 1:** Predictions obtained from SignalP and NetOGlyc for the proteins coded by the eight fungal genomes

**Organism**	**Total number of proteins**	**Predicted to have signal peptide**^**a**^	**Predicted to have signal peptide and to be*O*-Glycosylated**^**b**^
*Botrytis cinerea* T4	16360	1910 (11.7%)	1146 (60.0%)
*Magnaporthe grisea*	11109	2023 (18.2%)	1400 (69.2%)
*Sclerotinia sclerotiorum*	14522	1551 (10.7%)	913 (58.9%)
*Ustilago maydis*	9129	837 (12.8%)	603 (72.0%)
*Aspergillus nidulans*	10560	1453 (13.8%)	932 (64.1%)
*Neurospora crassa*	9907	1250 (12.6%)	929 (74.3%)
*Trichoderma reesei*	9129	1169 (9.2%)	695 (59.5%)
*Saccharomyces cerevisiae*	5900	594 (10.1%)	250 (42.1%)
Global average	10827	1348.4 (12.4%)	858.5 (63.7%)

^a^ As predicted by SignalP, percentages are calculated in relation to the total number of proteins.

^b^ As predicted by SignalP and NetOGlyc, percentages are calculated in relation to the number of proteins predicted to have signal peptide.

We then used the MS Excel macro “XRR”, explained in the Material and Methods section, to identify Ser/Thr-rich regions among all the SignalP-positive proteins coded by the eight fungal genomes. The parameters used in the analysis (W = 20, %G = 40, S = 5) ensured that all regions found were at least 20-amino acids long and had a minimum Ser/Thr content of 40%. Between 38.1% (*M. grisea*) and the 61.3% (*U. maydis*) of all proteins with predicted signal peptide contain at least one Ser/Thr-rich region (Table 2). Their average length was similar for the 8 genomes, varying between 32.1 residues (*M. grisea*) and 65.4 residues (*S. cerevisiae*), although regions much longer were found for all the organisms. Therefore, about half of fungal proteins with predicted signal peptide show at least one region with a 40%, or more, Ser/Thr content and with an average length of 40.1 amino acids.

**Table 2:** Ser/Thr-rich regions and pHGRs predicted in secretory proteins from the eight fungi

**Organism**	**Ser/Thr-rich regions**				**Predicted hyper-*O*-glycosylated regions**			
	**No. of regions**	**No. of proteins**^**a**^	**Length average**	**Maximal length**	**No. of regions**	**No. of proteins**^**a**^	**Length average**	**Maximal length**
*Botrytis cinerea* T4	1850	966 (50.6%)	41.5	1133	606	434 (22.7%)	45.6	437
*Magnaporthe grisea*	1190	770 (38.1%)	32.1	769	421	543 (26.8%)	36.9	753
*Sclerotinia sclerotiorum*	1502	782 (50.4%)	41.6	1216	512	356 (23%)	45.8	361
*Ustilago maydis*	1037	513 (61.3%)	33.7	618	276	214 (25.6%)	32.3	145
*Aspegillus nidulans*	1202	729 (50.2%)	33.9	499	345	269 (18.5%)	45.9	507
*Neurospora crassa*	1329	714 (57.1%)	35.6	700	538	389 (31.1%)	38.8	622
*Trichoderma reesei*	933	546 (46.7%)	36.6	617	311	233 (19.9%)	52.2	418
*Saccharomyces cerevisiae*	496	265 (44.6%)	65.4	1429	174	108 (18.2%)	66.9	821
Global average	1192.4	660.6 (49%)	40.1	872.6	397.9	318.3 (23.6%)	45.5	508

^**a**^ Values in brackets represent the percentage with respect to the number of secretory proteins.

### Most fungal secretory proteins are predicted to be *O*-glycosylated

We then used the NetOGlyc 3.1 server to detect the presence of potentially *O*-glycosylated Ser/Thr residues in the sets of signalP-positive proteins. A respectable number of proteins showed at least one Ser or Thr residue for which *O*-glycosylation is predicted (Additional file 2). A little less than half of *S. cerevisiae* signalP-positive proteins (42.1%) display at least one *O*-glycosylation, but the percentage is always higher for filamentous fungi, ranging from 58.9% for *Sclerotinia sclerotiorum* to 72.0% for *U. maydis* (Table 1). It is necessary to insist at this point that these numbers refer only to the predictions carried out by NetOGlyc 3.1, which seems to overestimate the actual number of *O*-glycosylation sites (see above).

**Figure 2:**
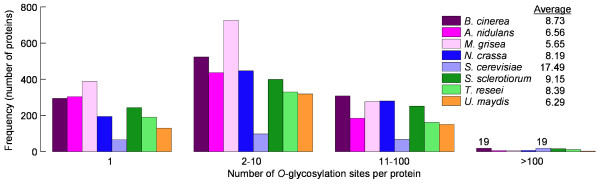
**Frequency distribution of the number of *O*-glycosylation sites per protein predicted by NetOGlyc.** Inset displays the average number of *O*-glycosylated residues per protein, corrected by multiplying by 0.68 to compensate the overestimation of *O*-glycosylated sites produced by the server on fungal proteins. See details in the text.

About 20-30% of *O-*glycosylated proteins are predicted to have sugars added to only one Ser/Thr residue (Figure 2), but most of them have multiple *O*-glycosylation sites reaching dozens or even hundreds of putatively *O*-glycosylated Ser/Thr residues in the same protein, in all the genomes studied. Correcting for the overestimation of the number of *O*-glycosylation sites, as explained above, the average number of *O*-glycosylations per protein is predicted to be in the range 5–10 sites per protein, except in the case of *S. cerevisiae* with a much higher number. This yeast seems therefore to differ clearly from filamentous fungi in the sense that it possesses quite a lower number of *O-*glycosylated proteins (Table 1), only partially explained by the smaller genome size, but they are more extensively *O-*glycosylated (Figure 2).

If we look at individual proteins we can find some with an extremely high number of *O*-glycosylation sites (Additional file 2). The protein with the highest proportion of predicted *O*-glycosylated residues is the *M. grisea* protein MG06773.4, of unknown function, with about half of its 819 amino acids being predicted to be *O-*glycosylated. Next is the *S. cerevisiae* protein YIR019C (Muc1), a mucin-like protein necessary for the yeast to grow with a filamentous pseudohyphal form [15]. Muc1 is a 1367-amino acids protein, of which 42% are predicted to be *O*-glycosylated. Similar examples can be found in the rest of the genomes, with at least a few proteins predicted to have more than 25% of their residues *O-*glycosylated.

### Fungal proteins are rich in pHGRs

The glycosylation positions obtained from NetOGlyc were analyzed with the MS Excel macro XRR in search of *O*-glycosylation-rich regions. The raw results can be found in Additional file 3 and a summary is presented in Table 2. All the genomes analyzed code for plenty of secretory proteins with pHGRs. Between 18% (*S. cerevisiae*) and 31% (*N. crassa*) of all proteins with predicted signal peptide contain at least one pHGR. The average length of pHGRs was similar for the eight genomes, varying between 32.3 residues (*U. maydis*) and 66.9 residues (*S. cerevisiae*), although pHGRs could be found of any length between the minimum, 5 residues, to several hundred. All genomes coded for proteins predicted to have quite large pHGRs, the record being the 821-aa pHGR found in the *S. cerevisiae* protein Muc1 discussed above. Globally, we could summarize these data by saying that among the set of secretory fungal proteins predicted by NetOGlyc to be *O*-glycosylated, about one fourth shows at least one pHGR having a mean length of 23.6 amino acids and displaying, on average, an *O*-glycosylated Ser or Thr residue every four amino acids.

**Figure 3:**
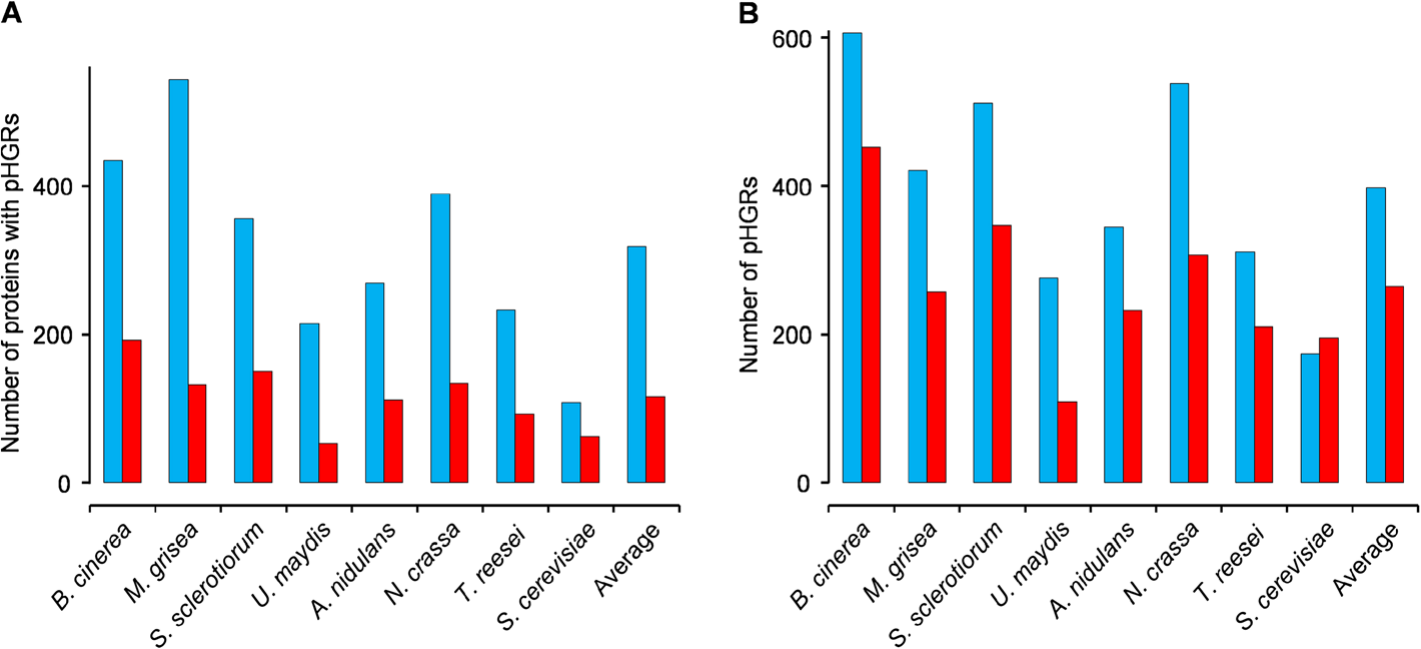
**Effect of the randomization of the position of the *O*-glycosylation sites on pHGR prediction.** Number of proteins with pHGRs (**A**) and total number of pHGRs (**B**) found in every genome with the *O*-glycosylation positions predicted by NetOGlyc (blue columns) or the randomized positions (red columns).

One possible concern about pHGRs is whether they are the result of a real tendency of the predicted *O*-glycosylation sites to be together forming hyper-*O*-glycosylated regions or, on the contrary, they are just a consequence of having proteins with a high number of *O*-glycosylation sites that, being so much, have no choice but be close to one another forming pHGRs. To address this question we randomized the *O*-glycosylation positions for all the proteins. In this new set of data, the proteins displayed the same number of *O*-glycosylation sites as predicted by NetOGlyc but their positions were chosen at random. When these hypothetical proteins were analyzed in search of pHGRs, we obtained the results presented in Figure 3. The number of proteins displaying pHGRs was considerably smaller when the positions of the *O*-glycosylation sites were randomized. Between 42.6% (*S. cerevisiae*) to 75.7% (*M. grisea*) of the proteins displaying pHGRs with the *O*-glycosylation sites predicted by NetOGlyc lost them with the randomization of the *O*-glycosylation positions, indicating that at least in the majority of proteins there is really a selective pressure to localize the *O*-glycosylation sites grouped in pHGRs. The total number of pHGRs was also lower with the randomized data (Figure 3B), although in this case the difference was not so big, and in the case of *S. cerevisiae* the total number of pHGRs actually increased with the randomization of the *O*-glycosylation positions. The reason for this result may be related to the presence of proteins predicted to have a very high number of *O*-glycosylation sites in this yeast, for which the randomization of the *O*-glycosylation positions leads to the scattering of the sites throughout the whole protein and the appearance of a greater number of smaller pHGRs. As discussed before, *S. cerevisiae* differentiates from the rest of the organisms under study in the sense that it possesses a higher proportion of these highly *O*-glycosylated proteins (Figure 2).

### pHGRs show a small tendency to be located at protein ends

**Figure 4:**
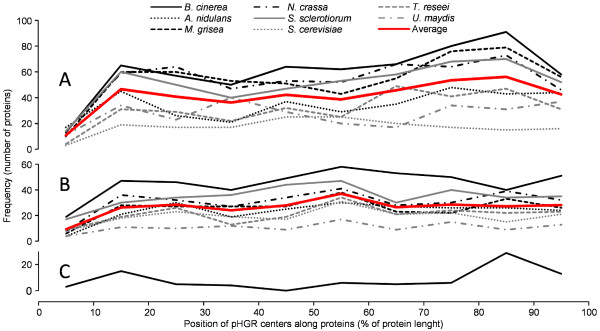
**Distribution of pHGRs along the length of proteins.** For each organism, the relative position of the centers of all pHGRs along the length of their respective protein was calculated, as percent distance from the N-terminus. The graph displays the frequency distribution of these pHGR centers in ten groups. **A**: distribution obtained with the position of *O*-glycosylation sites obtained from NetOGlyc. **B**: distribution obtained when the position of the *O*-glycosylation sites were randomized. **C**: distribution obtained for the group of *B. cinerea* secretory enzymes active on polysaccharides, using the not-randomized *O*-glycosylation positions.

We then addressed the question of whether the location of pHGRs shows a random distribution along the length of the proteins or, alternatively, there is preference for any given regions such as the C- or N-terminus. The central positions of all pHGRs detected for any given organism were calculated and classified in ten different groups according to their relative location along their respective protein. The first group contained those pHGRs having their center in the N-terminal 10% of the protein sequence; the second group those with center in the second 10%, and so on. Figure 4A shows the frequency distribution of these ten groups for the eight fungi and indicates that there is no clear preference for any protein region, although slightly higher frequencies are observed for the N- and C-terminus, especially the latter, for almost all fungi examined. The clearer exception is *S. cerevisiae*, which seems to have a tendency to bear pHGRs in the central part of proteins. This tendency is confirmed by the fact that a similar study made with the set of data in which the *O*-glycosylation positions were randomized (Figure 4B) resulted in a completely different distribution, with pHGRs more homogeneously scattered along the length of proteins.

The location of pHGRs towards protein ends can be more clearly seen when only secretory enzymes are considered. This was studied by analyzing a specific set of proteins from *B. cinerea* predicted to have signal peptide and classified as enzymes active on polysaccharides in the CAZY database [16,17]. This list of proteins contains 177 members with signal peptide and at least one *O*-glycosylation site, as predicted by signalP and NetOGlyc, respectively. Among them, we found 72 enzymes displaying pHGRs (not shown). The distribution of these regions along the length of the respective proteins (Figure 4C) shows clearly a much more marked tendency to be located at the ends, especially at the C-terminus.

## Discussion

We have shown here that the most popular *in silico* tool to predict *O*-glycosylation, NetOGlyc, is able to predict *O*-glycosylation for fungal proteins, although with less accuracy than for mammalian proteins, and has a fairly good ability to predict regions with a high density of *O*-glycosylation, better that the mere search for Ser/Thr-rich regions. We have also shown that fungal secretory proteins are rich in regions with a high Ser/Thr content and are frequently predicted to have pHGRs of varying length, averaging 24 residues but going up to 821, that can be found anywhere along the proteins but have a slight tendency to be at either one of the two ends. The coincidence between Ser/Thr-rich regions and pHGRs was studied for a representative number (361) of *B. cinerea* proteins (not shown), and the results obtained are similar to those shown in Figure 1, 91% of residues within pHGRs also belonged to a Ser/Thr-rich region, while only 25% of residues inside a Ser/Thr rich region were also within an pHGR. Although the abundance of Thr, Ser, and Pro residues has been used before to search for mucin-type regions in mammalian proteins [10], these results and the comparison of predicted vs. experimental HGRs (Figure 1) clearly show that NetOGlyc is a much more restrictive and accurate way to predict hyper-*O*-glycosylation in fungi, mainly because it produces a lower level of false positives.

Among the proteins predicted to have pHGRs we have identified some fungal proteins with an extremely high level of *O*-glycosylation. The *B. cinerea* genome, for example, codes for 9 proteins with 737–1764 residues, and signal peptide for secretion, that are predicted to be *O*-glycosylated in more than 400 of their amino acids, as well as 11 additional smaller proteins, up to 300 amino acids, with more than 75% *O*-glycosylated residues (Additional file 2). Even considering that the actual number of *O*-glycosylation sites maybe 68% of these (see the overestimation rate calculated for NetOGlyc in the results section), this level of *O*-glycosylation does not seem compatible with the globular fold typical of enzymes or effector proteins, thus leading to the hypothesis that these proteins may be involved in maintaining the structure of the cell wall or the extracellular matrix. Most of them were predicted to have a GPI anchor at the C-terminus by at least one of the available prediction tools [18,19], while others were homologues to proteins classified as GPI anchored proteins in other fungi or to proteins experimentally proven to be in the cell wall. Curiously, a BLAST search revealed that 5 out of the 9 *B. cinerea* proteins with more than 400 predicted *O*-glycosylation sites have homologues only in the closely related fungus *S. sclerotiorum*, but not in any other organism, raising the question of whether they make any contribution to the lifestyle of these two highly successful, broad range, plant pathogens.

Some of these highly *O*-glycosylated proteins in *B. cinerea* display interesting similarities/motifs: Bofut4_P004110.1, a 670-aa protein predicted to be *O*-glycosylated in 75% of its residues, is similar (BLAST expect value = 4x10^-7^) to the *S. cerevisiae* protein Sed1p [20], a structural component of the cell wall. Bofut4_P104050.1, a 903-aa protein predicted to be *O*-glycosylated in 453 of them, is only present in *B. cinerea* and *S. sclerotiorum* and has two CFEM motifs that were proposed to be involved in virulence [21]. Bofut4_P131790.1, a 938-aa protein predicted to be *O*-glycosylated in 414 residues, is homologous to the *Metarhizium anisopliae* protein Mad1 mediating adhesion to insect cuticle, raising the question of a putative role in spore dispersion. However, most of these proteins, with more than 400 *O*-glycosylated residues or with more than 75% *O*-glycosylated residues, have no similarity to proteins of known function. It would be especially interesting to search, among those proteins highly *O*-glycosylated, of candidate virulence factors involved in adhesion to the host surfaces. The existence of these *O*-glycosylated adhesion proteins is predicted from the fact that *O*-glycosylation deficient mutants in fungal pathogens have been shown to be affected in adhesion to the host [5,6,22].

An *in silico* search in *U. maydis* for a special type of *O*-glycosylated proteins, those predicted to be glycosylated specifically by PMT4, allowed the identification of 64 putative PMT4 substrates [6]. Only 14 of these were included in our initial set of *U. maydis* proteins used in the search for pHGRs, since the rest did not show any signal peptide in the prediction carried out with SignalP. Interestingly, 13 of these 14 proteins were also predicted to be highly *O*-glycosylated in this study, in a region overlapping with the putative site serving as PMT4 substrate in all but in one case in which the pHGR and the PMT4 glycosylation site were adjacent. Bearing in mind that both the results reported in this study and those of Fernández-Álvarez *et al.*[6] are plain *in silico* predictions, the fact that they coincide to a great extent encourages using these predictions in the experimental search for highly *O*-glycosylated regions in proteins.

We have found experimentally some of the putatively hyper-*O*-glycosylated *B. cinerea* proteins in the early secretome. 26 of the 105 proteins identified in the early secretome [23] are predicted to have at least one pHGR (not shown). This group contains proteins with a diverse set of functions, but is enriched in proteins that seem to be involved in the metabolism of the cell wall or extracellular matrix, such as ß-1,3-glucanosyltransferase or ß-1,3-endoglucanase. The rest are lytic enzymes for various soluble substrates or proteins with unknown function. Intriguingly, with the only exception of one endopolygalacturonase, no plant cell wall degrading enzymes were found in the set. This leads to the speculation of a possible role for HGRs in maintaining proteins in the extracellular matrix. Proteins involved in turning soluble polymers into monomers, such as proteases or ribonucleases, could carry a better function if retained in the vicinity of the fungal cell, and bearing an hyper-O-glycosylated region could provide that property by integrating the proteins in the very prominent glucan sheath of *B. cinerea *[24,25].

Another possible role for pHGRs could be to confer a specific topological configuration to the proteins. Such seems to be the case, for example, of the cell-surface GPI-anchored adhesin Epa1p from *Candida glabrata *[26], which bears a Ser/Thr-rich region proposed to be kept in an extended rode-like conformation by *O*-glycosylation [26]. This Ser/Thr region serves to protrude the proteins’ main body away from the GPI-anchored C-terminus on the cell membrane.

Given the prevalence of pHGRs among fungal secretory proteins and the variety of properties they may confer to the proteins displaying them, it is not surprising that mutants affected in *O*-glycosylation show pleiotropic phenotypes [2], including reduced viability and virulence [5,6]. *O*-glycosylation may be, therefore, worth exploring as a new target in the fight against fungal pathogens.

## Conclusions

We have shown here that the web-based *O*-glycosylation prediction tool NetOGlyc can be used to identify regions in fungal secretory proteins with a high probability of being highly *O*-glycosylated. The analysis of the complete set of putatively-secretory proteins from eight fungi showed that 38-61% of them display Ser/Thr-rich regions, i.e. regions of at least 20 residues with a minimum Ser/Thr content of 40%, and that 18-31% of them contain pHGRs, i.e. regions of 20 or more residues of which at least 25% are predicted to be *O*-glycosylated. pHGRs were found anywhere along proteins but have a slight preference for the proteins ends, especially the C-terminus.

## Methods

### Prediction of *O*-glycosylation sites in secretory proteins

Protein sequences used in this study were obtained from publically available databases. The whole set of proteins coded by the genomes of *Magnaporthe grisea* (strain 70–15), *Sclerotinia sclerotiorum* (strain 1980), *Ustilago maydis* (strain 521), *Aspergillus nidulans* (strain FGSC A4), and *Neurospora crassa* (strain N15) were obtained from the Broad Institute [27]. Those of *Botrytis cinerea* (strain T4), *Trichoderma reesei* (strain QM6a), and *Saccharomyces cerevisiae* (strain S288C) were obtained respectively from URGI [28], JGI [29], and SGD [30].

The predicted protein sequences for each genome were downloaded and transferred to a Microsoft Excel 2010 spreadsheet with the aid of Fasta2tab [31]. All proteins were then tested for the presence of a signal peptide for secretion, using the standalone version of SignalP 3.0 [32]. SignalP 3.0 has a false positive rate of 15%. Those proteins which gave positive result in each genome, i.e. all proteins putatively entering the secretory pathway at the endoplasmic reticulum, were then run through the web-based *O*-glycosylation prediction tool NetOGlyc 3.1 [12]. Results from NetOGlyc were saved as a text file from within the web browser and fed to Microsoft Word 2010 to transform these into an appropriate table format that could be incorporated into a Microsoft Excel 2010 spreadsheet (Additional file 2). The sets of proteins with randomized *O*-glycosylation positions were generated from the latter with the aid of the Rand function in Microsoft Excel. Each randomized set contains the same proteins as the original one. i.e. all signalP-positive proteins in a given genome, and the number of predicted *O*-glycosylation sites in every individual protein is also the same. The difference is that the position along the protein of every individual site was chosen by the generation of an appropriate random number (according to the length of each individual protein), being careful not to assign two sites to the same residue.

### Detection of Ser/Thr-rich regions and pHGRs

**Figure 5:**
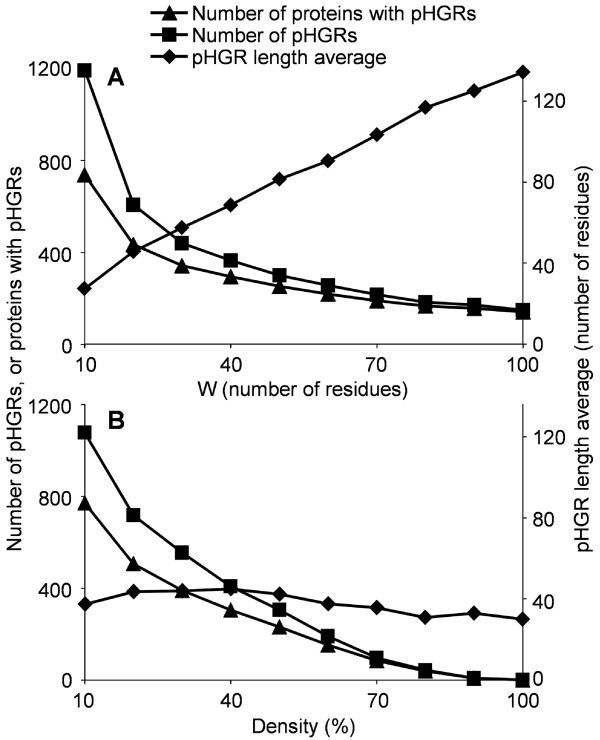
**Influence of the parameters *Window *(W) and *Density *(%G) on the detection of pHGRs.** The whole set of *B. cinerea* secretory proteins predicted by NetOGlyc to be *O*-glycosylated was scanned with the MS Excel macro XRR in search of pHGRs. **A**: results obtained with varying values of W and a fixed value for %G of 25%. **B**: results obtained with varying values of %G and a fixed value for W of 20.

To study the presence, in signalP-positive fungal proteins, of regions that are either rich in Ser/Thr or rich in predicted *O*-glycosylation, we first developed a simple algorithm that runs as a macro (named XRR) in a Microsoft Excel spreadsheet (Additional file 4), which was written with Microsoft Visual Basic for Applications. Although we have only used it to detect clusters of amino acids with a high content in Ser/Thr residues or a high number of predicted *O*-glycosylated residues, this macro can be used to search for the clustering of any protein modification or amino acid type along a sequence, i.e., to search for X-rich regions (where X stands for the kind of amino acid one is interested in). The algorithm just processes a list with the positions of the amino acids with the desired characteristics (X) and returns a list of protein regions rich in those amino acids (X-rich region). The version of the MS Excel macro included as supplementary material (Additional file 4) is able to analyze simultaneously up to 1500 proteins and is customized to search for hyper-*O*-glycosylated regions. Basically, the application scans the data searching for regions of a given length, called *Window* (W), having a *Density* (%G) of the desired amino acid characteristic above a minimum value. These regions can either be reported as independent X-rich regions, or can be combined into a single, longer region if several of them are found that overlap or are separated from one another by a number of amino acids which is less than the parameter *Separator* (S). The parameters W, %G, and S are set by the user. In any case, the beginning and end of X-rich regions are reported as the first and last amino acid with the desired properties in the group, so that for example, for W = 20 and %G = 25% (at least 5 positive hits in the window of 20 residues), X-rich regions as small as 5 amino acids could be reported. The results of the analysis are reported as a pdf file containing the data for all the X-rich regions encountered for each protein, both graphically and as a table, as well as several graphics with statistics for the whole set of proteins loaded. The influence of different values of the parameters W and %G on the detection of pHGRs was studied with the set of *B. cinerea* proteins predicted to have signal peptide (Figure 5). Lower values for both parameters, by making the analysis less stringent, resulted in a higher number of pHGRs, distributed in a broader set of proteins. Likewise, lower %G values tend to produce longer pHGRs, since the lower stringency permitted the pHGRs to be extended to neighboring regions displaying a not-so-high predicted sugar content. On the contrary, the average length of pHGRs increased with higher values of the parameter W, since this increase would eliminate the shorter ones as they would simply not be found.

### Control set of *O*-glycosylated proteins

In order to have a control set of proteins in the analysis of Ser/Thr-rich and predicted *O*-glycosylation-rich regions, we searched online databases and literature extensively in search of proteins from any fungi for which experimental evidence existed indicating *O*-glycosylation of at least one Ser or Thr residue, and only 30 proteins were found which were purified from its natural source (Additional file 1). The experimentally confirmed *O*-glycosylated positions in this set of 30 proteins were analyzed with the macro XRR to identify highly *O*-glycosylated regions, with the parameters set to result in low stringency (%G = 15, W = 20, S = 5). A total of 13 hyper-*O*-glycosylated regions were found in 12 of the 30 protein sequences (one protein displayed two separate regions), with an average length of 56 residues. Ser/Thr content in these regions resulted to be 38.5% ± 10.5, a value similar to that obtained for mucin domains in animal proteins [10].

## Abbreviations

HGR: Hyper-*O*-glycosylated Region; pHGR: Predicted Hyper-*O*-glycosylated Region; PMT: Protein Mannosyl Transferase.

## Competing interests

The authors declare that they have no competing interests.

## Authors’ contributions

NB and CG conceived the study. All authors participated in the design/evaluation of the algorithms used as well as the different analysis carried out with them. MG drafted the initial manuscript and all authors participated in the editing and approved its final version.

## Supplementary Material

Additional file 1 **Comparison of experimental*O*-glycosylation sites found in fungal proteins with those predicted by NetOGlyc 3.1 (http://www.cbs.dtu.dk/services/NetOGlyc/).** Click here for file

Additional file 2 **List of SignalP-positive proteins for the eight fungal genomes with the *O*-glycosylation sites predicted by NetOGlyc.** Click here for file

Additional file 3 **Results of the search for pHGRs (predicted Hyper-*O*-glycosylated Regions) in the SignalP-positive proteins coded by the eight fungal genomes.** Click here for file

Additional file 4 **Microsoft Excel spreadsheet with the macro XRR used in the search for Ser/Thr-rich regions and pHGRs (predicted Hyper-*O*-glycosylated Regions).** Click here for file
