# Supplementary material for: High abundance of Serine/Threonine-rich regions predicted to be hyper-O-glycosylated in the secretory proteins coded by eight fungal genomes
Source: BMC Microbiol. 2012 Sep 20;12:213. doi: 10.1186/1471-2180-12-213 (PMC3579731; doi:10.1186/1471-2180-12-213)

Table of contents:

Results for *Botrytis cinerea* T4 2
Results for *Aspergillus nidulans* 7
Results for *Magnaporthe grisea* 11
Results for *Neurospora crassa* 16
Results for *Saccharomyces cerevisiae* 21
Results for *Sclerotinia sclerotiorum* 24
Results for *Trichoderma reesei* 29
Results for *Ustilago maydis* 32

HGR_detection V1.1 Report.	*Botrytis cinerea*

1. Parameters used in HGR detection			
**Glycosylation Density (%)**	**Window**	**Limit**	**Separator**
25	20	5	5

2. Input protein set			
Number of proteins	1147	Largest protein	3211 aa
Protein length average	392,60	Smallest protein	34 aa

3. HGRs found in the protein set			
No. of proteins with HGRs	434	Number of HGRs	606
Average HGR length	45,59	Maximum HGR length	437
Average position of HGR centers (as % of protein lenght)	56,3	Minimum HGR length	7

4. Frequency distribution of the positions of HGR centers along the length of proteins

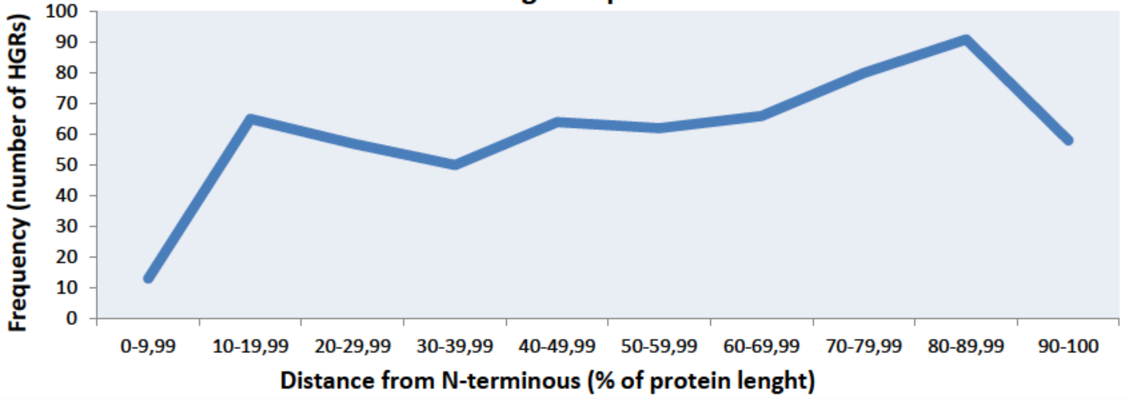

5. Frequency distribution of HGR lengths

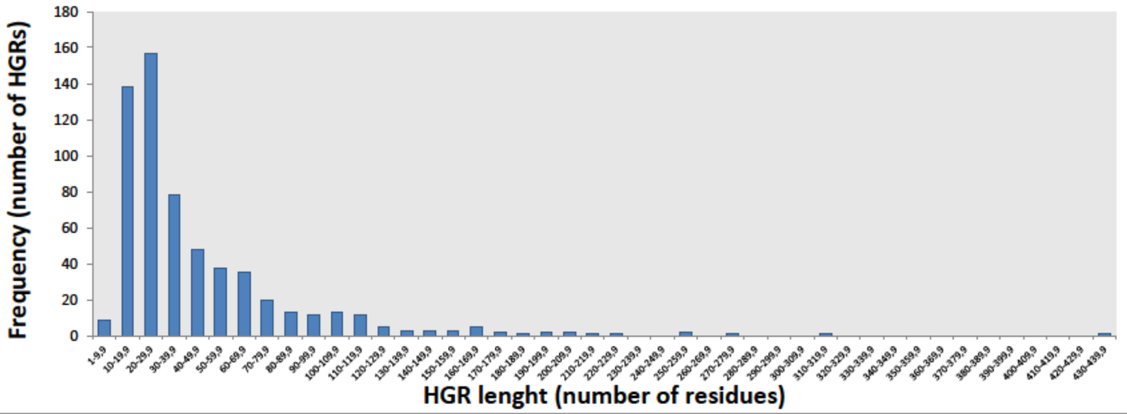

6. Alphabetical list of proteins with HGRs. Start and end positions of every HGR found.
(Protein name: start-end. start-end. start-end. …)

BofuT4_P000200.1 148-242.
BofuT4_P000760.1 376-391.
BofuT4_P001500.1 38-124. 171-186. 212-231.
BofuT4_P001910.1 315-342.
BofuT4_P002140.1 22-50.
BofuT4_P002420.1 200-217. 342-366.
BofuT4_P002470.1 29-48.
BofuT4_P002790.1 40-208.
BofuT4_P003860.1 398-416.
BofuT4_P003870.1 244-283.
BofuT4_P004000.1 53-71.
BofuT4_P004010.1 132-193. 234-255.
BofuT4_P004110.1 71-507. 597-641.
BofuT4_P004830.1 740-777.
BofuT4_P004950.1 71-90.
BofuT4_P005830.1 25-63.
BofuT4_P006210.1 97-119.
BofuT4_P006240.1 55-73. 153-220.
BofuT4_P006720.1 53-110. 154-226. 286-306.
BofuT4_P007160.1 135-168.
BofuT4_P008050.1 85-151.
BofuT4_P008060.1 225-250.
BofuT4_P008120.1 44-140.
BofuT4_P008250.1 208-243.
BofuT4_P009040.1 25-54. 160-235.
BofuT4_P009160.1 70-94. 137-154. 176-207. 227-353. 372-399. 470-492.
BofuT4_P009310.1 91-306.
BofuT4_P011390.1 166-239.
BofuT4_P012740.1 164-179.
BofuT4_P012770.1 157-190.
BofuT4_P013590.1 80-182. 235-268.
BofuT4_P013960.1 22-40.
BofuT4_P014300.1 451-480.
BofuT4_P014350.1 27-42.
BofuT4_P014500.1 20-94.
BofuT4_P014950.1 244-307.
BofuT4_P015800.1 33-46.
BofuT4_P015850.1 251-276. 361-390.
BofuT4_P016180.1 186-210.
BofuT4_P016340.1 51-96.
BofuT4_P016420.1 76-96.
BofuT4_P017380.1 287-297.
BofuT4_P018070.1 369-391.
BofuT4_P018380.1 455-539.
BofuT4_P018520.1 408-420.
BofuT4_P019100.1 117-141.
BofuT4_P019760.1 514-547.
BofuT4_P019940.1 179-203.
BofuT4_P020810.1 46-103.
BofuT4_P020980.1 197-212.
BofuT4_P021060.1 86-112. 154-258.
BofuT4_P021090.1 156-249. 306-420.
BofuT4_P021380.1 309-333.
BofuT4_P021460.1 65-86.
BofuT4_P023190.1 164-183.
BofuT4_P023240.1 70-98.
BofuT4_P023270.1 52-78.
BofuT4_P023520.1 291-325.
BofuT4_P023590.1 116-158.
BofuT4_P023980.1 237-272.
BofuT4_P024070.1 123-145. 198-232.
BofuT4_P025380.1 261-374.
BofuT4_P025430.1 251-315.
BofuT4_P026250.1 76-327. 436-473.
BofuT4_P027090.1 121-162. 183-378.
BofuT4_P027580.1 402-412.
BofuT4_P027850.1 33-172.
BofuT4_P029160.1 74-87.
BofuT4_P029480.1 448-476.
BofuT4_P029570.1 87-164.
BofuT4_P029600.1 199-238.
BofuT4_P029640.1 118-235.
BofuT4_P029790.1 40-194.
BofuT4_P029810.1 88-148. 273-335. 378-397. 438-462. 500-526.
BofuT4_P030090.1 326-347.
BofuT4_P030320.1 457-470.
BofuT4_P030590.1 89-120. 140-165. 232-255.
BofuT4_P031020.1 500-549. 627-683. 748-800.
BofuT4_P031060.1 199-405.
BofuT4_P031560.1 54-70. 119-140.
BofuT4_P031650.1 287-356.
BofuT4_P031660.1 238-282.
BofuT4_P031700.1 38-56.
BofuT4_P031830.1 229-254.
BofuT4_P032040.1 114-141.
BofuT4_P032460.1 31-54.
BofuT4_P032960.1 33-44.
BofuT4_P033270.1 33-47. 271-372.
BofuT4_P033320.1 308-422.
BofuT4_P033360.1 32-57.
BofuT4_P033390.1 292-356.
BofuT4_P034570.1 316-381.
BofuT4_P035060.1 505-520.
BofuT4_P035230.1 40-51.
BofuT4_P035550.1 700-718. 736-758.
BofuT4_P035820.1 20-31.
BofuT4_P035920.1 128-162.
BofuT4_P036610.1 62-314.
BofuT4_P036850.1 68-88.
BofuT4_P037990.1 277-298.
BofuT4_P038220.1 137-163.
BofuT4_P038300.1 68-130.
BofuT4_P038520.1 160-201.
BofuT4_P039280.1 367-399.
BofuT4_P039380.1 348-411.
BofuT4_P039570.1 127-143.
BofuT4_P039780.1 30-58. 87 105.
BofuT4_P039890.1 744-760.
BofuT4_P040100.1 20-42.
BofuT4_P040350.1 67-80.
BofuT4_P040360.1 73-87.
BofuT4_P040970.1 35-72.
BofuT4_P041440.1 68-137.
BofuT4_P041860.1 645-664. 679-703.
BofuT4_P042150.1 371-419.
BofuT4_P044540.1 92-152.
BofuT4_P044960.1 130-277.
BofuT4_P045000.1 19-32. 122-166.
BofuT4_P045480.1 752-762.
BofuT4_P045990.1 283-359.
BofuT4_P046230.1 151-174.
BofuT4_P047020.1 36-79.
BofuT4_P047770.1 280-293.
BofuT4_P047820.1 368-396. 435-485. 537-595.
BofuT4_P047970.1 26-87.
BofuT4_P048010.1 152-196.
BofuT4_P048140.1 29-52.
BofuT4_P048310.1 99-115.
BofuT4_P048790.1 339-357.
BofuT4_P048850.1 56-81. 511-522.
BofuT4_P049340.1 171-210. 437-455.
BofuT4_P049370.1 39-51.
BofuT4_P049790.1 339-350.
BofuT4_P050480.1 197-212.
BofuT4_P050550.1 112-179.
BofuT4_P050560.1 173-217.
BofuT4_P050650.1 357-384.
BofuT4_P050800.1 28-40. 80 97. 215-251. 267-283. 507-527. 561-618.
BofuT4_P051380.1 39-70.
BofuT4_P051420.1 52-65.
BofuT4_P051770.1 412-439.
BofuT4_P052010.1 416-448.
BofuT4_P052700.1 46-132.
BofuT4_P053390.1 98-172.
BofuT4_P054050.1 666-707.
BofuT4_P054440.1 19-39.
BofuT4_P054480.1 90-114.
BofuT4_P055250.1 528-552. 605-646. 717-754. 786-819.
BofuT4_P055490.1 93-115.
BofuT4_P056350.1 408-491.
BofuT4_P056370.1 270-330.
BofuT4_P056650.1 252-263. 367-382.
BofuT4_P057610.1 43-65. 235-271.
BofuT4_P057770.1 66-76.
BofuT4_P058210.1 534-562.
BofuT4_P059050.1 133-214. 434-454.
BofuT4_P059250.1 61-95.
BofuT4_P059260.1 62-77.
BofuT4_P059550.1 116-213. 243-273.
BofuT4_P059730.1 178-195.
BofuT4_P060340.1 30-77.
BofuT4_P060790.1 137-183.
BofuT4_P060820.1 336-360. 401-428.
BofuT4_P061290.1 32-65.
BofuT4_P061530.1 60-99.
BofuT4_P061550.1 45-69.
BofuT4_P062070.1 26-103.
BofuT4_P064000.1 35-52.
BofuT4_P064350.1 55-86.
BofuT4_P064370.1 266-312.
BofuT4_P064540.1 94-219.
BofuT4_P064930.1 157-177.
BofuT4_P065420.1 235-351.
BofuT4_P065630.1 33-50.
BofuT4_P065730.1 444-462. 679-705. 753-785. 959-979. 1060-1127. 1197-1211.
BofuT4_P066040.1 61-78.
BofuT4_P066110.1 24-35.
BofuT4_P066750.1 49-126. 490-509. 520-563. 899-939. 989-1043. 1065-1171.
BofuT4_P066930.1 405-429.
BofuT4_P067280.1 259-301.
BofuT4_P068540.1 83-115.
BofuT4_P069360.1 108-132.
BofuT4_P070600.1 27-107.
BofuT4_P070670.1 51-68.
BofuT4_P071490.1 31-59. 99-119. 180-228. 291-299. 327-357.
BofuT4_P071570.1 651-668.
BofuT4_P071810.1 29-157. 203-297.
BofuT4_P072500.1 477-514.
BofuT4_P072610.1 22-38.
BofuT4_P072900.1 332-379.
BofuT4_P072970.1 222-247.
BofuT4_P073050.1 150-195.
BofuT4_P073150.1 304-325.
BofuT4_P073830.1 166-182.
BofuT4_P074080.1 317-335.
BofuT4_P075410.1 217-266.
BofuT4_P075540.1 100-127. 192-268.
BofuT4_P075860.1 31-129.
BofuT4_P076030.1 58-108. 118-181.
BofuT4_P076050.1 82-157. 166-213.
BofuT4_P076320.1 607-635.
BofuT4_P077180.1 24-35. 109-130.
BofuT4_P077720.1 68-90.
BofuT4_P078510.1 133-155.
BofuT4_P079250.1 120-202.
BofuT4_P079300.1 57-80.
BofuT4_P079560.1 132-142.
BofuT4_P079660.1 122-178.
BofuT4_P079690.1 32-46.
BofuT4_P081140.1 38-106. 344-357.
BofuT4_P082390.1 213-235.
BofuT4_P082560.1 37-61.
BofuT4_P082690.1 683-700. 814-828.
BofuT4_P082800.1 200-234. 397-413. 435-468.
BofuT4_P083010.1 38-62.
BofuT4_P083150.1 29-168.
BofuT4_P083220.1 47-65. 194-203.
BofuT4_P083380.1 110-137.
BofuT4_P083790.1 109-271.
BofuT4_P083940.1 24-36.
BofuT4_P084350.1 457-467.
BofuT4_P084540.1 169-200.
BofuT4_P084590.1 42-101. 125-350.
BofuT4_P084900.1 228-237.
BofuT4_P085120.1 112-156.
BofuT4_P086450.1 21-39.
BofuT4_P086630.1 56-135.
BofuT4_P088110.1 508-523.
BofuT4_P088780.1 32-105. 287-304.
BofuT4_P089480.1 261-325. 364-390.
BofuT4_P089770.1 564-584.
BofuT4_P090110.1 40-86.
BofuT4_P090590.1 156-200. 354-384.
BofuT4_P091080.1 25-48.
BofuT4_P091190.1 27-38.
BofuT4_P091600.1 330-354.
BofuT4_P092230.1 143-210. 303-406.
BofuT4_P092250.1 39-99.
BofuT4_P092290.1 39-52.
BofuT4_P092720.1 191-209.
BofuT4_P093290.1 397-427.
BofuT4_P093720.1 96-366. 416-445.
BofuT4_P094060.1 332-371.
BofuT4_P094200.1 137-161.
BofuT4_P094270.1 470-485. 569-586.
BofuT4_P094770.1 390-419.
BofuT4_P095270.1 515-571.
BofuT4_P095480.1 26-54.
BofuT4_P095690.1 26-47.
BofuT4_P095740.1 214-245.
BofuT4_P096290.1 65-239.
BofuT4_P098480.1 80-119.
BofuT4_P098810.1 221-235.
BofuT4_P099150.1 259-576. 678-701.
BofuT4_P099240.1 20-39.
BofuT4_P100330.1 303-335. 349-436.
BofuT4_P100630.1 31-44.
BofuT4_P100680.1 39-142.
BofuT4_P101030.1 189-223. 409-430.
BofuT4_P101670.1 53-72.
BofuT4_P101730.1 120-139.
BofuT4_P102430.1 477-503.
BofuT4_P102910.1 180-344.
BofuT4_P103080.1 291-311.
BofuT4_P103160.1 245-305.
BofuT4_P103280.1 226-311.
BofuT4_P103460.1 21-38.
BofuT4_P104050.1 118-171. 306-495. 520-684.
BofuT4_P104090.1 95-118.
BofuT4_P104230.1 330-345.
BofuT4_P104330.1 101-129.
BofuT4_P105860.1 75-87.
BofuT4_P107070.1 486-547. 637-675.
BofuT4_P107230.1 35-57.
BofuT4_P107570.1 83-112.
BofuT4_P107750.1 118-159. 189-224.
BofuT4_P107840.1 19-33.
BofuT4_P108190.1 133-197. 283-339.
BofuT4_P108800.1 32-52.
BofuT4_P109230.1 241-294.
BofuT4_P109330.1 249-269.
BofuT4_P109530.1 280-287.
BofuT4_P109590.1 46-75. 108-218.
BofuT4_P109780.1 198-212. 304-320. 507-520.
BofuT4_P109890.1 75-121. 143-168.
BofuT4_P109960.1 141-199.
BofuT4_P110550.1 300-332. 420-435. 883-933. 964-1027. 1073-1260. 1327-
BofuT4_P110590.1 100-135.
BofuT4_P110840.1 211-230.
BofuT4_P110970.1 166-172.
BofuT4_P111650.1 30-43.
BofuT4_P111880.1 315-392.
BofuT4_P111940.1 233-346. 369-523.
BofuT4_P114350.1 190-213.
BofuT4_P114590.1 61-113.
BofuT4_P114670.1 147-215.
BofuT4_P114690.1 40-57. 198-232.
BofuT4_P114910.1 473-503.
BofuT4_P115020.1 71-107.
BofuT4_P115530.1 359-375.
BofuT4_P116300.1 64-133. 171-233. 254-304. 339-357. 453-544. 633-685.
BofuT4_P116810.1 295-326.

BofuT4_P116860.1	753-768.
BofuT4_P118690.1	457-541.
BofuT4_P118950.1	28-42.
BofuT4_P119060.1	25-40.
BofuT4_P119250.1	53-78. 145-162.
BofuT4_P119450.1	106-160. 301-321. 356-373. 430-457.
BofuT4_P120190.1	59-78. 161-201. 214-243.
BofuT4_P120520.1	19-35.
BofuT4_P120550.1	244-273. 299-341.
BofuT4_P120590.1	48-79.
BofuT4_P121910.1	490-514.
BofuT4_P121960.1	359-397. 436-462.
BofuT4_P122570.1	28-48.
BofuT4_P122630.1	24-44.
BofuT4_P122770.1	61-90.
BofuT4_P123190.1	110-135.
BofuT4_P124300.1	458-516.
BofuT4_P124770.1	430-466.
BofuT4_P124950.1	219-234. 543-562.
BofuT4_P125080.1	108-118.
BofuT4_P125090.1	451-476.
BofuT4_P125220.1	148-215.
BofuT4_P125570.1	296-319.
BofuT4_P126460.1	59-82. 126-156. 200-224.
BofuT4_P128390.1	276-301.
BofuT4_P129080.1	87-192.
BofuT4_P129150.1	88-108.
BofuT4_P129340.1	336-351. 518-568. 669-686.
BofuT4_P130330.1	130-196.
BofuT4_P131080.1	55-160.
BofuT4_P131160.1	601-620.
BofuT4_P131670.1	226-256. 303-329.
BofuT4_P131790.1	223-244. 306-328. 403-496. 570-702. 796-854. 881-907.
BofuT4_P132140.1	123-234.
BofuT4_P132260.1	79-135. 264-284. 373-424.
BofuT4_P132940.1	387-409.
BofuT4_P133000.1	58-66.
BofuT4_P133340.1	107-128. 163-292. 327-355. 398-528. 546-576. 603-656. 711-775. 954-983. 1075-1141.
BofuT4_P133520.1	260-274.
BofuT4_P133860.1	84-114. 220-238.
BofuT4_P134190.1	78-92. 131-160.
BofuT4_P134240.1	189-294.
BofuT4_P134700.1	211-219. 764-796.
BofuT4_P135520.1	530-559.
BofuT4_P135810.1	153-204.
BofuT4_P136240.1	103-131.
BofuT4_P136250.1	192-269.
BofuT4_P136540.1	20-45. 154-210.
BofuT4_P136550.1	336-407.
BofuT4_P136730.1	467-496.
BofuT4_P137590.1	306-360.
BofuT4_P137730.1	51-93. 302-357.
BofuT4_P138260.1	162-186.
BofuT4_P138400.1	328-344.
BofuT4_P138500.1	182-194.
BofuT4_P138980.1	225-342.
BofuT4_P139360.1	168-182. 291-305.
BofuT4_P139490.1	88-101. 180-194.
BofuT4_P140270.1	464-480. 517-591.
BofuT4_P140830.1	26-64.
BofuT4_P141000.1	148-187.
BofuT4_P141150.1	149-166.
BofuT4_P141390.1	261-297.
BofuT4_P141400.1	41-203.
BofuT4_P141410.1	824-838.
BofuT4_P141890.1	192-295.
BofuT4_P142980.1	95-201.
BofuT4_P144440.1	75-191.
BofuT4_P144530.1	406-424.
BofuT4_P145020.1	446-555.
BofuT4_P145510.1	135-169.
BofuT4_P145730.1	195-211.
BofuT4_P145970.1	451-494.
BofuT4_P146410.1	132-143. 166-249.
BofuT4_P146870.1	378-474. 681-747. 808-857. 907-939.
BofuT4_P147300.1	73-85.
BofuT4_P147740.1	78-115.
BofuT4_P149470.1	25-43.
BofuT4_P149530.1	108-215.
BofuT4_P149760.1	1191-1243.
BofuT4_P150000.1	76-90. 139-237.
BofuT4_P150210.1	74-81.
BofuT4_P150620.1	223-242.
BofuT4_P150760.1	188-212. 312-363.
BofuT4_P151390.1	344-379.
BofuT4_P151520.1	179-242.
BofuT4_P152070.1	45-73.
BofuT4_P152630.1	309-323.
BofuT4_P152650.1	203-246. 534-547.
BofuT4_P152680.1	132-185.
BofuT4_P154120.1	139-188.
BofuT4_P154700.1	680-720.
BofuT4_P154780.1	69-111.
BofuT4_P154790.1	117-169.
BofuT4_P154810.1	437-457.
BofuT4_P155200.1	395-414.
BofuT4_P155440.1	301-313. 441-464. 511-534. 556-573.
BofuT4_P155600.1	139-148.
BofuT4_P156020.1	39-63.
BofuT4_P156400.1	389-404.
BofuT4_P156410.1	66-94. 164-183.
BofuT4_P156750.1	331-419. 471-499.
BofuT4_P156900.1	20-39.
BofuT4_P157050.1	23-70.
BofuT4_P157390.1	136-260.
BofuT4_P160670.1	43-57.
BofuT4_P160720.1	234-338.
BofuT4_P161220.1	23-97.
BofuT4_P161460.1	212-339.
BofuT4_P162440.1	31-54. 94 121. 146-166.
BofuT4_P162620.1	153-192.
BofuT4_P162630.1	51-204. 248-268. 308-402.
BofuT4_P163130.1	128-149. 156-223. 317-519. 541-566. 667-693. 769-814. 836-951. 995-1011. 1034-1124.
BofuT4_P163190.1	36-54. 145-162.
BofuT4_P163280.1	247-253.
BofuT4_P163510.1	472-509. 550-595.
BofuT4_uP005500.1	26-48.
BofuT4_uP025980.1	22-50.
BofuT4_uP037940.1	47-82.
BofuT4_uP043500.1	80-90.
BofuT4_uP050030.1	58-68.
BofuT4_uP097170.1	40-48.
BofuT4_uP123850.1	40-55.
BofuT4_uP127440.1	56-65.
BofuT4_uP137880.1	17-35.
BofuT4_uP142990.1	35-79.
BofuT4_uP143280.1	27-56.
BofuT4_uP144210.1	75-97.
BofuT4_uP146480.1	46-89.
BofuT4_uP147890.1	60-67.
BofuT4_uP156490.1	27-62.
BofuT4_uP157720.1	31-49.

7. Graphical representation of all HGRs found in the protein set

Proteins are represented as boxes of lenght proportional to protein length. Black regions indicate HGRs

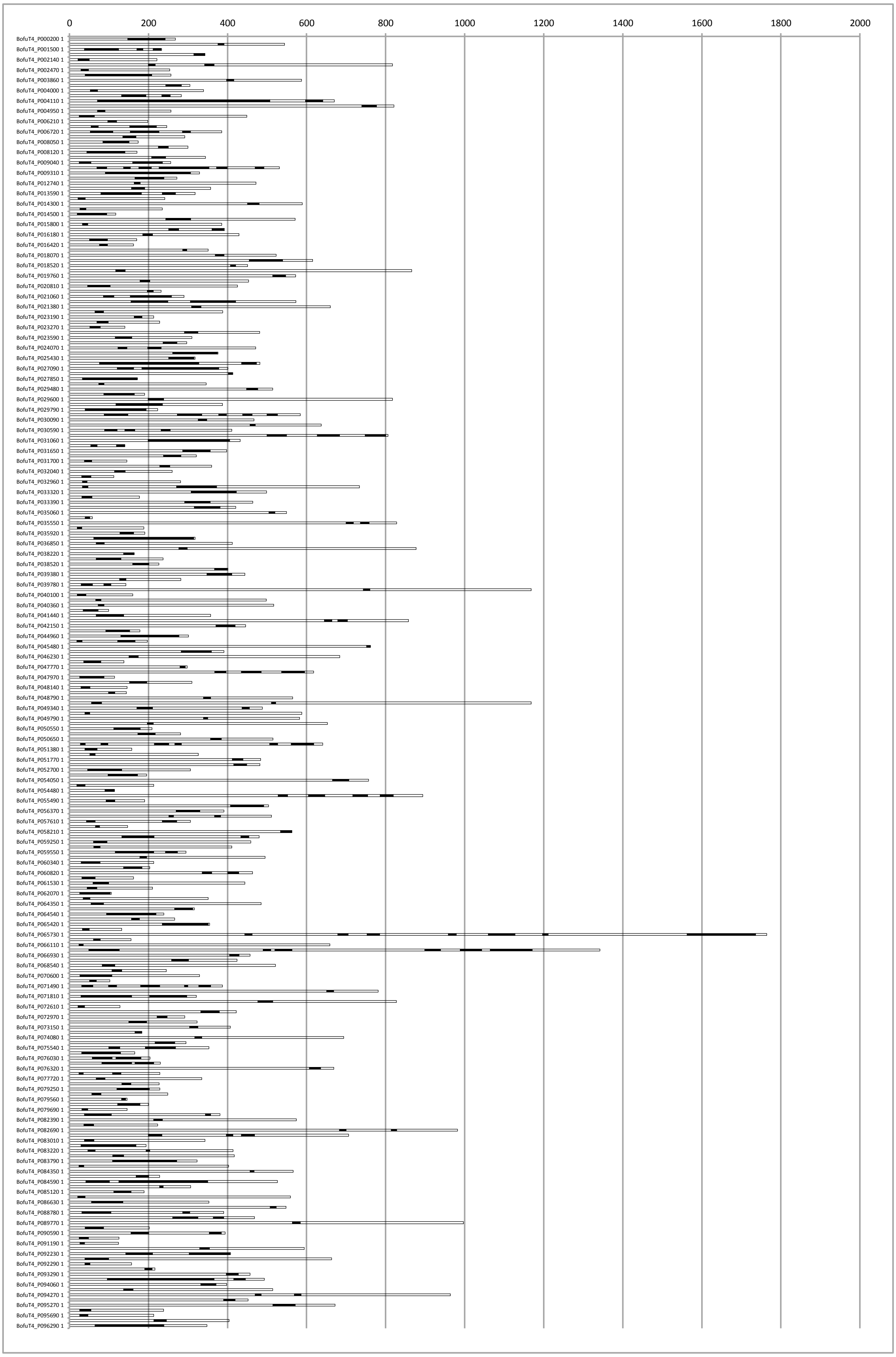

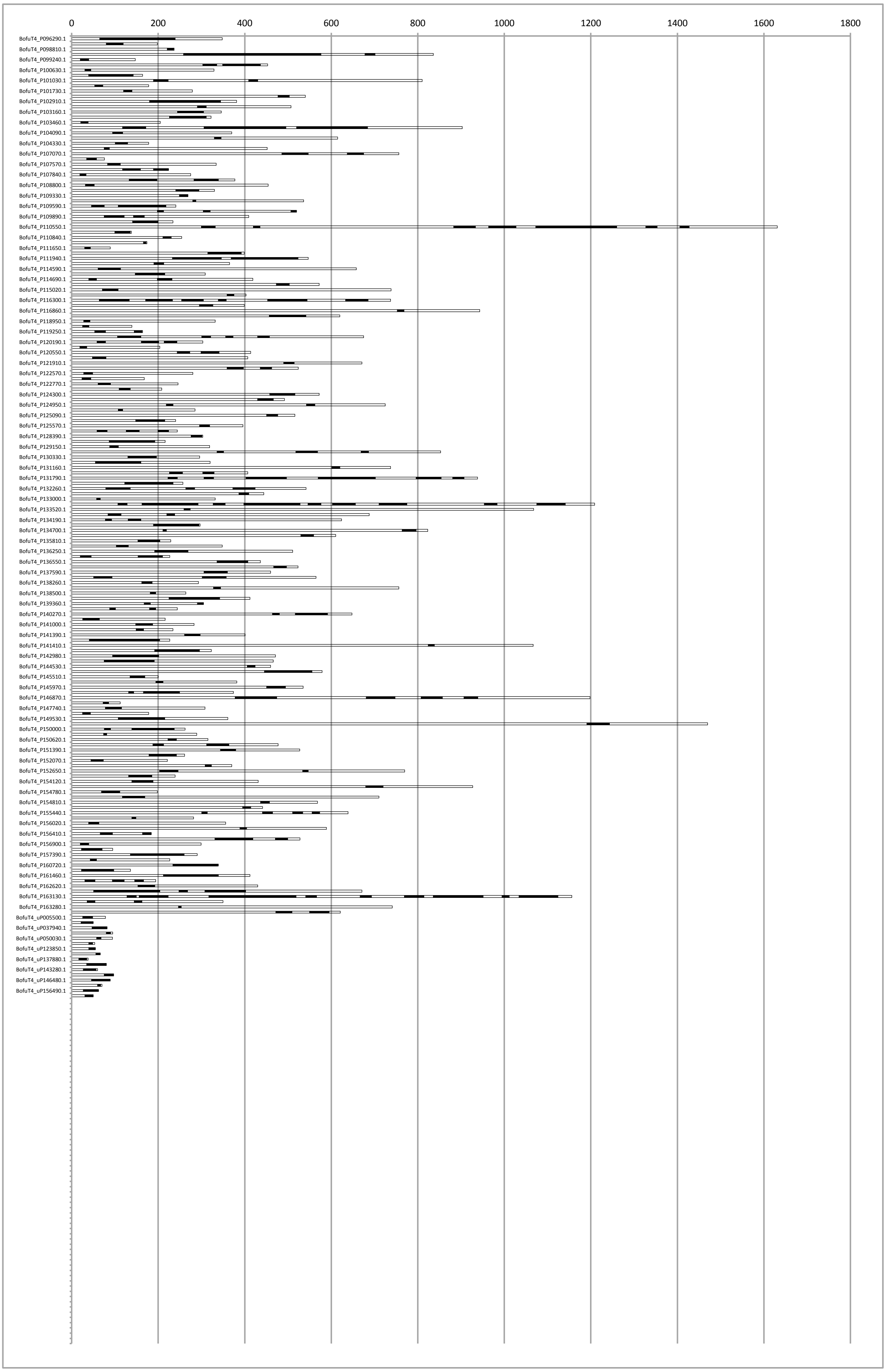
0
200
400
600
800
1000
1200
1400
1600
1800
BofuT4_P096290.1
BofuT4_P098810.1
BofuT4_P099240.1
BofuT4_P100630.1
BofuT4_P101030.1
BofuT4_P101730.1
BofuT4_P102910.1
BofuT4_P103160.1
BofuT4_P103460.1
BofuT4_P104090.1
BofuT4_P104330.1
BofuT4_P107070.1
BofuT4_P107570.1
BofuT4_P107840.1
BofuT4_P108800.1
BofuT4_P109330.1
BofuT4_P109590.1
BofuT4_P109890.1
BofuT4_P110550.1
BofuT4_P110840.1
BofuT4_P111650.1
BofuT4_P111940.1
BofuT4_P114590.1
BofuT4_P114690.1
BofuT4_P115020.1
BofuT4_P116300.1
BofuT4_P116860.1
BofuT4_P118950.1
BofuT4_P119250.1
BofuT4_P120190.1
BofuT4_P120550.1
BofuT4_P121910.1
BofuT4_P122570.1
BofuT4_P122770.1
BofuT4_P124300.1
BofuT4_P124950.1
BofuT4_P125090.1
BofuT4_P125570.1
BofuT4_P128390.1
BofuT4_P129150.1
BofuT4_P130330.1
BofuT4_P131160.1
BofuT4_P131790.1
BofuT4_P132260.1
BofuT4_P133000.1
BofuT4_P133520.1
BofuT4_P134190.1
BofuT4_P134700.1
BofuT4_P135810.1
BofuT4_P136250.1
BofuT4_P136550.1
BofuT4_P137590.1
BofuT4_P138260.1
BofuT4_P138500.1
BofuT4_P139360.1
BofuT4_P140270.1
BofuT4_P141000.1
BofuT4_P141390.1
BofuT4_P141410.1
BofuT4_P142980.1
BofuT4_P144530.1
BofuT4_P145510.1
BofuT4_P145970.1
BofuT4_P146870.1
BofuT4_P147740.1
BofuT4_P149530.1
BofuT4_P150000.1
BofuT4_P150620.1
BofuT4_P151390.1
BofuT4_P152070.1
BofuT4_P152650.1
BofuT4_P154120.1
BofuT4_P154780.1
BofuT4_P154810.1
BofuT4_P155440.1
BofuT4_P156020.1
BofuT4_P156410.1
BofuT4_P156900.1
BofuT4_P157390.1
BofuT4_P160720.1
BofuT4_P161460.1
BofuT4_P162620.1
BofuT4_P163130.1
BofuT4_P163280.1
BofuT4_uP005500.1
BofuT4_uP037940.1
BofuT4_uP050030.1
BofuT4_uP123850.1
BofuT4_uP137880.1
BofuT4_uP143280.1
BofuT4_uP146480.1
BofuT4_uP156490.1

HGR_detection V1.1 Report.	*Aspergillus nidulans*

1. Parameters used in HGR detection			
**Glycosylation Density (%)**	**Window**	**Limit**	**Separator**
25	20	5	5

2. Input protein set			
Number of proteins	932	Largest protein	3187 aa
Protein length average	460,89	Smallest protein	59 aa

3. HGRs found in the protein set			
No. of proteins with HGRs	269	Number of HGRs	345
Average HGR length	45,85	Maximum HGR length	507
Average position of HGR centers (as % of protein lenght)	55,4	Minimum HGR length	6

4. Frequency distribution of the positions of HGR centers along the length of proteins

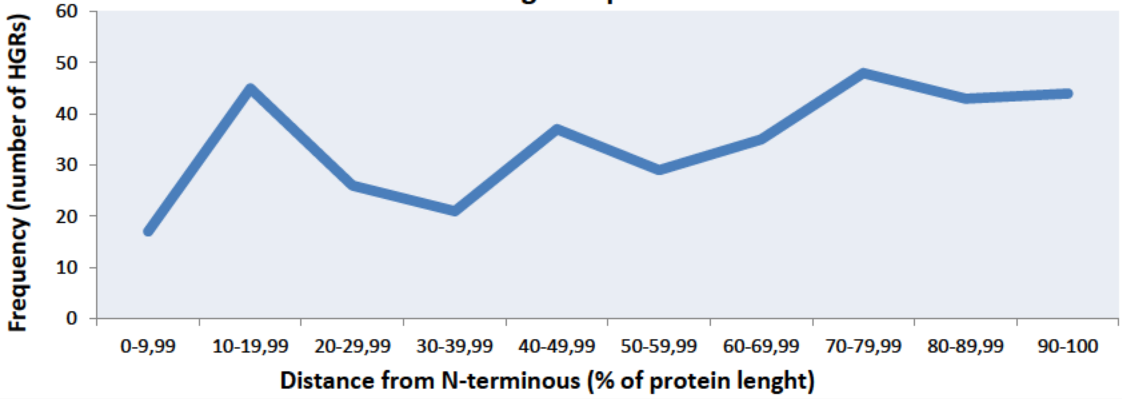

5. Frequency distribution of HGR lengths

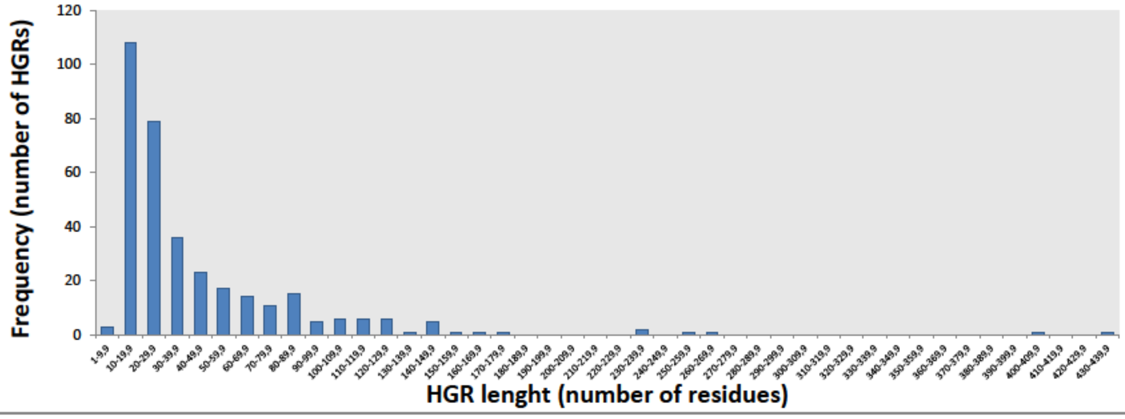

6. Alphabetical list of proteins with HGRs. Start and end positions of every HGR found. (Protein name: start-end. start-end. start-end. …)

AN D_00014T0 110-125.
AN D_00248T0 268-318.
AN D 00362T0 60-76.
AN D 00403T0 423-438.
AN D 00452T0 223-239.
AN D_00457T0 23-52.
AN D 00463T0 431-447.
AN D_00472T0 108-164.
AN D 00494T0 466-489.
AN D 00502T0 52-63.
AN D 00511T0 82-139.
AN D 00543T0 108-124. 189-209. 275-291. 359-376. 440-454.
AN D_00563T0 51-68.
AN D_00592T0 534-545.
AN D 00602T0 228-290.
AN D 00726T0 323-385. 414-426.
AN D 00778T0 200-282.
AN D 00779T0 464-534.
AN D_00803T0 486-495.
AN D_00810T0 918-939.
AN D_00822T0 702-713.
AN D 00847T0 833-845.
AN D 00933T0 268-378.
AN D 01027T0 258-273.
AN D 01041T0 255-395.
AN D_01058T0 30-145.
AN D_01160T0 27-140.
AN D 01218T0 361-404.
AN D 01230T0 43-74.
AN D 01273T0 27-37.
AN D_01286T0 441-469.
AN D 01330T0 86-147.
AN D_01338T0 23-34. 103-143.
AN D 01426T0 532-550.
AN D 01442T0 200-240.
AN D 01449T0 112-199.
AN D 01488T0 593-607.
AN D_01490T0 131-211.
AN D_01493T0 136-160.
AN D 01515T0 145-186.
AN D 01551T0 54-288.
AN D 01559T0 238-272.
AN D 01561T0 100-184.
AN D 01602T0 235-316.
AN D_01627T0 78-104. 126-174.
AN D_01716T0 96-116. 150-171. 258-309.
AN D_01753T0 32-68.
AN D 01803T0 31-63. 153-168.
AN D 01813T0 58-74.
AN D 01833T0 86-101.
AN D_01870T0 235-251.
AN D_01941T0 125-144.
AN D_02017T0 560-579.
AN D 02237T0 556-568.
AN D 02305T0 326-337.
AN D 02312T0 93-142.
AN D 02338T0 110-144. 195-235.
AN D_02385T0 342-442. 479-602.
AN D_02445T0 92-166.
AN D 02582T0 284-380.
AN D 02659T0 112-259. 296-465. 690-807.
AN D 02690T0 29-34.
AN D 02702T0 103-204.
AN D_02752T0 34-46.
AN D_02828T0 614-627.
AN D 02851T0 115-135.
AN D 02922T0 124-164.
AN D 02928T0 447-697.
AN D 02954T0 32-131.
AN D_03032T0 42-139. 147-183. 204-229. 319-372.
AN D_03042T0 102-153. 210-259.
AN D_03086T0 111-197. 589-599.
AN D 03115T0 450-488.
AN D 03127T0 257-288.
AN D 03141T0 193-594. 670-800.
AN D_03215T0 132-158.
AN D_03258T0 133-186. 215-239.
AN D_03259T0 46-82.
AN D 03285T0 29-43.
AN D 03310T0 22-37. 116-200.
AN D 03321T0 440-456.
AN D 03358T0 23-48.
AN D_03377T0 87-104.
AN D_03402T0 497-518.
AN D 03404T0 125-171.
AN D 03520T0 391-406. 501-515.
AN D 03529T0 144-178.
AN D 03583T0 650-699.
AN D 03592T0 546-559.
AN D_03606T0 217-239.
AN D_03631T0 94-168. 219-296.
AN D 03670T0 84-116. 157-190.
AN D 03671T0 96-129.
AN D 03692T0 372-401.
AN D 03727T0 50-62.
AN D_03798T0 117-172. 198-224.
AN D_03818T0 297-347.
AN D 03914T0 287-321.
AN D 03959T0 376-408.
AN D 04047T0 392-435.
AN D 04049T0 136-211.
AN D_04055T0 21-36.
AN D_04121T0 177-258. 468-487.
AN D 04190T0 33-161.
AN D 04260T0 126-162.
AN D 04354T0 109-170.
AN D 04378T0 336-347.
AN D_04379T0 49-208.
AN D_04381T0 88-153.
AN D_04389T0 68-88. 139-159.
AN D 04390T0 344-368.
AN D 04422T0 429-473.
AN D 04438T0 46-481. 519-551.
AN D 04480T0 49-190. 385-405. 573-601.
AN D_04565T0 76-96.
AN D_04575T0 38-116.
AN D 04637T0 605-627.
AN D 04674T0 116-181.
AN D 04716T0 47-59.
AN D 04812T0 51-173.
AN D_04814T0 392-400.
AN D_04845T0 256-333.
AN D_04846T0 149-203.
AN D 04879T0 65-184.
AN D 04882T0 384-464.
AN D 04897T0 27-169.
AN D_04950T0 47-108.
AN D_04970T0 23-97.
AN D 05061T0 746-793.
AN D 05076T0 212-239. 341-372.
AN D 05125T0 73-91.
AN D 05149T0 139-228. 373-399.
AN D_05197T0 1002-1047.
AN D_05282T0 36-69.
AN D_05290T0 83-151.
AN D 05328T0 374-395.
AN D 05357T0 94-130. 185-284.
AN D 05391T0 115-128.
AN D 05397T0 74-122.
AN D 05398T0 350-366.
AN D_05463T0 256-280. 368-381.
AN D_05660T0 126-158.
AN D 05683T0 45-72. 244-258.
AN D 05762T0 104-174.
AN D 05818T0 85-119.
AN D 05834T0 32-141.
AN D_05919T0 474-576.
AN D_06049T0 325-366.
AN D_06134T0 170-202. 240-308. 594-619. 1376-1399.
AN D 06321T0 350-369.
AN D 06465T0 211-226.

ANID_06471T0 116-177.
ANID_06535T0 19-35.
ANID 06553T0 208-226. 253-269.
ANID 06656T0 85-172.
ANID 06664T0 101-121. 194-215. 281-293. 361-388.
ANID_06697T0 81-136.
ANID 06873T0 150-189.
ANID_06927T0 123-202.
ANID 06937T0 208-231. 259-341.
ANID 06965T0 81-123. 186-205.
ANID 07041T0 120-145. 175-204. 255-371. 425-455. 467-566.
ANID 07053T0 120-273.
ANID_07101T0 46-126.
ANID_07166T0 49-173.
ANID 07191T0 80-102. 202-254. 273-292.
ANID 07322T0 180-209.
ANID 07323T0 98-187.
ANID 07366T0 284-344.
ANID_07401T0 323-347.
ANID_07402T0 471-532. 617-632.
ANID_07454T0 69-81.
ANID 07457T0 29-50. 345 361.
ANID 07515T0 79-94.
ANID 07534T0 53-78.
ANID 07535T0 55-79.
ANID_07541T0 217-246.
ANID_07548T0 52-64.
ANID 07551T0 34-264.
ANID 07657T0 392-409.
ANID 07672T0 35-61.
ANID_07673T0 202-222.
ANID 07716T0 121-183.
ANID_07735T0 45-171.
ANID 07754T0 207-223.
ANID 07758T0 81-98.
ANID 07778T0 24-40. 167 183.
ANID 07847T0 75-118. 185-195. 264-286.
ANID_07857T0 140-150.
ANID_07858T0 391-404.
ANID 07891T0 55-70. 246 329.
ANID 07897T0 140-152.
ANID 07946T0 106-123.
ANID 07948T0 329-344.
ANID 07950T0 325-340. 375-441.
ANID_07975T0 70-97.
ANID_08011T0 24-36.
ANID_08154T0 41-84.
ANID 08167T0 367-378. 405-431.
ANID 08175T0 254-339.
ANID 08196T0 32-50. 209 218.
ANID_08241T0 347-853. 917-933.
ANID_08319T0 275-323.
ANID_08382T0 20-43.
ANID 08421T0 392-407.
ANID 08461T0 52-130.
ANID 08466T0 137-158. 232-253. 313-326.
ANID 08484T0 187-450.
ANID_08509T0 126-146. 303-315.
ANID_08512T0 357-374.
ANID 08602T0 180-204.
ANID 08609T0 63-95. 156 169.
ANID 08624T0 143-176.
ANID 08660T0 534-554.
ANID_08675T0 501-510.
ANID_08730T0 201-236.
ANID 08775T0 24-78. 170 188.
ANID 08822T0 3-26.
ANID 08830T0 2-18. 81-124.
ANID 08902T0 92-106.
ANID_08913T0 20-166.
ANID_08957T0 1390-1421.
ANID_08963T0 64-88.
ANID 08981T0 139-146.
ANID 08994T0 10-52.
ANID 09042T0 483-559.
ANID_09056T0 254-275.
ANID_09090T0 21-41.
ANID_09201T0 51-65.
ANID 09263T0 116-211.
ANID 09351T0 2-15.
ANID 09406T0 467-492.
ANID 09433T0 3-16.
ANID_09481T0 171-197.
ANID_09482T0 26-46.
ANID 09517T0 2-48. 79-104. 477-494.
ANID 10019T0 104-123. 141-168.
ANID 10064T0 8-18. 198-215.
ANID 10090T0 214-228. 317-334.
ANID 10105T0 2-23. 968-984.
ANID_10181T0 415-442.
ANID_10211T0 393-404.
ANID 10229T0 25-40.
ANID 10283T0 2-12.
ANID 10488T0 27-42.
ANID 10547T0 22-31.
ANID_10575T0 30-87.
ANID_10805T0 563-580.
ANID 11018T0 59-79.
ANID 11046T0 88-103.
ANID 11048T0 176-200.
ANID 11143T0 485-509.
ANID_11152T0 457-496.
ANID_11159T0 386-404.
ANID 11245T0 59-82.
ANID 11378T0 43-69.
ANID 11740T0 184-215.
ANID 11761T0 193-220.
ANID_11769T0 107-123.
ANID_11955T0 36-55.
ANID_11957T0 37-59.
ANID 11979T0 274-306.
ANID 11982T0 41-57.
ANID 12161T0 330-359.
ANID 12207T0 29-50.
ANID_12227T0 82-91.
ANID_12299T0 144-165. 278-292.
ANID 12337T0 404-414. 736-749. 774-790.
ANID 12429T0 557-570.

7. Graphical representation of all HGRs found in the protein set

Proteins are represented as boxes of lenght proportional to protein length. Black regions indicate HGRs

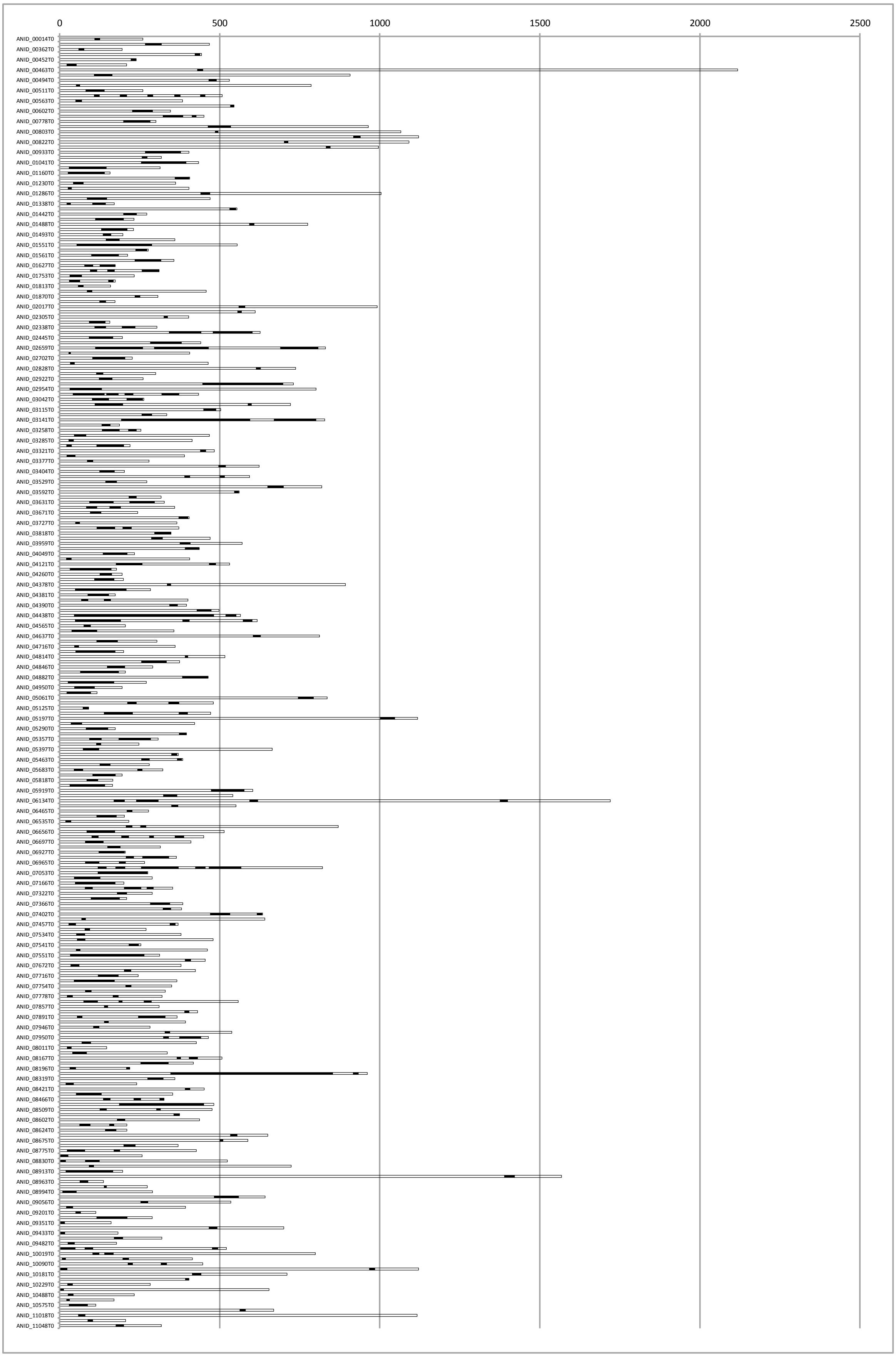

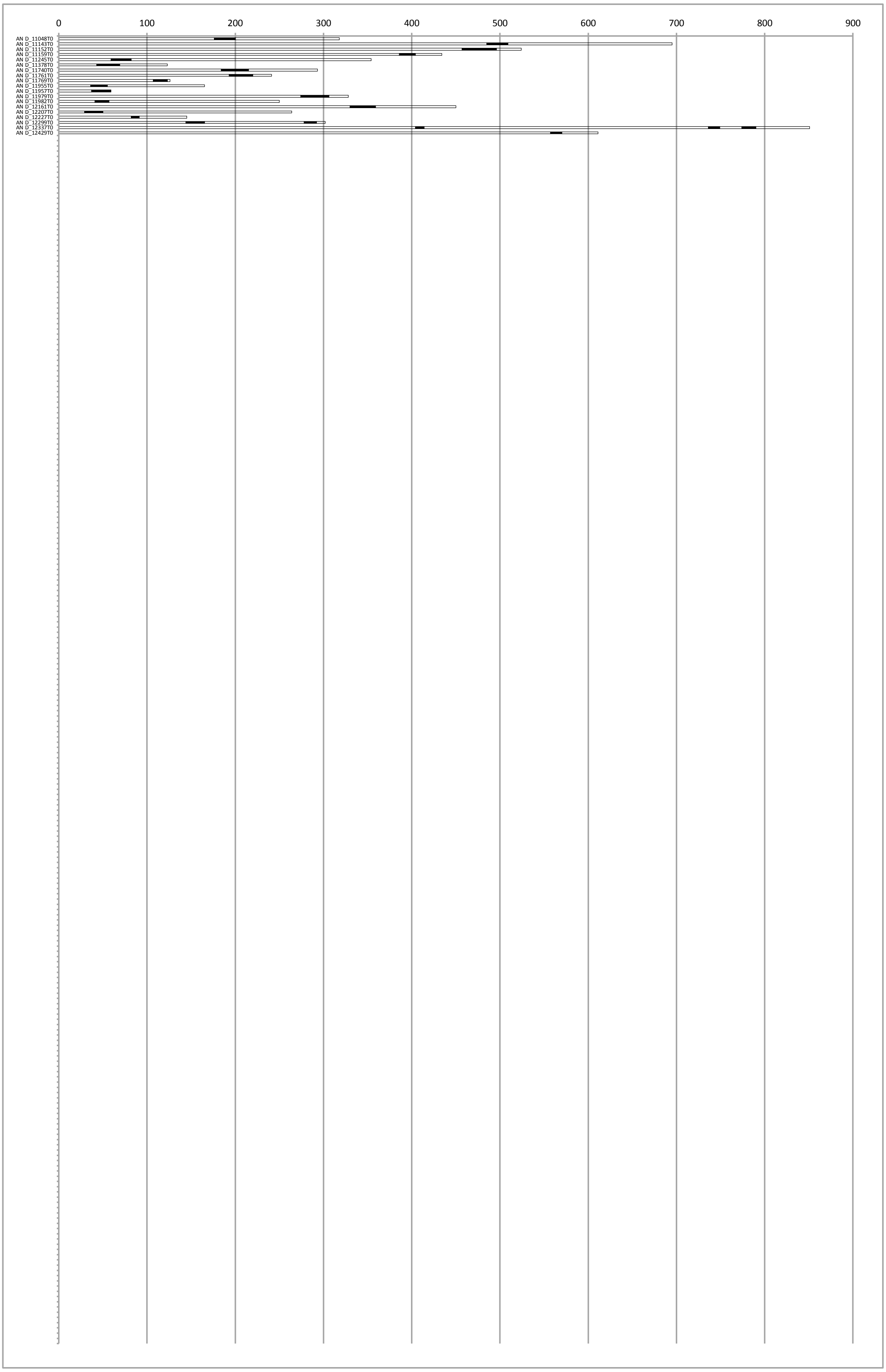
0
100
200
300
400
500
600
700
800
900
AN D_11048T0
AN D_11143T0
AN D_11152T0
AN D_11159T0
AN D_11245T0
AN D_11378T0
AN D_11740T0
AN D_11761T0
AN D_11769T0
AN D_11955T0
AN D_11957T0
AN D_11979T0
AN D_11982T0
AN D_12161T0
AN D_12207T0
AN D_12227T0
AN D_12299T0
AN D_12337T0
AN D_12429T0

HGR_detection V1.1 Report.	*M. grisea*

1. Parameters used in HGR detection

Glycosylation Density (%)	Window	Limit	Separator
25	20	5	5

2. Input protein set

Number of proteins	1400	Largest protein	5344 aa
Protein length average	385,10	Smallest protein	54 aa

3. HGRs found in the protein set

No. of proteins with HGRs	421	Number of HGRs	543
Average HGR length	36,90	Maximum HGR length	753
Average position of HGR centers (as % of protein lenght)	55,7	Minimum HGR length	6

4. Frequency distribution of the positions of HGR centers along the length of proteins

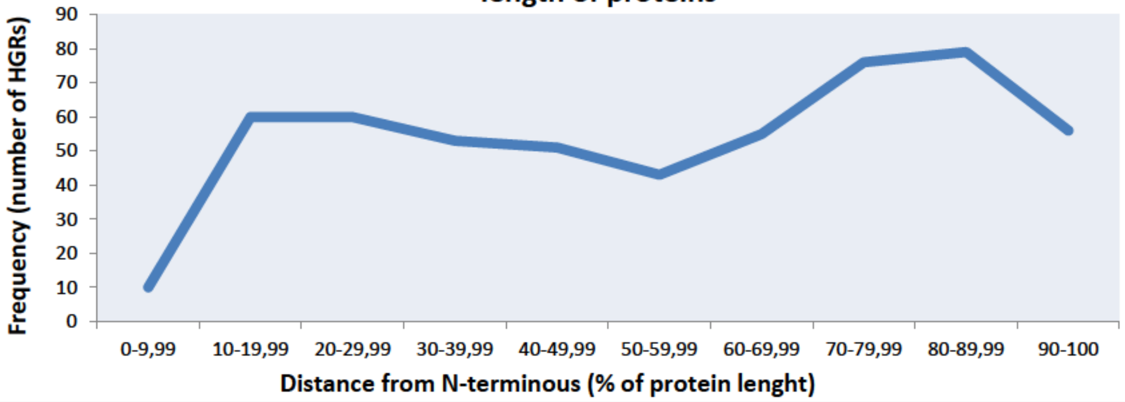

5. Frequency distribution of HGR lengths

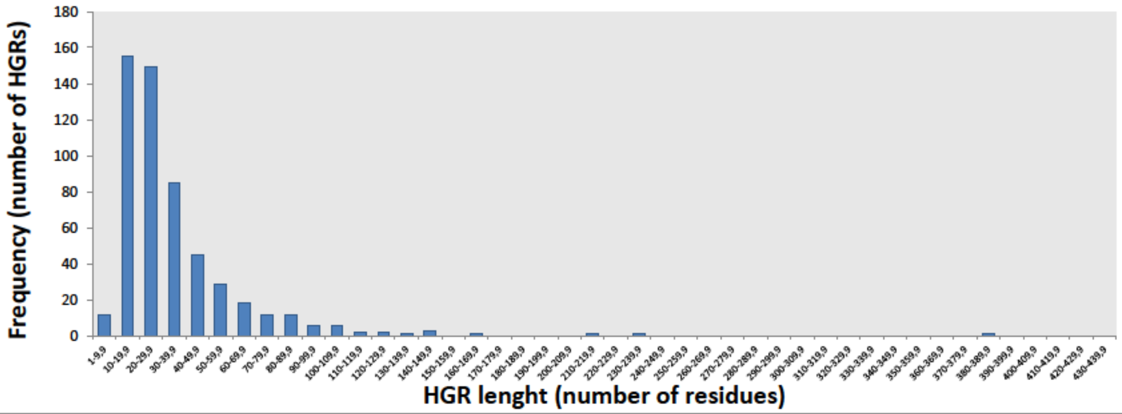

6. Alphabetical list of proteins with HGRs. Start and end positions of every HGR found. (Protein name: start-end. start-end. start-end. …)

MG00047.4 20-42.
MG00049.4 106-119.
MG00140.4 295-317.
MG00171.4 59-79.
MG00194.4 30-72.
MG00210.4 26-35.
MG00216.4 51-132.
MG00227.4 138-278. 387-411.
MG00235.4 132-140.
MG00259.4 40-60.
MG00319.4 389-418. 463-503.
MG00334.4 66-72.
MG00387.4 178-188. 285-310.
MG00390.4 201-226.
MG00469.4 85-97. 529-546. 580-595. 772-792.
MG00488.4 50-66.
MG00490.4 215-232.
MG00495.4 136-169.
MG00505.4 96-144.
MG00527.4 110-183.
MG00539.4 22-122.
MG00563.4 204-259. 293-317.
MG00592.4 288-344.
MG00627.4 22-54. 264-278.
MG00631.4 559-578.
MG00654.4 777-794.
MG00721.4 205-227. 257-280.
MG00737.4 44-71. 86-127.
MG00742.4 72-109.
MG00756.4 202-221.
MG00815.4 148-220.
MG00816.4 113-137.
MG00827.4 54-65.
MG00888.4 82-317. 444-562.
MG00916.4 30-55. 106-122.
MG01001.4 53-70.
MG01009.4 92-128.
MG01145.4 198-221.
MG01149.4 105-128.
MG01219.4 356-393.
MG01243.4 124-167. 244-279.
MG01247.4 66-102.
MG01265.4 171-188.
MG01275.4 228-238. 276-302. 468-486. 527-541.
MG01343.4 210-227.
MG01350.4 437-462.
MG01398.4 348-362.
MG01416.4 116-139.
MG01444.4 124-192.
MG01466.4 124-170.
MG01495.4 45-90.
MG01530.4 23-61. 226-240.
MG01532.4 20-28.
MG01609.4 19-34. 113-158.
MG01612.4 276-298. 349-390.
MG01655.4 250-264.
MG01657.4 180-209. 291-306.
MG01678.4 142-193.
MG01706.4 37-77.
MG01750.4 257-272. 497-516. 701-729.
MG01775.4 20-32.
MG01787.4 34-50.
MG01858.4 215-234. 785-811.
MG01861.4 25-147.
MG01868.4 289-329. 372-511. 548-634. 684-721.
MG01872.4 19-34. 125-150.
MG01875.4 38-57.
MG01890.4 320-332.
MG01905.4 350-367.
MG01914.4 210-251.
MG01963.4 47-66.
MG01977.4 46-72.
MG02082.4 33-82.
MG02097.4 176-194.
MG02106.4 96-111.
MG02111.4 34-60.
MG02142.4 78-91. 153-166. 226-367. 492-1013.
MG02148.4 420-489.
MG02166.4 24-60.
MG02176.4 123-153. 162-205.
MG02277.4 84-101.
MG02288.4 156-188.
MG02293.4 138-151.
MG02297.4 66-82.
MG02327.4 212-248.
MG02355.4 34-105.
MG02362.4 61-122.
MG02390.4 312-328.
MG02431.4 48-67. 80-109.
MG02508.4 168-196.
MG02540.4 22-65.
MG02546.4 24-37.
MG02562.4 43-67.
MG02564.4 29-53.
MG02588.4 25-39.
MG02602.4 29-79. 131-164.
MG02614.4 23-44.
MG02642.4 700-750. 779-822.
MG02645.4 26-63.
MG02654.4 382-408.
MG02754.4 459-484.
MG02756.4 207-222.
MG02758.4 37-50.
MG02771.4 569-587.
MG02839.4 654-666.
MG02884.4 303-329.
MG02898.4 72-95.
MG02957.4 91-154.
MG02981.4 57-67.
MG02984.4 358-364. 600-655.
MG03014.4 448-459.
MG03042.4 49-61.
MG03085.4 37-77.
MG03197.4 333-348.
MG03203.4 234-248.
MG03208.4 363-389.
MG03276.4 31-46. 259-285.
MG03342.4 26-56.
MG03354.4 22-186.
MG03368.4 132-214.
MG03394.4 81-107.
MG03408.4 136-239.
MG03417.4 139-251. 293-313.
MG03442.4 208-226.
MG03495.4 26-53.
MG03499.4 110-135.
MG03505.4 37-124. 169-216.
MG03544.4 68-90. 235-281. 311-323.
MG03620.4 515-522.
MG03691.4 133-155.
MG03706.4 76-122.
MG03742.4 603-654.
MG03746.4 282-304.
MG03747.4 45-63.
MG03750.4 66-96.
MG03760.4 41-54.
MG03767.4 42-60.
MG03784.4 145-229. 353-420. 536-547.
MG03815.4 154-178.
MG03849.4 109-124.
MG03887.4 406-424.
MG03946.4 31-52.
MG04172.4 189-229.
MG04202.4 128-143.
MG04206.4 193-205.
MG04218.4 71-126.
MG04237.4 287-317.
MG04262.4 256-286.
MG04290.4 139-156.
MG04306.4 148-174.
MG04325.4 156-185.
MG04412.4 194-254.
MG04490.4 213-244. 310-328.
MG04527.4 199-219.
MG04532.4 104-160. 405-437.
MG04541.4 870-930. 1050-1069.
MG04567.4 120-143.
MG04582.4 60 153. 168-268.
MG04583.4 34 82.
MG04599.4 117-205.
MG04669.4 499-518.
MG04689.4 317-366.
MG04691.4 161-231.
MG04734.4 21-47.
MG04737.4 56 102.
MG04749.4 301-322. 345-404.
MG04752.4 30 60.
MG04804.4 240-269. 408-438.
MG04828.4 94 106. 357-389.
MG04838.4 40 64.
MG04841.4 171-218.
MG04849.4 388-393.
MG04880.4 238-267.
MG04892.4 145-171.
MG04963.4 40-49.
MG05023.4 281-301.
MG05039.4 525-544. 700-719.
MG05051.4 161-190.
MG05054.4 111-118.
MG05069.4 29 86. 177-222.
MG05070.4 300-341.
MG05083.4 96 113.
MG05119.4 37-46. 74-91. 158-174.
MG05120.4 308-366.
MG05270.4 100-172.
MG05279.4 152-175.
MG05320.4 693-704.
MG05351.4 123-132.
MG05382.4 852-870. 1652-1659.
MG05393.4 150-229.
MG05402.4 452-485.
MG05418.4 79 104. 154-203.
MG05456.4 92 178.
MG05464.4 359-375.
MG05483.4 321-352.
MG05493.4 227-245.
MG05518.4 38 50.
MG05520.4 70 125.
MG05531.4 110-146.
MG05564.4 26 57.
MG05575.4 276-294.
MG05604.4 26 104.
MG05632.4 93 165.
MG05640.4 31 56.
MG05705.4 116-149.
MG05789.4 70 87. 159-182.
MG05832.4 82 90.
MG05874.4 262-305.
MG05875.4 18 32.
MG05885.4 161-183.
MG05913.4 85 118.
MG05932.4 387-425.
MG05978.4 94 124.
MG06008.4 216-249.
MG06027.4 90 107.
MG06033.4 46 261.
MG06069.4 375-399. 432-463.
MG06101.4 248-270.
MG06116.4 188-254.
MG06123.4 61 78.
MG06155.4 138-161. 423-507.
MG06166.4 204-225.
MG06207.4 61 81. 437-471.
MG06216.4 94 119.
MG06224.4 26-45.
MG06275.4 43 70.
MG06412.4 40 84.
MG06418.4 473-500.
MG06477.4 503-554. 582-605. 1144-1167.
MG06478.4 85 97.
MG06538.4 135-157.
MG06593.4 223-253.
MG06648.4 614-629.
MG06653.4 148-208.
MG06714.4 44 59.
MG06755.4 474-491.
MG06771.4 68 97.
MG06773.4 41 793.
MG06775.4 123-197. 212-246.
MG06786.4 265-291. 443-462.
MG06803.4 281-298.
MG06834.4 416-431.
MG06835.4 46 64.
MG06842.4 681-736.
MG06849.4 196-217.
MG06930.4 190-205. 238-254.
MG06953.4 50 73. 115-131.
MG06955.4 170-199.
MG06988.4 199-225. 305-327.
MG07005.4 116-207. 238-257.
MG07096.4 101-120.
MG07100.4 111-175.
MG07179.4 442-486.
MG07194.4 30 59.
MG07220.4 554-580.
MG07225.4 41 53.
MG07292.4 27 135. 158-170.
MG07303.4 24 39.
MG07306.4 344-730.
MG07312.4 784-795.
MG07334.4 506-537.
MG07348.4 50 72.
MG07353.4 143-172.
MG07362.4 164-190.
MG07414.4 211-303.
MG07566.4 51 64.
MG07568.4 339-354.
MG07571.4 20-41.
MG07575.4 694-729.
MG07577.4 583-600.
MG07598.4 24-45. 142-175.
MG07607.4 68 124.
MG07609.4 61 94.
MG07623.4 22 55.
MG07644.4 27 76. 294-313.
MG07684.4 141-170.
MG07716.4 259-283.
MG07748.4 133-148.
MG07775.4 24-47. 144-197.
MG07786.4 23 38.
MG07807.4 21 38.
MG07842.4 105-121.
MG07880.4 134-143.
MG07924.4 335-354.
MG07972.4 102-124.
MG08045.4 408-451.
MG08109.4 131-150.
MG08120.4 21-40.
MG08125.4 128-153. 176-210.
MG08158.4 152-168.
MG08165.4 598-611.
MG08200.4 548-577. 684-705.
MG08210.4 259-277.
MG08231.4 332-353.
MG08232.4 50 68.
MG08252.4 451-473.
MG08253.4 62 89.
MG08264.4 35 65.
MG08276.4 126-189. 244-285.
MG08321.4 97 176.
MG08328.4 241-270.
MG08334.4 46 104. 144-192.

MG08348.4 91-177.
MG08351.4 188-220.
MG08401.4 223-229.
MG08406.4 63-94. 132-167.
MG08408.4 262-315.
MG08409.4 237-249.
MG08433.4 30-49. 78-97.
MG08441.4 29-50.
MG08442.4 25-36. 122-153.
MG08467.4 138-152. 339-352.
MG08491.4 35-56. 77-98.
MG08501.4 55-74.
MG08506.4 26-59.
MG08507.4 45-63.
MG08523.4 400-417.
MG08544.4 124-135.
MG08559.4 45-70.
MG08561.4 151-169.
MG08577.4 65-120. 154-173.
MG08580.4 481-523.
MG08593.4 293-312.
MG08607.4 28-40.
MG08647.4 153-194. 252-295. 325-343. 361-382.
MG08698.4 416-442.
MG08729.4 107-142. 185-228.
MG08754.4 57-74. 157-226.
MG08772.4 230-275.
MG08773.4 134-158. 391-409.
MG08774.4 283-336.
MG08798.4 37-52. 167-195.
MG08799.4 29-42.
MG08811.4 22-49. 167-178.
MG08812.4 21-39. 159-174.
MG08819.4 30-58.
MG08824.4 122-187.
MG08940.4 158-171.
MG08941.4 27-60.
MG08944.4 91-138.
MG08946.4 25-55.
MG08960.4 296-335.
MG08999.4 165-184.
MG09036.4 333-355.
MG09079.4 181-205.
MG09094.4 69-90.
MG09106.4 20-36.
MG09159.4 370-392. 454-508.
MG09180.4 31-85.
MG09205.4 692-718.
MG09210.4 374-473.
MG09221.4 819-830. 934-976.
MG09237.4 82-158. 188-242.
MG09252.4 24-119.
MG09258.4 29-95.
MG09268.4 131-146.
MG09271.4 77-88.
MG09395.4 340-401. 487-501. 518-539. 574-639. 690 706.
MG09412.4 185-203.
MG09419.4 87-124. 193-208.
MG09425.4 23-41.
MG09460.4 189-232.
MG09488.4 169-185.
MG09506.4 120-129.
MG09527.4 222-250. 285-320.
MG09570.4 65-94.
MG09576.4 58-68.
MG09604.4 360-372. 415-423.
MG09629.4 84-101.
MG09641.4 141-235.
MG09714.4 408-446. 545-563.
MG09717.4 198-232.
MG09736.4 112-185.
MG09738.4 54-72.
MG09742.4 44-54. 110-133.
MG09765.4 41-68.
MG09792.4 346-395.
MG09804.4 45-73. 131-156.
MG09807.4 124-186.
MG09840.4 247-281.
MG09851.4 174-189.
MG09938.4 86-121. 221-349.
MG09974.4 50-85.
MG09985.4 353-365.
MG09989.4 640-677.
MG09998.4 33-52.
MG10001.4 25-42.
MG10004.4 111-130.
MG10024.4 74-105. 208-224.
MG10065.4 41-60.
MG10066.4 229-294.
MG10097.4 22-44.
MG10105.4 20-46.
MG10169.4 163-194.
MG10191.4 254-322. 424-450.
MG10206.4 47-81.
MG10208.4 222-262.
MG10310.4 22-50.
MG10317.4 42-62. 136-154. 199-332. 354-460.
MG10361.4 159-185.
MG10424.4 18-37.
MG10425.4 75-90. 162-179.
MG10431.4 373-410. 472-508. 593-609.
MG10467.4 28-59. 120-152.
MG10471.4 23-39. 156-183.
MG10515.4 21-36.
MG10621.4 448-469.
MG10657.4 117-130.
MG10679.4 73-87.
MG10708.4 155-173.
MG10725.4 71-88.
MG10789.4 117-156.
MG10790.4 119-215.
MG10824.4 140-178.
MG10865.4 87-168. 198-237. 264 288. 310-323. 357-376. 396-415. 478-498. 528-584. 648-679.
MG10895.4 199-239.
MG10995.4 181-220.
MG11009.4 329-353.
MG11020.4 388-393.
MG11044.4 412-425.
MG11072.4 26-56.

7. Graphical representation of all HGRs found in the protein set

Proteins are represented as boxes of lenght proportional to protein length. Black regions indicate HGRs

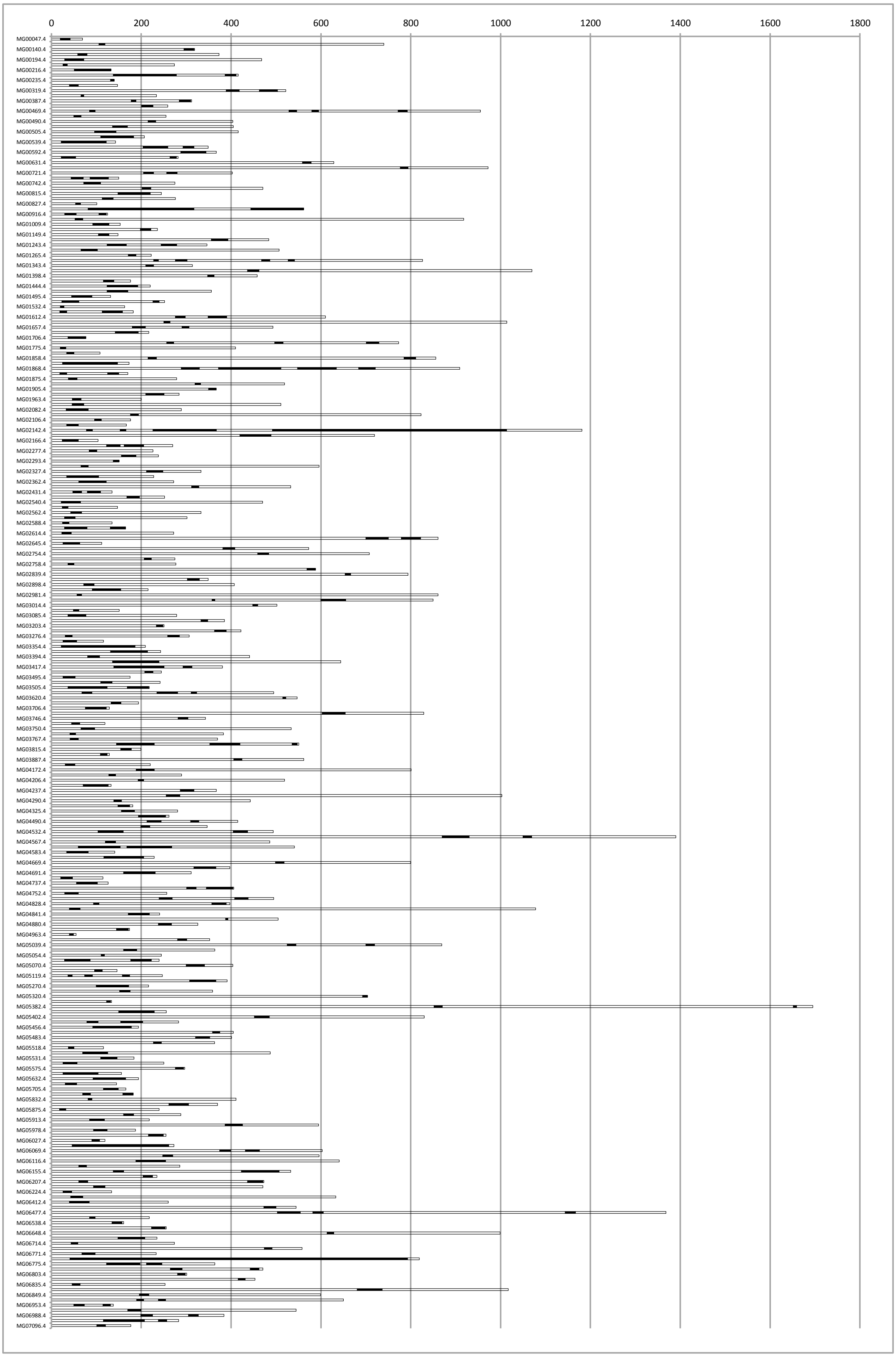

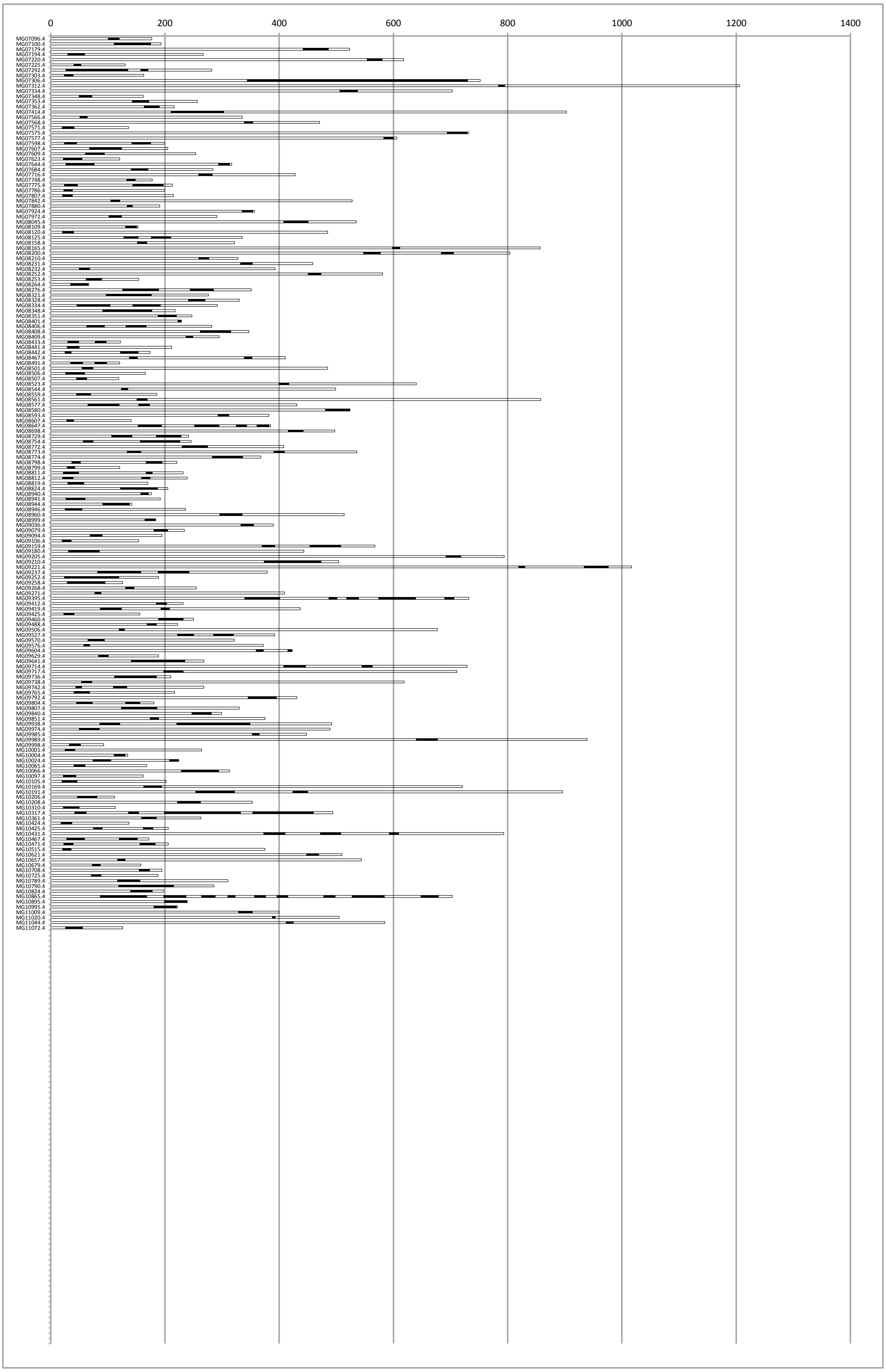

HGR_detection V1.1 Report.	*N. crassa*

1. Parameters used in HGR detection

Glycosylation Density (%)	Window	Limit	Separator
25	20	5	5

2. Input protein set

Number of proteins	929	Largest protein	3054 aa
Protein length average	440,83	Smallest protein	46 aa

3. HGRs found in the protein set

No. of proteins with HGRs	389	Number of HGRs	538
Average HGR length	38,80	Maximum HGR length	622
Average position of HGR centers (as % of protein lenght)	53,9	Minimum HGR length	5

4. Frequency distribution of the positions of HGR centers along the length of proteins

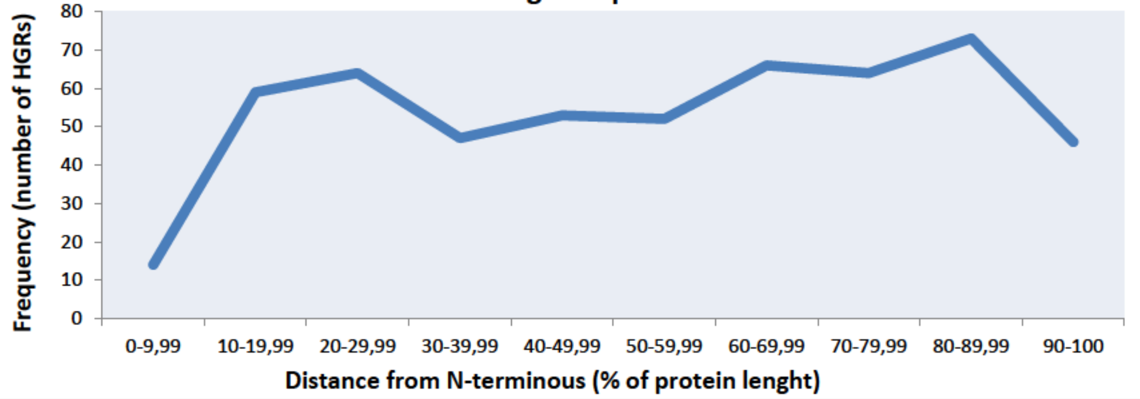

5. Frequency distribution of HGR lengths

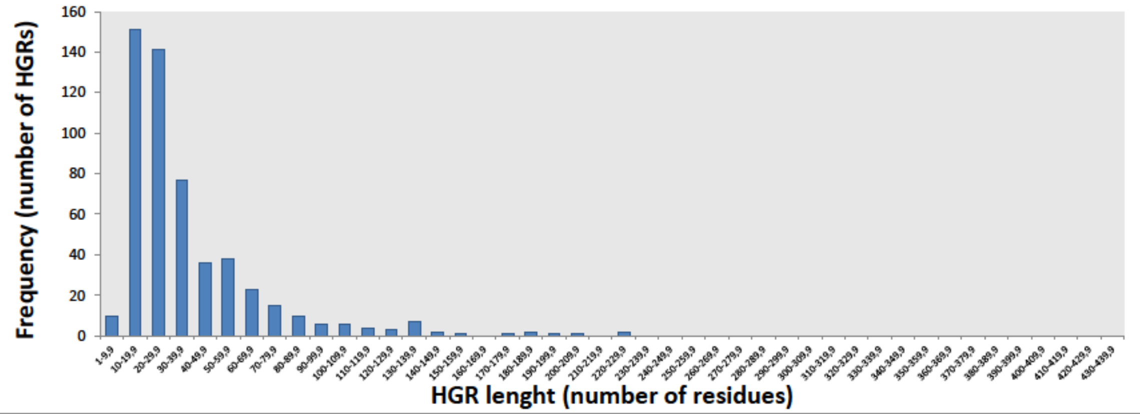

6. Alphabetical list of proteins with HGRs. Start and end positions of every HGR found.
(Protein name: start-end. start-end. start-end. …)

NCU00119T0 533-558.
NCU00175T0 47-97. 118-145. 224-253.
NCU00207T0 109-123.
NCU00265T0 103-122.
NCU00267T0 75-121.
NCU00270T0 103-131. 255-269.
NCU00309T0 405-435.
NCU00322T0 26-72. 98-136.
NCU00363T0 688-720.
NCU00459T0 2843-2871.
NCU00473T0 43-58.
NCU00607T0 19-32.
NCU00609T0 30-52.
NCU00630T0 367-400. 457-477.
NCU00694T0 142-160. 490-500.
NCU00708T0 129-149.
NCU00710T0 323-351.
NCU00729T0 121-185.
NCU00762T0 59-83.
NCU00798T0 24-150.
NCU00811T0 52-78.
NCU00813T0 299-320.
NCU00822T0 301-359. 376-394.
NCU00826T0 98-119. 385-395.
NCU00836T0 223-291.
NCU00957T0 113-191.
NCU00968T0 29-46. 228-263. 327-345.
NCU01045T1 81-135.
NCU01056T0 37-58.
NCU01063T0 30-45.
NCU01158T0 158-244.
NCU01162T0 398-429.
NCU01214T0 101-192. 317-341. 367-562.
NCU01267T0 448-460.
NCU01296T0 222-251.
NCU01319T0 471-496.
NCU01351T0 110-294. 312-344.
NCU01353T0 343-364. 465-478. 587-605.
NCU01380T0 246-270. 285-341.
NCU01394T0 404-420. 853-872.
NCU01395T0 421-434.
NCU01403T0 253-300.
NCU01418T0 24-117.
NCU01425T0 187-245.
NCU01462T0 258-277.
NCU01521T0 45-61.
NCU01568T0 84-104.
NCU01699T0 138-158. 312-322.
NCU01720T0 63-98. 113-133.
NCU01750T0 152-163.
NCU01752T0 187-204. 363-412. 482-533.
NCU01769T0 207-240. 402-417.
NCU01812T0 189-207. 223-288. 458-474.
NCU01947T0 893-911.
NCU01964T0 641-678.
NCU01988T0 80-92.
NCU01990T0 380-388.
NCU02021T0 23-42.
NCU02028T0 56-75.
NCU02041T0 124-172.
NCU02064T0 56-94.
NCU02087T0 93-169. 200-288. 431-453. 491-526. 545-579.
NCU02099T0 99-115.
NCU02119T0 798-852.
NCU02138T0 148-186.
NCU02184T0 1049-1078. 1119-1138. 1373 1401.
NCU02187T0 54-146. 171-202.
NCU02189T0 23-42.
NCU02192T0 61-76.
NCU02197T0 31-43.
NCU02199T0 45-57. 85-112.
NCU02212T0 28-48.
NCU02216T0 25-44.
NCU02240T0 236-275.
NCU02246T0 274-499. 526-545. 640-659. 680-702.
NCU02277T0 167-183.
NCU02336T0 79-118.
NCU02338T0 16-32.
NCU02344T0 248-281.
NCU02470T0 72-147.
NCU02561T0 31-88.
NCU02583T0 561-587.
NCU02584T0 156-191.
NCU02668T0 114-160.
NCU02681T0 201-215.
NCU02722T0 156-192.
NCU02732T0 234-256.
NCU02745T0 121-157. 341-373.
NCU02865T0 32-47. 174-201.
NCU02869T0 266-287.
NCU02879T0 38-52.
NCU02880T0 47-65. 119-143.
NCU02883T0 27-45. 185-237. 273-293. 357-373.
NCU02916T0 242-307.
NCU02940T0 77-86.
NCU02997T0 186-201.
NCU03013T0 198-218.
NCU03083T0 101-110. 172-183.
NCU03104T0 1171-1187.
NCU03168T0 464-493.
NCU03210T0 38-73. 129-267. 301-348.
NCU03219T0 635-697.
NCU03222T0 128-149.
NCU03222T1 128-149.
NCU03287T0 42-66.
NCU03293T0 27-148.
NCU03318T0 23-49. 295-315. 392-410.
NCU03369T0 59-75.
NCU03456T0 29-78.
NCU03498T0 446-455.
NCU03511T0 160-177.
NCU03530T0 107-242.
NCU03549T0 120-154.
NCU03602T0 97-125.
NCU03646T0 561-612.
NCU03707T0 87-102.
NCU03736T0 93-117.
NCU03740T0 551-580.
NCU03742T0 127-147.
NCU03767T0 178-203. 729-763.
NCU03873T0 93-152.
NCU04033T0 243-282.
NCU04064T0 351-377.
NCU04066T0 338-374.
NCU04070T0 163-228.
NCU04133T0 630-679.
NCU04134T0 33-50.
NCU04160T0 33-92. 281-308.
NCU04170T0 1190-1204. 1328-1352. 1489 1521. 1613-1628. 1690-1827.
NCU04286T0 168-188.
NCU04373T0 109-144. 191-252. 268-318. 435-481. 596-616. 657-676. 740-781.
NCU04464T0 158-217. 313-329. 401-422. 446-471.
NCU04471T0 103-117.
NCU04493T0 97-152.
NCU04496T0 154-199.
NCU04501T0 31-48. 142-169.
NCU04524T0 55-88.
NCU04542T0 128-156.
NCU04543T0 154-168. 248-282.
NCU04588T0 44-72.
NCU04603T0 58-160.
NCU04616T0 102-215. 461-538.
NCU04633T0 688-717.
NCU04732T0 480-509. 556-574.
NCU04752T0 94-113.
NCU04774T0 536-558. 590-604.
NCU04853T0 33-152.
NCU04872T0 180-199.
NCU04900T0 30-76.
NCU04932T0 86-139. 195-255.
NCU04948T0 61-85. 268-343.
NCU04953T0 84-116.
NCU04997T0 338-377.
NCU04998T0 22-43.
NCU05042T0 580-616.
NCU05043T0 435-470.
NCU05048T0 82-114. 175-200. 378-442.
NCU05054T0 1657-1688.
NCU05074T0 315-347.
NCU05096T0 89-120.
NCU05119T0 96-113.
NCU05121T0 233-258.
NCU05143T0 31-76.
NCU05152T0 68-93. 161-190.
NCU05155T0 37-60. 101-148. 168-238.
NCU05158T0 47-109. 130-155. 197-206. 228-248. 302-312.
NCU05159T0 246-262.
NCU05190T0 71-89. 130-180.
NCU05191T0 191-277.
NCU05212T0 184-214.
NCU05229T0 55-254.
NCU05373T0 74-88.
NCU05395T0 20-96.
NCU05404T0 230-278. 324-387.
NCU05547T0 57-72.
NCU05609T0 230-317.
NCU05618T0 82-102. 165-182.
NCU05639T0 155-159.
NCU05667T0 124-196.
NCU05686T0 392-411.
NCU05707T0 22-37.
NCU05812T0 205-258.
NCU05814T0 96-196.
NCU05827T0 131-153.
NCU05872T0 196-217.
NCU05915T0 173-214.
NCU05916T0 58-73.
NCU05917T0 128-139. 183-199. 275-286.
NCU05923T0 234-267.
NCU05932T0 289-317. 333-366.
NCU05940T0 41-85. 163-189. 251-295.
NCU05955T0 750-812.
NCU05969T0 261-303.
NCU05974T0 279-315.
NCU06010T0 508-544.
NCU06041T0 241-259.
NCU06051T0 141-152. 187-233.
NCU06098T0 548-572.
NCU06109T0 310-461. 490-509. 556-607. 696-728. 800-848. 1121-1129.
NCU06117T0 343-402.
NCU06169T0 274-292.
NCU06185T0 33-174. 194-228.
NCU06381T0 350-376.
NCU06391T0 345-351.
NCU06436T0 475-612.
NCU06524T0 31-53.
NCU06525T0 213-275. 316-460.
NCU06541T0 46-77.
NCU06547T0 74-84. 222-330.
NCU06602T0 197-281. 386-408.
NCU06607T0 323-366.
NCU06772T0 55-77. 573-597.
NCU06781T0 432-477.
NCU06792T0 83-95.
NCU06817T0 155-172.
NCU06839T0 321-372.
NCU06850T0 385-402.
NCU06910T0 162-193.
NCU06910T1 162-193.
NCU06911T0 21-27.
NCU06912T0 38-54.
NCU06934T0 245-255.
NCU06937T0 25-39.
NCU06967T0 394-409.
NCU06981T0 128-198.
NCU06983T0 196-255.
NCU06991T0 422-439.
NCU07017T0 131-152. 242-296.
NCU07051T0 49-59.
NCU07061T0 27-77.
NCU07063T0 466-483.
NCU07095T0 35-55.
NCU07121T0 658-679.
NCU07136T0 152-175.
NCU07141T0 87-96.
NCU07163T0 26-156.
NCU07164T0 33-162.
NCU07169T0 280-293.
NCU07225T0 229-248.
NCU07253T0 406-432.
NCU07275T0 129-165.
NCU07277T0 83-704.
NCU07340T0 447-485.
NCU07355T0 314-333.
NCU07388T0 160-215. 397-432.
NCU07454T0 103-164.
NCU07455T0 158-171. 206-218.
NCU07486T0 81-133. 189-211.
NCU07520T0 271-302.
NCU07533T0 365-412. 485-489.
NCU07537T0 68-74. 307-325. 404-419. 463-494.
NCU07545T0 340-355.
NCU07581T0 32-63.
NCU07627T0 28-55.
NCU07628T0 28-80.
NCU07635T0 106-122.
NCU07649T0 386-399.
NCU07695T0 117-134. 272-293.
NCU07695T1 121-138. 276-297.
NCU07709T0 386-402.
NCU07760T0 255-321.
NCU07776T0 97-164.
NCU07813T0 287-301.
NCU07827T0 219-276.
NCU07881T0 47-123.
NCU07910T0 208-225.
NCU07923T0 26-45.
NCU07974T0 404-432.
NCU07999T0 798-805.
NCU08038T0 204-219.
NCU08083T0 52-81.
NCU08084T0 28-35. 67-84.
NCU08085T0 146-190. 254-326.
NCU08100T0 36-64.
NCU08127T0 421-442.
NCU08176T0 22-44.
NCU08180T0 621-640.
NCU08193T0 174-202.
NCU08219T0 89-135.
NCU08230T0 70-96.
NCU08318T0 37-56. 128-145. 179-210.
NCU08371T0 583-605.
NCU08442T0 84-121.
NCU08447T0 306-320.
NCU08454T0 59-86.
NCU08473T0 803-814. 1575-1600.
NCU08523T0 27-85.
NCU08629T0 86-136. 247-267. 309-326. 400-436.
NCU08635T0 48-66.
NCU08646T0 25-45.
NCU08649T0 82-95. 128-153. 229-243.
NCU08654T0 128-157.
NCU08680T0 58-78.
NCU08720T0 130-146.
NCU08746T0 263-283.
NCU08756T0 37-93. 251-267.
NCU08760T0 259-279.
NCU08761T0 26-55.
NCU08773T0 114-129. 195-218.
NCU08810T0 26-38.
NCU08819T0 126-148.
NCU08820T0 84-98. 199-245. 338-524. 623-641.

NCU08821T0	734-752.
NCU08905T0	562-580.
NCU08916T0	167-178.
NCU08926T0	434-450.
NCU08982T0	26-32.
NCU09026T0	41-56.
NCU09029T0	18-36. 84-93. 167 187.
NCU09042T0	844-855.
NCU09098T0	244-265.
NCU09099T0	32-258.
NCU09133T0	132-233.
NCU09155T0	444-462.
NCU09175T0	314-387.
NCU09213T0	53-73. 86-106. 204-230.
NCU09223T0	481-501.
NCU09236T0	55-72.
NCU09263T0	116-173.
NCU09267T0	364-380.
NCU09280T0	91-111.
NCU09326T0	53-190. 225-250. 268-351.
NCU09383T0	27-36. 123-215.
NCU09386T0	37-49.
NCU09416T0	66-118.
NCU09428T0	199-254.
NCU09431T0	48-101.
NCU09464T0	20-36.
NCU09484T0	333-353.
NCU09485T0	609-627. 804-822.
NCU09487T0	68-106. 122-153. 214-236.
NCU09492T0	1856-1867.
NCU09498T0	28-75. 118-159. 198-232.
NCU09524T0	75-144.
NCU09526T0	571-588.
NCU09562T0	169-185.
NCU09564T0	314-333.
NCU09620T0	36-58.
NCU09672T0	301-320.
NCU09680T0	69-121.
NCU09685T0	21-46.
NCU09718T0	32-45.
NCU09729T0	261-278. 833-843.
NCU09733T0	330-447.
NCU09734T0	144-157.
NCU09755T0	59-93.
NCU09764T0	255-357.
NCU09765T0	79-129.
NCU09785T0	460-476.
NCU09786T0	192-205.
NCU09791T0	98-138.
NCU09848T0	21-35.
NCU09851T0	68-149. 200-229.
NCU09868T0	46-108.
NCU09929T0	147-232.
NCU09964T0	21-33.
NCU09990T0	178-201.
NCU10003T0	178-187.
NCU10014T0	50-143. 194-274.
NCU10039T0	93-118.
NCU10152T0	23-102.
NCU10264T0	653-691. 1197-1219.
NCU10641T0	38-53.
NCU10687T0	175-288. 313-337. 396-427. 507-534. 568-638.
NCU10702T0	43-71.
NCU10861T0	27-47.
NCU10866T0	21-50.
NCU10893T0	23-84.
NCU10907T0	462-492.
NCU11429T0	118-162. 213-249.
NCU11430T0	69-85.
NCU11432T0	24-95.
NCU11550T0	99-114.
NCU11575T0	132-178. 212-243.
NCU11583T0	28-79. 170-183.
NCU11598T0	512-529.
NCU11753T0	294-308.
NCU11766T0	62-236.
NCU11787T0	22-60. 142-167.
NCU11797T0	62-77. 193-294.
NCU11800T0	102-153. 221-244.
NCU11801T0	443-457.
NCU11828T0	295-338.
NCU11857T0	119-129.
NCU11860T0	90-106.
NCU11888T0	67-93.
NCU12063T0	56-166. 184-228.
NCU12122T0	104-118.

253-342. 443-497.

7. Graphical representation of all HGRs found in the protein set

Proteins are represented as boxes of lenght proportional to protein length. Black regions indicate HGRs

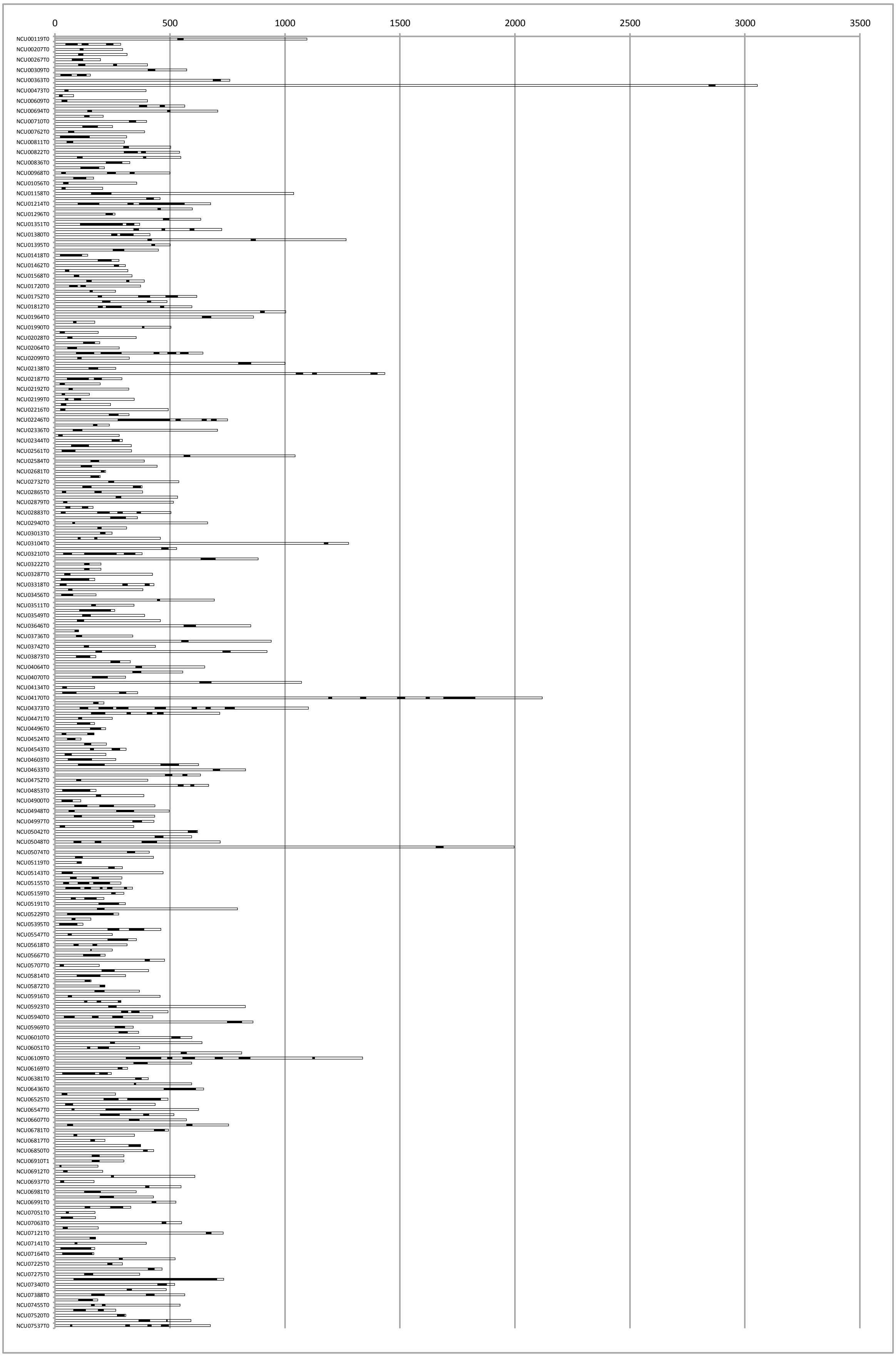

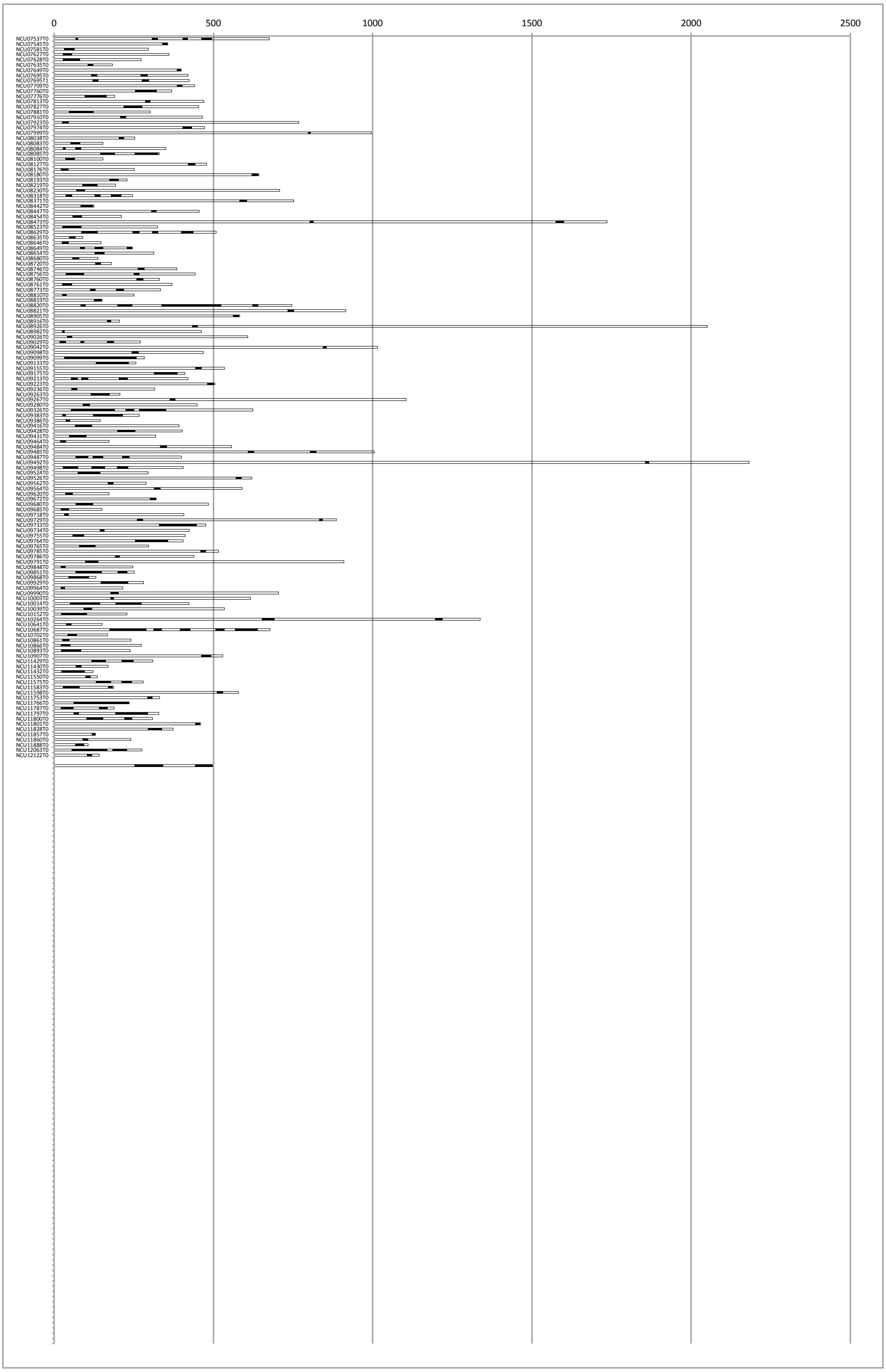
0
500
1000
1500
2000
2500

HGR_detection V1.1 Report.	*S. cerevisiae*

1. Parameters used in HGR detection			
**Glycosylation Density (%)**	**Window**	**Limit**	**Separator**
25	20	5	5

2. Input protein set			
Number of proteins	250	Largest protein	2628 aa
Protein length average	445,83	Smallest protein	39 aa

3. HGRs found in the protein set			
No. of proteins with HGRs	108	Number of HGRs	174
Average HGR length	66,92	Maximum HGR length	821
Average position of HGR centers (as % of protein lenght)	53,0	Minimum HGR length	7

4. Frequency distribution of the positions of HGR centers along the length of proteins

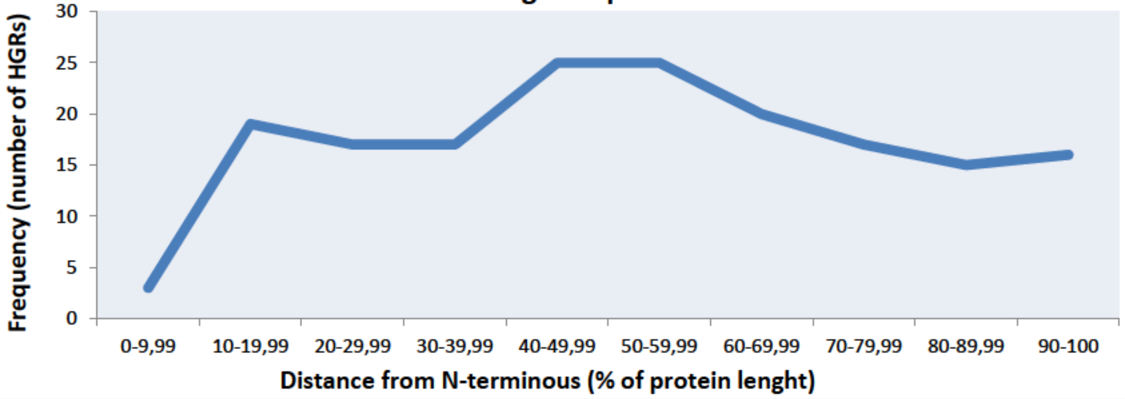

5. Frequency distribution of HGR lengths

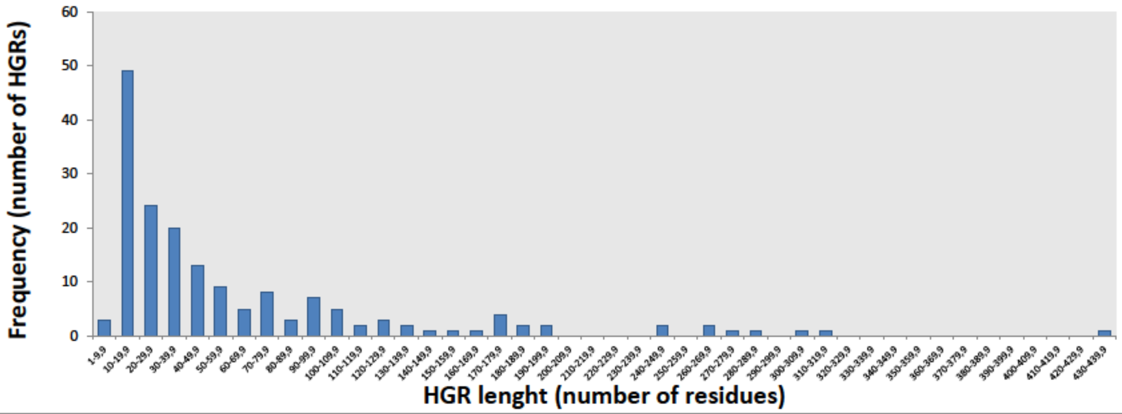

6. Alphabetical list of proteins with HGRs. Start and end positions of every HGR found. (Protein name: start-end. start-end. start-end. …)

YAL063C 275-305. 365-380. 503-515. 548-560. 575-620. 638-650. 683-695. 728-742. 773-845. 918-1014. 1200-
YAR050W 275-305. 368-380. 544-560. 589-607. 683-713. 773-836. 863-881. 908-925. 953-971. 998-1061. 1134-
YAR066W 30-75.
YBR067C 110-185.
YBR078W 362-384.
YBR162C 156-219.
YCL027W 36-46.
YCL048W-A 27-61.
YCR068W 482-501.
YCR089W 847-877. 1245-1319.
YDL218W 244-259.
YDL222C 256-306.
YDR055W 405-416.
YDR077W 23-300.
YDR144C 526-571.
YDR349C 27-39.
YDR420W 278-298. 461-773.
YDR524C-B 23-41.
YDR534C 58-91. 136-161. 206-238. 271-447.
YEL040W 352-438.
YER011W 114-217.
YER150W 33-124.
YFL021W 290-304.
YFL067W 27-87.
YGL028C 99-168. 205-278.
YGL032C 22-44.
YGL228W 568-576.
YGL259W 119-136.
YGR014W 169-183. 583-608. 630-649. 708-822.
YGR023W 44-307.
YGR189C 25-49. 306-464.
YGR279C 64-122.
YHL028W 123-314. 469-484. 507-521.
YHR126C 114-129.
YHR139C 198-207.
YHR143W 77-273. 311-324.
YHR195W 250-265.
YHR211W 272-290. 320-620. 683-786.
YHR214W 30-75.
Y L011W 119-240.
Y L037C 69-85.
Y L123W 56-79. 111-201.
Y L140W 455-484.
Y L169C 39-286. 598-643. 788-903.
Y R019C 214-1034.
YJL078C 164-342. 456-485. 552 566. 703-715. 772-788.
YJL079C 49-155.
YJL116C 53-72.
YJL158C 25-47. 82-122.
YJL159W 28-67. 296-308.
YJL160C 128-158.
YJL174W 142-181.
YJR004C 376-390. 523-540.
YJR150C 119-263.
YJR151C 123-554. 668-719. 866-895.
YKL096W 130-201.
YKL096W-A 30-69.
YKL164C 28-45. 209-240.
YKR013W 54 68. 96-189.
YKR042W 57 113.
YKR058W 300-319.
YKR102W 339-371. 402-418. 465-497. 528-555. 591-620. 688-716. 798-815.
YLR042C 70-137.
YLR059C 246-267.
YLR110C 23-111.
YLR121C 443-466.
YLR194C 40-218.
YLR286C 316-484.
YLR332W 41-174.
YLR356W 32-48.
YLR390W-A 89-213.
YMR006C 627-668.
YMR123W 107-119.
YMR148W 90-115.
YMR200W 185-197.
YMR215W 461-492.
YMR305C 63-132.
YMR307W 487-522.
YNL019C 34-45.
YNL033W 34-45.
YNL066W 64 146.
YNL190W 28-48.
YNL194C 233-252.
YNL238W 625-666.
YNL283C 122-302.
YNL300W 21 77.
YNL322C 44-232.
YNL327W 379-414. 477-488. 610-618. 986-1003.
YNR041C 35-54.
YNR044W 149-388. 607-680.
YNR067C 305-318. 373-398.
YOL007C 38-88.
YOL011W 630-651.
YOL030W 404-451.
YOL060C 569-575.
YOL073C 267-297.
YOL105C 148-319.
YOL155C 57-321. 630-688. 721-738. 802-852. 887-909.
YOR008C 119-209.
YOR009W 115-400.
YOR010C 112-208.
YOR214C 168-206.
YOR247W 25-123. 159-208.
YOR382W 26-129.
YOR383C 54-181.
YPL156C 114-129.
YPL163C 26-164. 216-258.
YPR027C 35-54.

7. Graphical representation of all HGRs found in the protein set

Proteins are represented as boxes of lenght proportional to protein length. Black regions indicate HGRs

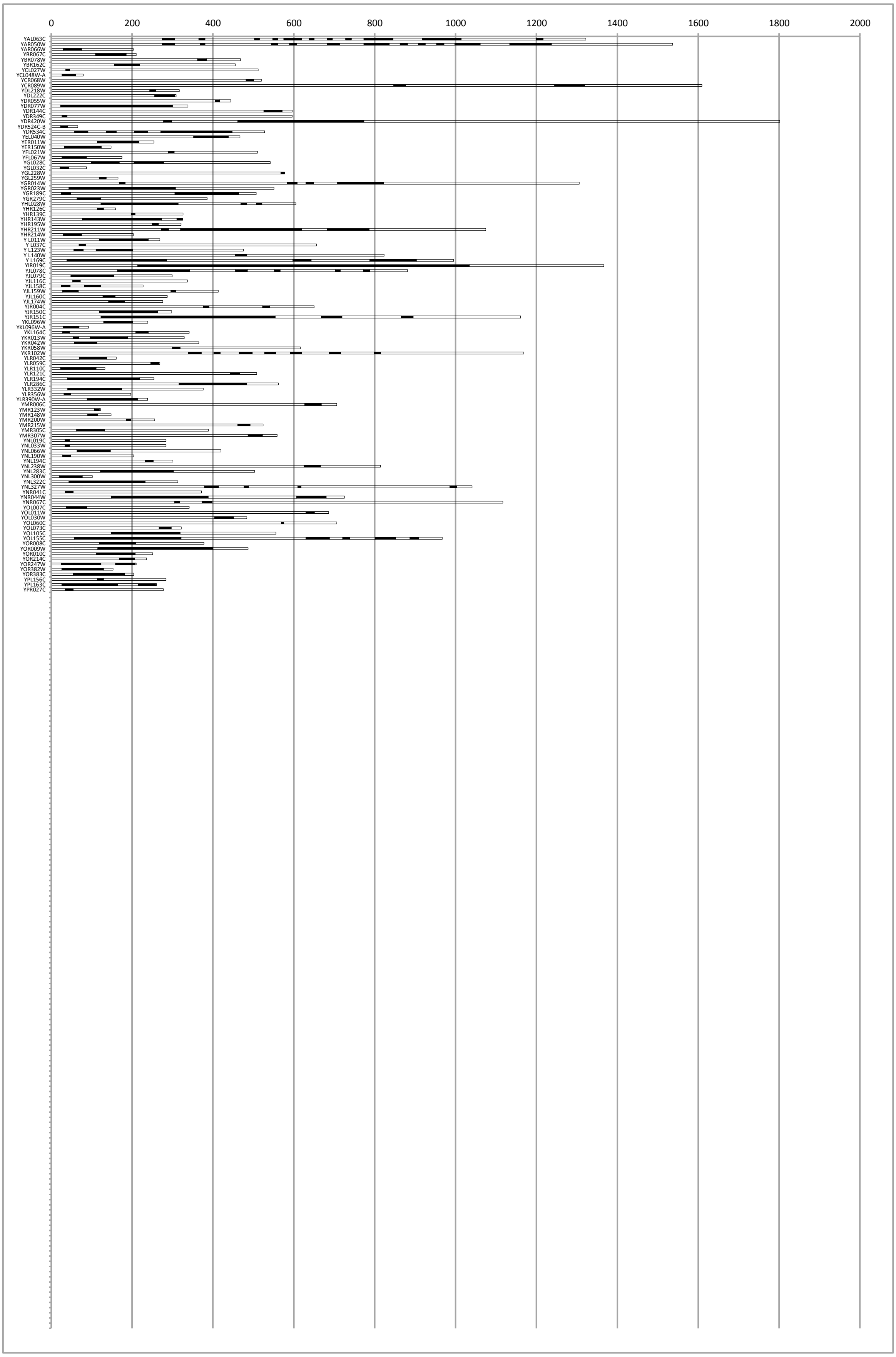

HGR_detection V1.1 Report.	*S. sclerotiorum*

1. Parameters used in HGR detection			
**Glycosylation Density (%)**	**Window**	**Limit**	**Separator**
25	20	5	5

2. Input protein set			
Number of proteins	913	Largest protein	3252 aa
Protein length average	399,43	Smallest protein	33 aa

3. HGRs found in the protein set			
No. of proteins with HGRs	356	Number of HGRs	512
Average HGR length	45,83	Maximum HGR length	361
Average position of HGR centers (as % of protein lenght)	55,5	Minimum HGR length	5

4. Frequency distribution of the positions of HGR centers along the length of proteins

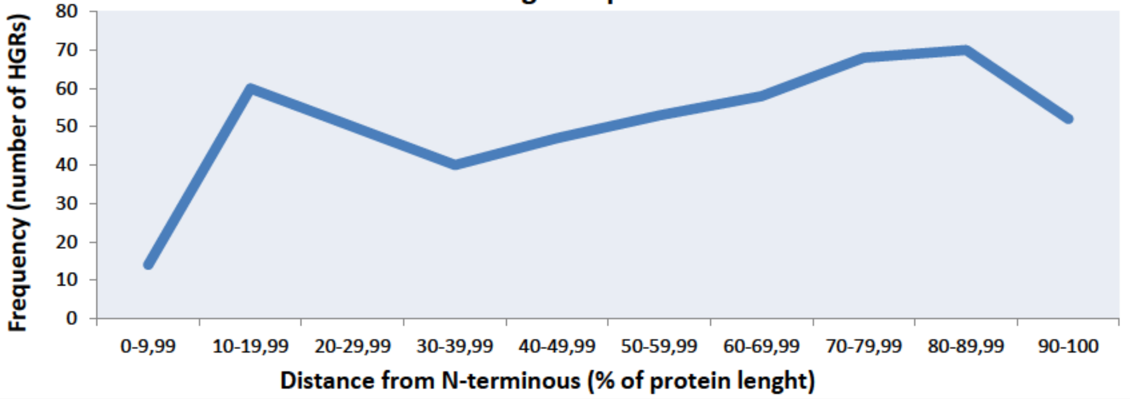

5. Frequency distribution of HGR lengths

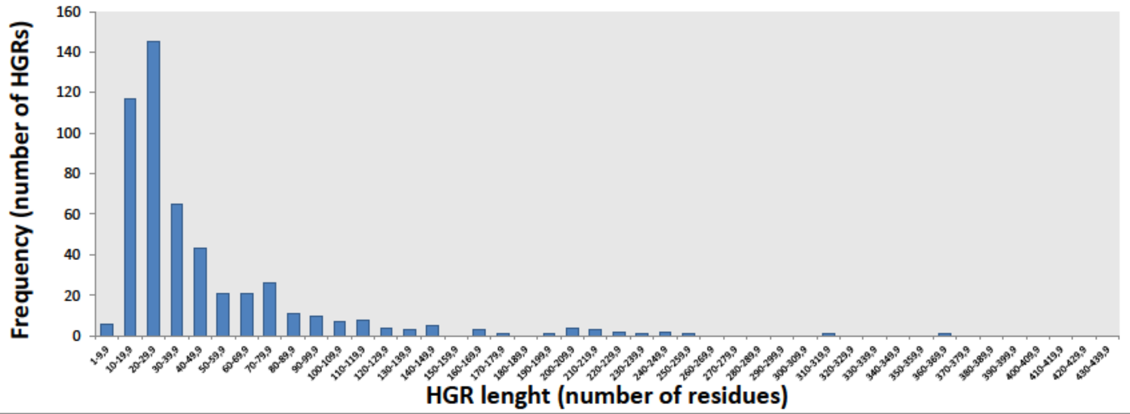

6. Alphabetical list of proteins with HGRs. Start and end positions of every HGR found. (Protein name: start-end. start-end. start-end. …)

SS1T_00074 191-201. 399-419.
SS1T_00095 71-98.
SS1T_00173 282-302.
SS1T_00233 474-621.
SS1T_00265 457-541.
SS1T_00274 324-341.
SS1T_00311 126-143.
SS1T_00376 513-539.
SS1T_00380 176-196.
SS1T_00395 403-426.
SS1T_00458 34-70.
SS1T_00474 201-219.
SS1T_00509 47-60.
SS1T_00534 30-51. 66-107.
SS1T_00561 44-111.
SS1T_00642 412-461. 508-522.
SS1T_00744 45-69.
SS1T_00746 61-95.
SS1T_00772 439-468.
SS1T_00781 156-191. 297-314. 512-524.
SS1T_00809 166-270.
SS1T_00872 112-209.
SS1T_00877 69-85.
SS1T_00891 58-86.
SS1T_00892 64-92.
SS1T_00946 143-164.
SS1T_01027 98-117. 300-312. 510-521. 753-768.
SS1T_01083 565-587.
SS1T_01107 33-75.
SS1T_01109 236-255.
SS1T_01176 152-200. 352-388.
SS1T_01200 72-88.
SS1T_01212 118-138.
SS1T_01235 20-34.
SS1T_01262 73-169.
SS1T_01297 27-48.
SS1T_01298 362-371.
SS1T_01329 255-269. 293-335.
SS1T_01334 753-776.
SS1T_01373 62-103.
SS1T_01427 245-256.
SS1T_01428 48-82. 185-204.
SS1T_01618 62-95.
SS1T_01749 36-52. 148-212. 270-366.
SS1T_01828 243-292.
SS1T_01838 245-269.
SS1T_01851 46-79. 105-218.
SS1T_01866 210-239.
SS1T_01901 78-101. 210-237.
SS1T_01966 27-85.
SS1T_02014 224-356.
SS1T_02126 40-62.
SS1T_02131 33-64. 116-225. 280-309. 409-465.
SS1T_02155 148-226.
SS1T_02247 64-75.
SS1T_02250 61-72. 116-156. 199-222.
SS1T_02268 243-273.
SS1T_02334 442-469.
SS1T_02369 243-320.
SS1T_02370 430-477.
SS1T_02399 439-499.
SS1T_02412 198-246.
SS1T_02501 338-411.
SS1T_02522 125-143.
SS1T_02542 137-159.
SS1T_02570 412-435.
SS1T_02580 90-156.
SS1T_02587 117-205.
SS1T_02599 23-168.
SS1T_02600 43-82. 121-160. 174-237. 265-316. 336-380.
SS1T_02655 129-155. 199-234.
SS1T_02714 180-380.
SS1T_02760 31-53.
SS1T_02804 65-93.
SS1T_02812 453-490.
SS1T_02845 211-224.
SS1T_02865 159-176.
SS1T_02872 324-342. 387-420. 509-524. 839-857. 904-928. 1090-1121. 1203-1219. 1244-1271. 1339-1356.
SS1T_02920 193-281.
SS1T_03000 27-50.
SS1T_03076 25-102. 266-286.
SS1T_03160 458-485.
SS1T_03214 78-93. 120-135. 303-418.
SS1T_03229 263-362.
SS1T_03230 123-188.
SS1T_03268 222-241. 297-316. 374-518. 606-805. 900-958.
SS1T_03276 78-105.
SS1T_03326 64-83. 95-199.
SS1T_03364 563-580.
SS1T_03378 42-250.
SS1T_03387 64-104.
SS1T_03395 40-72. 204-230.
SS1T_03442 113-126.
SS1T_03535 22-37.
SS1T_03576 237-251.
SS1T_03618 218-246.
SS1T_03629 87-106.
SS1T_03650 54-80.
SS1T_03653 243-308.
SS1T_03656 19-35.
SS1T_03709 23-41. 110-163.
SS1T_03740 26-54.
SS1T_03744 52-85.
SS1T_03795 115-132.
SS1T_03803 51-263.
SS1T_03900 406-423.
SS1T_03941 356-370. 445-537.
SS1T_04008 630-644.
SS1T_04085 229-304.
SS1T_04095 177-190.
SS1T_04158 268-277.
SS1T_04171 26-85.
SS1T_04213 512-526. 777-858. 976-1025. 1131-1143. 1223-1244. 1325-1397. 1677-1738.
SS1T_04247 38-65. 108-131. 155-174.
SS1T_04264 147-172. 226-256. 318-344. 395-421. 521-764. 864-907.
SS1T_04312 71-88. 132-230. 306-512. 568-608. 695-728. 811 846. 880-1047. 1063-1150.
SS1T_04343 477-568.
SS1T_04393 36-101.
SS1T_04425 130-257.
SS1T_04429 19-35. 128-169.
SS1T_04497 281-356.
SS1T_04515 437-446.
SS1T_04519 25-36.
SS1T_04592 61-76.
SS1T_04611 29-44.
SS1T_04639 318-338.
SS1T_04683 175-211.
SS1T_04685 36-57.
SS1T_04786 222-326.
SS1T_04850 413-436.
SS1T_04852 303-438.
SS1T_04857 18-38.
SS1T_04871 27-40.
SS1T_04874 64-73.
SS1T_04875 32-53.
SS1T_04891 150-177.
SS1T_04898 190-225. 411-432.
SS1T_04923 44-58.
SS1T_04945 405-422. 459-549.
SS1T_04946 270-293. 334-378. 459-500.
SS1T_05013 80-108.
SS1T_05037 27-45.
SS1T_05075 96-199.
SS1T_05110 401-420.
SS1T_05278 58-91. 129-169. 226-247. 276-443.
SS1T_05299 64-90. 138-213.
SS1T_05325 77-113.
SS1T_05337 22-38.
SS1T_05457 160-197.
SS1T_05460 409-418.
SS1T_05494 977-1037.
SS1T_05592 104-133.
SS1T_05609 28-47.
SS1T_05659 108-122. 154-176.
SS1T_05668 81-116.
SS1T_05784 138-167.
SS1T_05989 23-45.
SS1T_06009 326-382.
SS1T_06027 638-703.
SS1T_06075 64-79.
SS1T_06100 30-70.
SS1T_06119 26-43.
SS1T_06235 413-443.
SS1T_06264 766-794.
SS1T_06269 36-75.
SS1T_06293 129-164.
SS1T_06297 162-225.
SS1T_06365 394-411.
SS1T_06513 188-221.
SS1T_06542 390-412.
SS1T_06590 36-107. 204-227.
SS1T_06695 311-336.
SS1T_06713 70-156.
SS1T_06742 51-80.
SS1T_06747 29-129.
SS1T_06781 107-131. 263-283.
SS1T_06817 52-262.
SS1T_06862 66-96.
SS1T_06890 116-130. 232-253.
SS1T_06942 28-38. 196-228. 359-417. 453 527.
SS1T_06988 27-33.
SS1T_07042 189-223.
SS1T_07072 290-311.
SS1T_07224 64-82.
SS1T_07234 123-196.
SS1T_07295 82-199.
SS1T_07359 40-70. 96-173. 544-566.
SS1T_07416 29-342.
SS1T_07426 636-652. 692-714.
SS1T_07554 37-228.
SS1T_07639 25-38.
SS1T_07655 394-416.
SS1T_07656 244-293.
SS1T_07667 66-241. 274-420. 440-493.
SS1T_07723 51-69. 146-231.
SS1T_07725 97-116.
SS1T_07758 23-60.
SS1T_07784 25-47. 184-210.
SS1T_07836 67-86. 146-163.
SS1T_07844 157-187.
SS1T_07847 756-800.
SS1T_07863 238-263.
SS1T_07928 140-160.
SS1T_08104 237-264.
SS1T_08135 508-529.
SS1T_08160 26-33.
SS1T_08229 387-409.
SS1T_08263 115-143. 162-273. 327-359. 385-472. 552-567. 620-657. 897-916.
SS1T_08292 91-103.
SS1T_08528 268-310.
SS1T_08529 49-84.
SS1T_08542 28-42.
SS1T_08566 22-45.
SS1T_08587 200-238. 439-455.
SS1T_08695 319-372.
SS1T_08698 154-232.
SS1T_08786 295-323. 421-435.
SS1T_08834 31-63.
SS1T_08892 104-120.
SS1T_08907 316-433.
SS1T_08917 74-95. 137-166. 180-231.
SS1T_09000 492-517. 895-915.
SS1T_09020 415-420.
SS1T_09045 128-146.
SS1T_09050 335-381.
SS1T_09067 278-316.
SS1T_09105 378-418.
SS1T_09169 486-545. 718-790.
SS1T_09215 30-56.
SS1T_09251 238-272.
SS1T_09256 44-54.
SS1T_09278 123-146.
SS1T_09299 225-237. 361-385.
SS1T_09371 152-167.
SS1T_09380 826-843.
SS1T_09381 46-209.
SS1T_09392 264-295.
SS1T_09578 150-200.
SS1T_09630 23-39.
SS1T_09768 19-58.
SS1T_09795 283-305.
SS1T_09844 98-140.
SS1T_09861 456-535.
SS1T_09982 170-219.
SS1T_10087 621-639.
SS1T_10115 75-105.
SS1T_10151 667-679.
SS1T_10161 260-332.
SS1T_10229 105-125. 206-227.
SS1T_10237 37-61.
SS1T_10260 1041-1086.
SS1T_10266 128-379.
SS1T_10301 322-339.
SS1T_10306 388-408.
SS1T_10353 397-424.
SS1T_10434 31-69.
SS1T_10454 141-154.
SS1T_10572 358-375.
SS1T_10698 80-141.
SS1T_10707 314-342.
SS1T_10728 92-334. 397-413. 447-472. 513-551.
SS1T_10746 70-89. 123-170.
SS1T_10768 396-414.
SS1T_10827 81-148.
SS1T_10836 51-148.
SS1T_10845 72-86. 219-246.
SS1T_10923 79-94. 150-365. 408-426.
SS1T_10936 95-316.
SS1T_11065 25-41.
SS1T_11120 33-67. 185-325.
SS1T_11202 61-138. 187-284. 305-414. 455-517. 541-586.
SS1T_11216 222-244.
SS1T_11223 149-197.
SS1T_11236 304-326.
SS1T_11382 605-635.
SS1T_11413 93-140. 162-237.
SS1T_11468 109-137.
SS1T_11499 85-100.
SS1T_11579 388-408.
SS1T_11600 298-316. 452-475. 569-586.
SS1T_11673 24-48.
SS1T_11837 387-406.
SS1T_11863 125-130.
SS1T_11910 49-84. 150-191. 225-240. 385-404. 441-496. 705-926.
SS1T_11927 33-84.
SS1T_11929 139-210. 311-357.
SS1T_11977 324-391.
SS1T_12017 477-503.
SS1T_12024 77-197.
SS1T_12043 291-311.
SS1T_12052 126-138.
SS1T_12059 226-304.
SS1T_12200 524-553.
SS1T_12262 92-138.
SS1T_12263 212-242.
SS1T_12291 26-39. 148-207.
SS1T_12294 336-411.
SS1T_12336 193-291.
SS1T_12482 159-184.
SS1T_12509 188-202. 317-338.
SS1T_12513 106-134. 196-221. 281-311.
SS1T_12619 281-305.

SS1T 12741	480-504.
SS1T_12765	407-430.
SS1T_12771	108-128.
SS1T 12889	20-97.
SS1T 12910	452-478.
SS1T 12938	192-552.
SS1T_12963	169-232.
SS1T 12969	477-496.
SS1T_12999	255-334. 424-462.
SS1T 13012	135-165.
SS1T 13035	64-105.
SS1T 13036	115-185.
SS1T 13039	444-469.
SS1T_13046	681-721.
SS1T_13091	59-72.
SS1T 13115	185-255.
SS1T 13142	73-111.
SS1T 13199	296-312.
SS1T 13214	201-241.
SS1T_13277	22-61. 143-167. 298-323.
SS1T_13279	66-88.
SS1T_13465	44-51.
SS1T 13472	507-528.
SS1T 13578	30-131.
SS1T 13589	51-68. 199-216.
SS1T 13599	102-146.
SS1T_13682	113-138. 157-185. 198-256. 285-327. 470-493. 525-543. 581-635. 665-677.
SS1T_13732	71-91. 138-155. 204-223. 270-289. 336-382.
SS1T 13736	37-51. 81-119.
SS1T 13771	47-90.
SS1T 13777	314-343.
SS1T_13860	245-333.
SS1T 13935	113-185. 255-489. 514-529.
SS1T_13989	294-312.
SS1T 14007	31-77.
SS1T 14011	52-81.
SS1T 14041	45-63.
SS1T 14153	23-27. 1055-1075.
SS1T_14158	245-315.
SS1T_14160	248-364.
SS1T 14163	459-482.
SS1T 14180	35-57.
SS1T 14184	179-191.
SS1T 14220	268-284.
SS1T 14307	98-224.
SS1T_14320	22-55.
SS1T_14343	163-175.
SS1T_14408	76-213. 257-270. 320-375.
SS1T 14460	347-360. 401-430.
SS1T 14492	44-57.
SS1T 14495	67-90.
SS1T_14497	403-530. 590-654.
SS1T_14515	46-64.

224-245. 325-348.

7. Graphical representation of all HGRs found in the protein set

Proteins are represented as boxes of lenght proportional to protein length. Black regions indicate HGRs

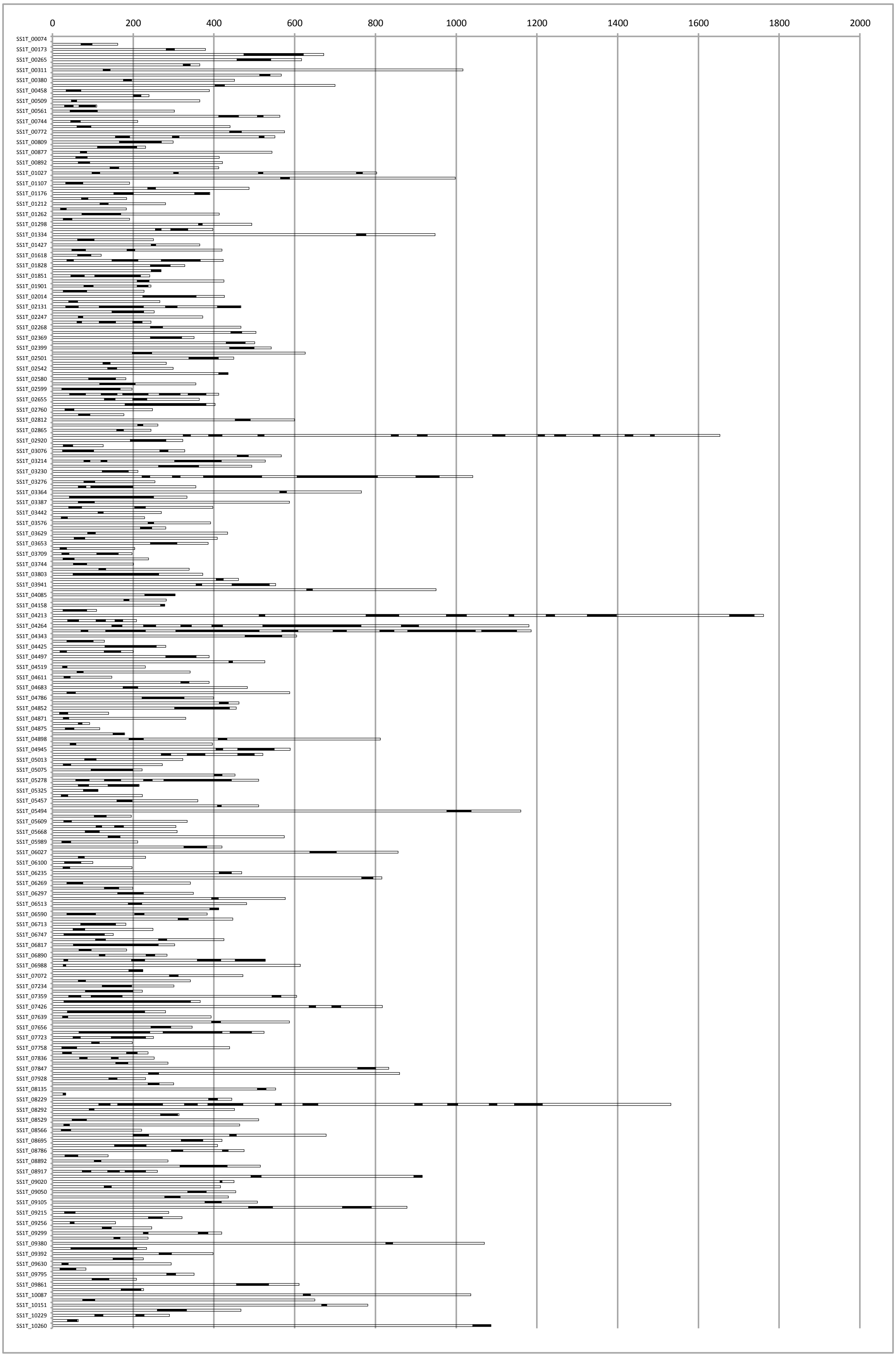

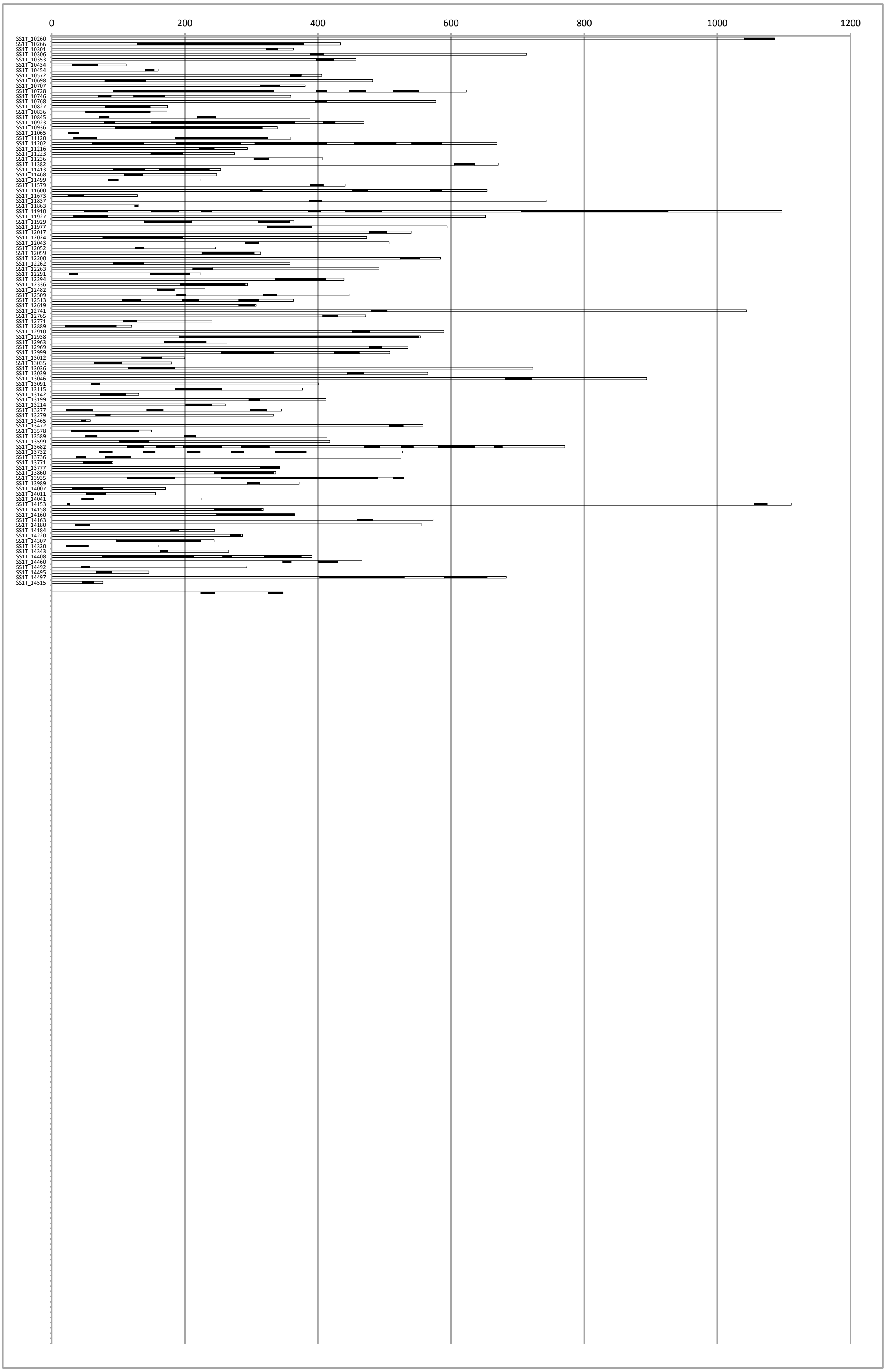
0
200
400
600
800
1000
1200
SS1T_10260
SS1T_10266
SS1T_10301
SS1T_10306
SS1T_10353
SS1T_10434
SS1T_10454
SS1T_10572
SS1T_10698
SS1T_10707
SS1T_10728
SS1T_10746
SS1T_10768
SS1T_10827
SS1T_10836
SS1T_10845
SS1T_10923
SS1T_10936
SS1T_11065
SS1T_11120
SS1T_11202
SS1T_11216
SS1T_11223
SS1T_11236
SS1T_11382
SS1T_11413
SS1T_11468
SS1T_11499
SS1T_11579
SS1T_11600
SS1T_11673
SS1T_11837
SS1T_11863
SS1T_11910
SS1T_11927
SS1T_11929
SS1T_11977
SS1T_12017
SS1T_12024
SS1T_12043
SS1T_12052
SS1T_12059
SS1T_12200
SS1T_12262
SS1T_12263
SS1T_12291
SS1T_12294
SS1T_12336
SS1T_12482
SS1T_12509
SS1T_12513
SS1T_12619
SS1T_12741
SS1T_12765
SS1T_12771
SS1T_12889
SS1T_12910
SS1T_12938
SS1T_12963
SS1T_12969
SS1T_12999
SS1T_13012
SS1T_13035
SS1T_13036
SS1T_13039
SS1T_13046
SS1T_13091
SS1T_13115
SS1T_13142
SS1T_13199
SS1T_13214
SS1T_13277
SS1T_13279
SS1T_13465
SS1T_13472
SS1T_13578
SS1T_13589
SS1T_13599
SS1T_13682
SS1T_13732
SS1T_13736
SS1T_13771
SS1T_13777
SS1T_13860
SS1T_13935
SS1T_13989
SS1T_14007
SS1T_14011
SS1T_14041
SS1T_14153
SS1T_14158
SS1T_14160
SS1T_14163
SS1T_14180
SS1T_14184
SS1T_14220
SS1T_14307
SS1T_14320
SS1T_14343
SS1T_14408
SS1T_14460
SS1T_14492
SS1T_14495
SS1T_14497
SS1T_14515

HGR_detection V1.1 Report.	*T. reesei*

1. Parameters used in HGR detection			
**Glycosylation Density (%)**	**Window**	**Limit**	**Separator**
25	20	5	5

2. Input protein set			
Number of proteins	695	Largest protein	3204 aa
Protein length average	454,47	Smallest protein	54 aa

3. HGRs found in the protein set			
No. of proteins with HGRs	233	Number of HGRs	311
Average HGR length	52,17	Maximum HGR length	418
Average position of HGR centers (as % of protein lenght)	58,0	Minimum HGR length	7

4. Frequency distribution of the positions of HGR centers along the length of proteins

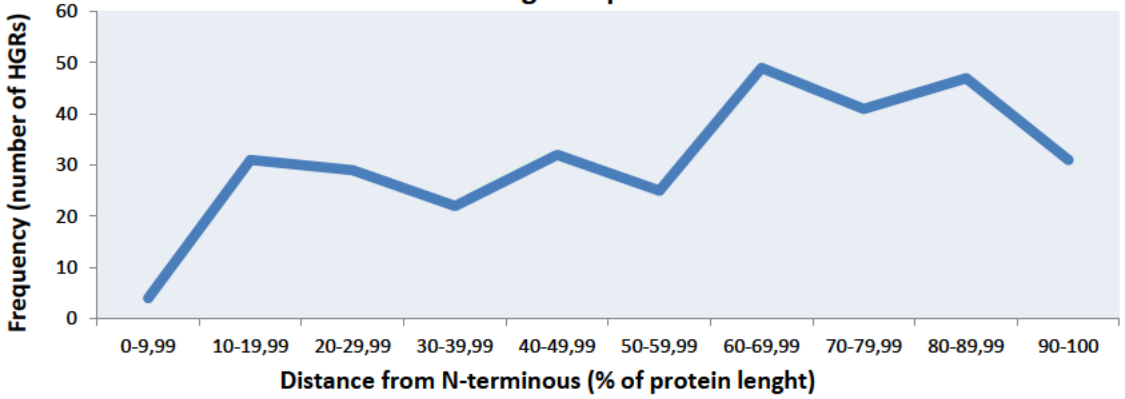

5. Frequency distribution of HGR lengths

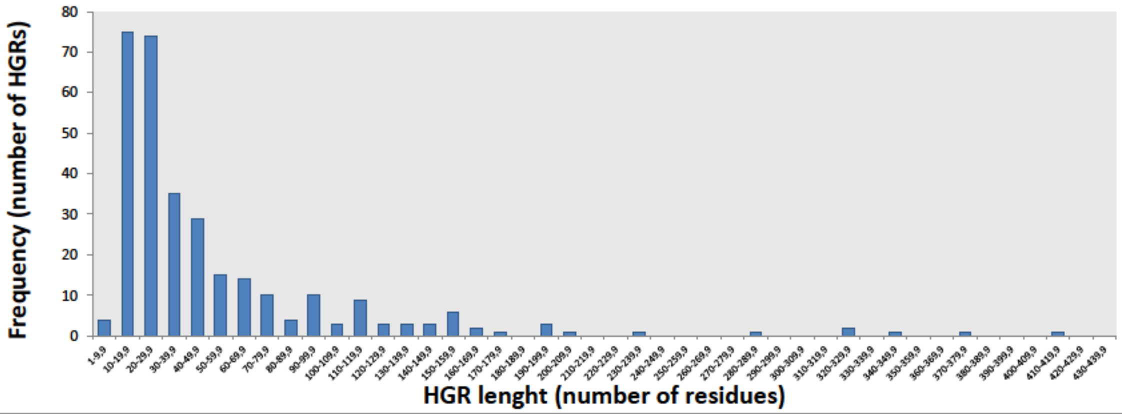

6. Alphabetical list of proteins with HGRs. Start and end positions of every HGR found.
(Protein name: start-end. start-end. start-end. …)

jg |T e2|102437| 305-631.
jg |T e2|102582| 271-308.
jg |T e2|102617| 307-361.
jg |T e2|102836| 95-121.
jg |T e2|102837| 106-275.
jg |T e2|102850| 34-56. 250-265.
jg |T e2 102851| 40-52.
jg |T e2|102908| 25-41.
jg |T e2|102938| 65-91. 135-173.
jg |T e2|103162| 73-103. 226-245.
jg |T e2 103236| 356-402.
jg |T e2|103393| 78-101.
jg |T e2|103653| 40-82.
jg |T e2|103712| 165-192.
jg |T e2|103740| 100-149.
jg |T e2|103866| 77-143.
jg |T e2|104277| 31-48.
jg |T e2 104394| 30-41.
jg |T e2|104401| 98-171.
jg |T e2|104664| 84-159.
jg |T e2|104925| 421-452. 525-554.
jg |T e2|105291| 347-366.
jg |T e2|105313| 393-543. 1199-1210. 1378-1413. 1599-1625.
jg |T e2|105336| 105-111. 309-342.
jg |T e2 105444| 200-210.
jg |T e2|105483| 29-104.
jg |T e2|105553| 23-54.
jg |T e2|105589| 20-37.
jg |T e2|105709| 143-232.
jg |T e2|105763| 97-189.
jg |T e2|105840| 362-380.
jg |T e2 105860| 284-326.
jg |T e2|105984| 142-231.
jg |T e2|106024| 126-277.
jg |T e2|106138| 162-175. 247-390. 419-511. 674-701.
jg |T e2 106223| 111-302.
jg |T e2|106353| 173-215.
jg |T e2|106615| 33-61.
jg |T e2|106998| 80-196.
jg |T e2|107011| 49-79. 129-152.
jg |T e2|107436| 26-107.
jg |T e2|107495| 69-85.
jg |T e2 107523| 437-457.
jg |T e2|107644| 119-139.
jg |T e2|107856| 154-165.
jg |T e2|108233| 125-140.
jg |T e2|108387| 92-99. 185-207.
jg |T e2|108418| 94-111.
jg |T e2|108546| 32-60. 107-160. 207-237.
jg |T e2 108570| 134-198.
jg |T e2|108642| 316-341. 355-374. 454-469.
jg |T e2|108672| 492-521.
jg |T e2|109235| 227-322.
jg |T e2|109364| 131-146. 324-359.
jg |T e2|109385| 201-214.
jg |T e2|109828| 25-56.
jg |T e2|109907| 36-59.
jg |T e2|109934| 57-249. 438-784.
jg |T e2|109994| 550-565.
jg |T e2|110140| 70-168.
jg |T e2 110317| 333-357.
jg |T e2|110711| 63-144. 220-278.
jg |T e2|110862| 63-116. 176-234.
jg |T e2|110943| 124-150. 165-231. 715-724. 1044-1059. 1140 1156.
jg |T e2|110949| 108-142.
jg |T e2|111272| 230-250.
jg |T e2|111341| 241-256. 301-328.
jg |T e2 111450| 290-308.
jg |T e2|111495| 63-92.
jg |T e2|111632| 86-108. 155-178.
jg |T e2|111799| 60-79.
jg |T e2|111803| 151-169.
jg |T e2|111951| 50-107. 141-256.
jg |T e2|111955| 148-213.
jg |T e2 112002| 29-49.
jg |T e2|112018| 36-47.
jg |T e2|112258| 21-41. 102-124.
jg |T e2|112458| 219-246.
jg |T e2|112681| 53-121.
jg |T e2|119552| 142-294.
jg |T e2|119568| 106-218.
jg |T e2|119805| 105-196.
jg |T e2|119902| 227-248.
jg |T e2|119963| 93-161.
jg |T e2|119975| 35-125.
jg |T e2 120067| 91-111.
jg |T e2|120189| 153-215.
jg |T e2|120294| 199-209.
jg |T e2|120312| 61-89.
jg |T e2|120320| 156-170.
jg |T e2|120351| 204-275. 387-405.
jg |T e2|120453| 80-107.
jg |T e2 120697| 65-86. 153-165.
jg |T e2|120821| 129-243.
jg |T e2|120823| 62-110. 135-459. 494-654. 685-835.
jg |T e2|120837| 205-237.
jg |T e2|120873| 492-534.
jg |T e2|120937| 239-256. 661-679.
jg |T e2|120980| 220-338.
jg |T e2 121135| 74-100.
jg |T e2|121251| 246-529. 550-595. 681-749.
jg |T e2|121306| 414-553. 634-678. 702-722.
jg |T e2|121475| 505-519.
jg |T e2 121689| 55-74. 92-231. 328-424. 458-596. 698-849. 890-902.
jg |T e2|121702| 173-194. 391-413.
jg |T e2|121818| 120-149. 179-260. 275-361.
jg |T e2|122047| 66-178. 252-268. 293-497.
jg |T e2|122081| 392-418.
jg |T e2|122198| 85-123.
jg |T e2|122242| 203-230. 331-357.
jg |T e2 122255| 230-284. 311-338.
jg |T e2|122455| 188-262.
jg |T e2|122506| 34-48. 105-123. 243-259. 318-510. 652-671. 700-777.
jg |T e2|122657| 112-162. 199-236.
jg |T e2|122870| 94-198.
jg |T e2|122874| 199-273.
jg |T e2|122941| 147-195. 288-331.
jg |T e2 123039| 91-220.
jg |T e2|123047| 52-206.
jg |T e2|123191| 20-143.
jg |T e2|123199| 61-81.
jg |T e2|123213| 53-216. 269-288. 341-368. 390-621. 636-657.
jg |T e2|123327| 90-130.
jg |T e2|123475| 107-524. 603-713.
jg |T e2 123538| 399-424.
jg |T e2|123539| 112-152.
jg |T e2|123561| 658-693.
jg |T e2|123608| 84-100.
jg |T e2 123614| 20-91.
jg |T e2|123636| 106-215.
jg |T e2|123650| 29-159.
jg |T e2|123659| 196-247. 257-301.
jg |T e2|123680| 119-172.
jg |T e2|123776| 32-141. 202-274. 402-413.
jg |T e2|123777| 56-71.
jg |T e2 123911| 260-307.
jg |T e2|123940| 65-95.
jg |T e2|123976| 69-165. 235-297.
jg |T e2|123989| 406-418. 446-479.
jg |T e2|123992| 68-113.
jg |T e2|124043| 348-376.
jg |T e2|124059| 58-77. 121-139.
jg |T e2 124083| 128-145.
jg |T e2|124173| 44-76.
jg |T e2|124277| 20-37. 85-120.
jg |T e2|124282| 364-377.
jg |T e2|124295| 103-122.
jg |T e2|1885| 481-528.
jg |T e2|21342| 533-558.
jg |T e2|21392| 161-173.
jg |T e2|21412| 85-191.
g |T e2|21415| 456-470.
g |T e2|21468| 134-256.
g |T e2|21576| 39-55.
g |T e2|22129| 248-314.
g |T e2|22181| 413-456.
g |T e2|22564| 138-184.
g |T e2|23115| 68-193.
g |T e2|27181| 365-396.
g |T e2|28928| 281-298.
g |T e2|32293| 64-77.
g |T e2|3488| 54-72.
g |T e2|35465| 614-630.
g |T e2|37271| 228-250. 374-390.
g |T e2|37665| 453-494.
g |T e2|3787| 253-272.
g |T e2|3824| 144-156.
g |T e2|41035| 235-253.
g |T e2|4240| 27-85.
g |T e2|44214| 227-271.
g |T e2|44230| 182-214.
g |T e2|49081| 754-792.
g |T e2|49193| 51-155. 227-267.
g |T e2|49976| 174-208.
g |T e2|50625| 504-510.
g |T e2|50647| 683-747.
g |T e2|51946| 39-49.
g |T e2|53777| 348-366.
g |T e2|53947| 115-177.
g |T e2|54426| 68-83.
g |T e2|54723| 104-130. 191-212.
g |T e2|54761| 21-36.
g |T e2|54846| 877-902.
g |T e2|54858| 273-297.
g |T e2|56341| 441-450.
g |T e2|56512| 118-129.
g |T e2|56531| 267-289.
g |T e2|58239| 297-318.
g |T e2|60456| 59-72.
g |T e2|61382| 173-185.
g |T e2|61526| 127-140.
g |T e2|61584| 25-41.
g |T e2|61830| 58-78.
g |T e2|62333| 145-166.
g |T e2|63416| 153-177.
g |T e2|63558| 61-101.
g |T e2|64044| 99-115.
g |T e2|64181| 162-180.
g |T e2|64261| 489-532.
g |T e2|64448| 142-152. 358-403.
g |T e2|66616| 613-640.
g |T e2|66792| 308-328.
g |T e2|68347| 334-366.
g |T e2|69061| 322-366.
g |T e2|69276| 417-459.
g |T e2|70092| 270-288.
g |T e2|71441| 36-97. 318-335.
g |T e2|72072| 208-221.
g |T e2|72339| 982-1007. 1055-1087. 1408-1431.
g |T e2|72567| 71-111.
g |T e2|72800| 307-327.
g |T e2|72907| 288-663. 768-802.
g |T e2|73519| 264-278.
g |T e2|73632| 250-269.
g |T e2|73638| 240-283.
g |T e2|73643| 251-310.
g |T e2|73897| 149-158.
g |T e2|74060| 51-74.
g |T e2|76065| 243-296.
g |T e2|76075| 93-105.
g |T e2|76117| 422-436.
g |T e2|76266| 354-390.
g |T e2|76659| 180-200.
g |T e2|77577| 156-190.
g |T e2|78264| 17-38.
g |T e2|79448| 123-164.
g |T e2|79779| 1144-1181.
g |T e2|79807| 427-485.
g |T e2|81296| 73-105.
g |T e2|81331| 30-106.
g |T e2|81517| 997-1018.
g |T e2|82616| 354-404.
587-594.

7. Graphical representation of all HGRs found in the protein set

Proteins are represented as boxes of lenght proportional to protein length. Black regions indicate HGRs

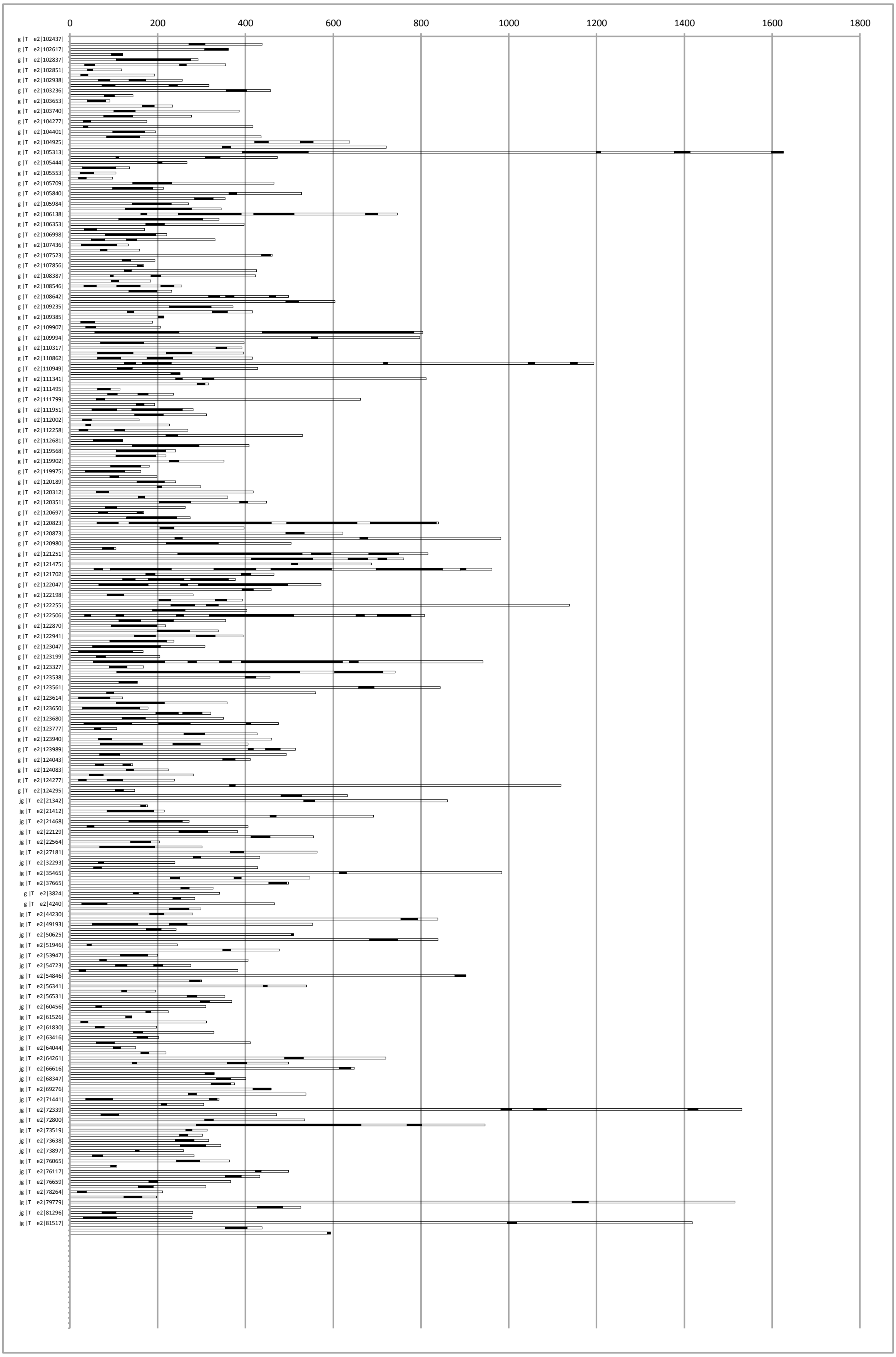

HGR_detection V1.1 Report.	*U. maydis*

1. Parameters used in HGR detection			
**Glycosylation Density (%)**	**Window**	**Limit**	**Separator**
25	20	5	5

2. Input protein set			
Number of proteins	603	Largest protein	4044 aa
Protein length average	491,36	Smallest protein	55 aa

3. HGRs found in the protein set			
No. of proteins with HGRs	214	Number of HGRs	276
Average HGR length	32,31	Maximum HGR length	145
Average position of HGR centers (as % of protein lenght)	53,5	Minimum HGR length	5

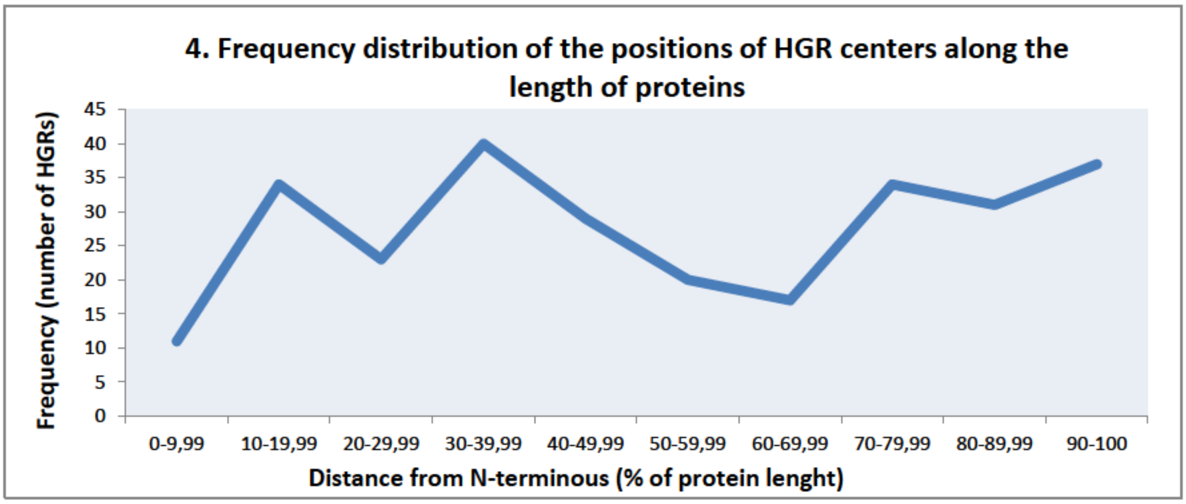

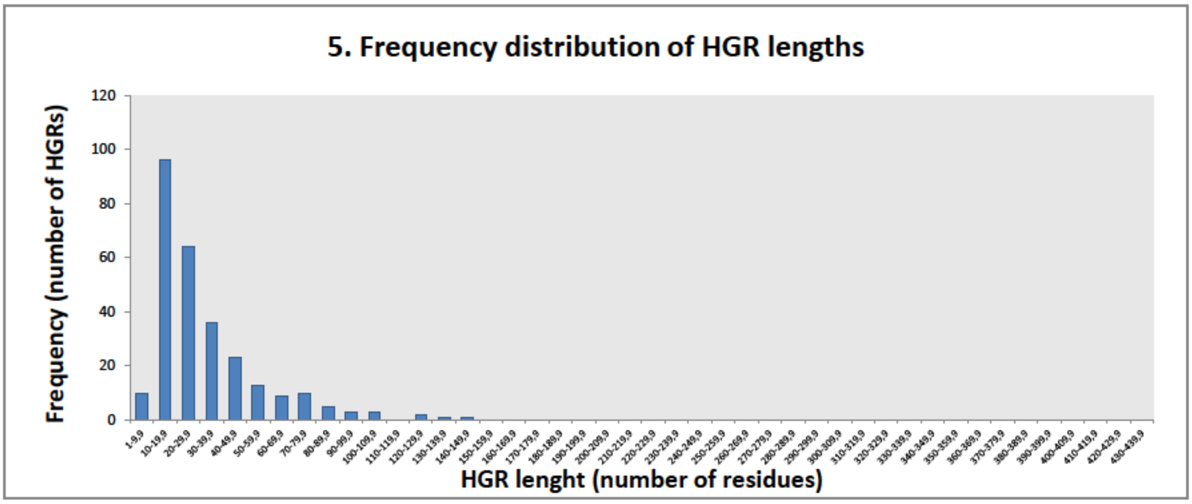

6. Alphabetical list of proteins with HGRs. Start and end positions of every HGR found. (Protein name: start-end. start-end. start-end. …)

UM00013 247-261.
UM00042 363-392.
UM00063 151-204. 479-498.
UM00070 611-651.
UM00072 423-465.
UM00081 166-240.
UM00104 251-266.
UM00200 34-68. 95-119. 926-941.
UM00250 208-243.
UM00265 133-169. 241-262.
UM00299 85-102. 672-690.
UM00309 302-395.
UM00326 234-276.
UM00327 191-214.
UM00365 84-104.
UM00480 132-203. 370-469. 518-538. 587 607.
UM00534 39-55. 244-282.
UM00538 291-307.
UM00542 327-345.
UM00567 173-253.
UM00634 33-45.
UM00638 346-398.
UM00662 123-140.
UM00692 33-54. 161-245.
UM00698 55-86. 345-360.
UM00715 156-172.
UM00723 44-86. 286-300. 375-467.
UM00730 92-149.
UM00799 230-244.
UM00834 76-183.
UM00860 287-317. 357-384.
UM00873 121-193. 550-562.
UM00912 58-75.
UM00967 122-206.
UM01082 45-145.
UM01143 417-458.
UM01204 91-127.
UM01210 210-331.
UM01213 154-193.
UM01240 98-113.
UM01244 501-514. 594-602.
UM01289 1066-1092. 1102-1119.
UM01334 238-252. 308-329.
UM01365 75-93.
UM01377 150-171.
UM01499 105-160.
UM01513 114-131. 172-191. 226-356. 468-483. 575-623.
UM01542 110-164.
UM01547 964-978.
UM01553 38-59.
UM01598 316-331.
UM01640 515-545.
UM01653 58-70.
UM01666 182-217.
UM01689 58-80. 243-264.
UM01725 124-149.
UM01734 132-147.
UM01808 113-135. 268-286.
UM01823 25-64. 93-107.
UM01844 76-113. 135-154.
UM01851 150-203.
UM01895 132-184.
UM01940 165-178. 213-220.
UM01949 225-240.
UM01970 56-75.
UM01972 169-185.
UM01976 80-120.
UM01985 143-177.
UM02011 385-398.
UM02019 50-84.
UM02063 147-175. 292-325.
UM02080 29-81.
UM02087 177-199.
UM02093 481-497.
UM02138 80-86. 223-241.
UM02194 94-107.
UM02212 43-99.
UM02254 31-49. 172-191. 229-248.
UM02261 111-153. 179-227.
UM02294 86-109.
UM02295 118-140.
UM02298 114-139.
UM02299 423-444.
UM02316 584-618.
UM02381 491-515.
UM02430 235-273.
UM02465 41-52.
UM02526 27-37.
UM02539 196-201.
UM02640 248-284.
UM02652 95-155.
UM02738 84-204.
UM02767 162-225.
UM02770 702-716.
UM02813 57-74.
UM02925 103-127. 179-213. 321-332. 392-470.
UM02928 54-94. 120-188.
UM02940 89-125.
UM02981 28-41.
UM03043 72-115.
UM03109 281-318.
UM03110 32-43.
UM03139 215-242.
UM03174 149-182. 336-345. 879-894. 1097-1107.
UM03236 38-58.
UM03327 57-72.
UM03349 245-312.
UM03367 1283-1295.
UM03381 289-300.
UM03383 53-67.
UM03403 1153-1175.
UM03408 428-447.
UM03422 463-480.
UM03467 121-157.
UM03476 253-269.
UM03487 115-131. 371-382.
UM03510 469-502. 588-600.
UM03532 201-216.
UM03564 32-59. 174-192.
UM03574 126-157. 319-330. 351-369. 448-477.
UM03586 87-101.
UM03587 37-51. 172-176.
UM03614 429-461.
UM03616 598-613.
UM03634 106-165.
UM03660 505-593.
UM03707 220-245.
UM03776 582-660.
UM03812 29-42. 618-632.
UM03818 88-106.
UM03822 24-58.
UM03880 338-356.
UM03900 309-342.
UM03924 480-516. 540-560.
UM03977 104-193.
UM03999 31-47.
UM04033 25-51. 87-113.
UM04084 95-180.
UM04166 154-177.
UM04185 194-253.
UM04259 318-338.
UM04267 665-684.
UM04282 29-48.
UM04297 327-377.
UM04368 185-329. 390-456.
UM04395 85-114.
UM04403 1398-1412.
UM04422 463-479.
UM04525 42-57. 160-173.
UM04528 46-116.
UM04601 261-308.
UM04627 216-243.
UM04637 328-344.
UM04639 31-48.
UM04649 343-365.
UM04650 38-52.
UM04656 280-303. 341 356.
UM04698 267-287.
UM04807 410-442.
UM04816 336-353.
UM04818 29-37. 59-78.
UM04893 103-117.
UM04994 251-273.
UM05036 111-179.
UM05072 57-71.
UM05154 55-70.
UM05167 172-191.
UM05220 141-155.
UM05305 60-75.
UM05331 145-162.
UM05349 212-225.
UM05352 341-367.
UM05361 1160-1190. 1250-1269.
UM05366 181-204.
UM05419 361-390.
UM05430 257-264.
UM05439 212-270.
UM05495 233-278.
UM05546 26-51.
UM05604 140-157.
UM05615 94-148.
UM05622 121-169.
UM05631 202-216. 1009-1029.
UM05684 280-307.
UM05696 49-89.
UM05704 60-107.
UM05741 32-105.
UM05819 53-68.
UM05824 67-84. 156-175.
UM05953 121-152.
UM05995 32-74.
UM06064 57-78. 97-120.
UM06119 162-209.
UM06127 259-272.
UM06128 283-291.
UM06141 65-143.
UM06158 102-120.
UM06162 274-349.
UM06179 280-295.
UM06181 72-80.
UM06190 121-156.
UM06216 197-225.
UM06218 119-155.
UM06221 149-197.
UM06223 97-116.
UM06255 230-258. 307 364. 411-430. 493-533. 589-667.
UM06332 327-366.
UM06357 254-280.
UM06365 304-317.
UM06370 334-340.
UM06431 104-116. 190 207.
UM06434 565-582.
UM06440 167-230.
UM06467 348-362.

7. Graphical representation of all HGRs found in the protein set

Proteins are represented as boxes of lenght proportional to protein length. Black regions indicate HGRs

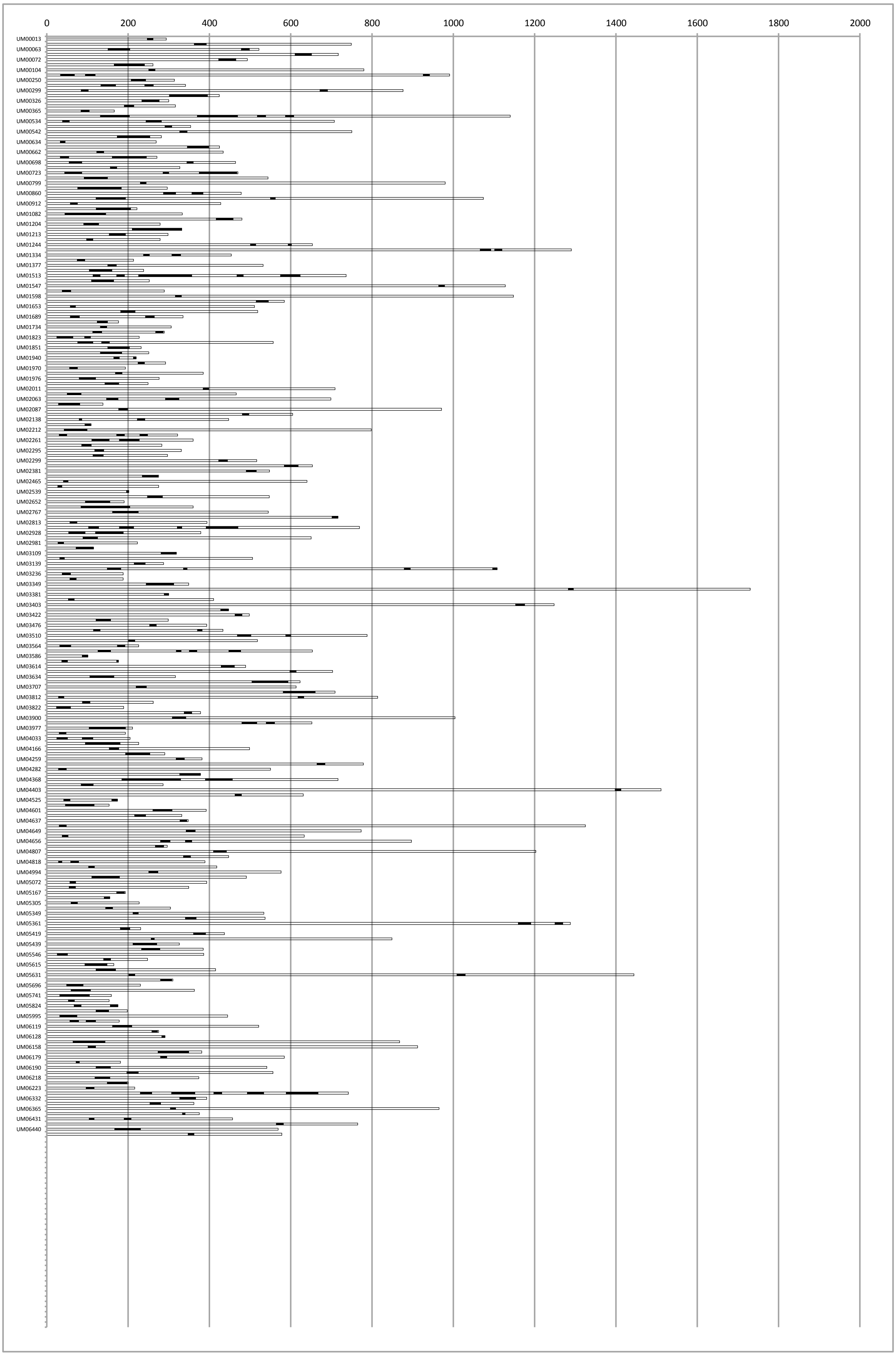

Supplement: Additional file 3 — Results of the search for pHGRs (predicted Hyper-O-glycosylated Regions) in the SignalP-positive proteins coded by the eight fungal genomes. [file 1471-2180-12-213-S3.pdf]
